# Genome-Wide Identification and Expression Analysis of *Dendrocalamus farinosus CCoAOMT* Gene Family and the Role of *DfCCoAOMT14* Involved in Lignin Synthesis

**DOI:** 10.3390/ijms24108965

**Published:** 2023-05-18

**Authors:** Lixian Wei, Xin Zhao, Xiaoyan Gu, Jiahui Peng, Wenjuan Song, Bin Deng, Ying Cao, Shanglian Hu

**Affiliations:** 1 Lab of Plant Cell Engineering, Southwest University of Science and Technology, Mianyang 621010, China; 2 Engineering Research Center for Biomass Resource Utilization and Modification of Sichuan Province, Mianyang 621010, China

**Keywords:** *Dendrocalamus farinosus*, *CCoAOMT* gene, genome-wide identification, drought response, lignin synthesis

## Abstract

As the main component of plant cell walls, lignin can not only provide mechanical strength and physical defense for plants, but can also be an important indicator affecting the properties and quality of wood and bamboo. *Dendrocalamus farinosus* is an important economic bamboo species for both shoots and timber in southwest China, with the advantages of fast growth, high yield and slender fiber. Caffeoyl-coenzyme A-O-methyltransferase (CCoAOMT) is a key rate-limiting enzyme in the lignin biosynthesis pathway, but little is known about it in *D. farinosus.* Here, a total of 17 *DfCCoAOMT* genes were identified based on the *D. farinosus* whole genome. *DfCCoAOMT1/14/15/16* were homologs of *AtCCoAOMT1*. *DfCCoAOMT6/9/14/15/16* were highly expressed in stems of *D. farinosus*; this is consistent with the trend of lignin accumulation during bamboo shoot elongation, especially *DfCCoAOMT14*. The analysis of promoter cis-acting elements suggested that *DfCCoAOMTs* might be important for photosynthesis, ABA/MeJA responses, drought stress and lignin synthesis. We then confirmed that the expression levels of *DfCCoAOMT2/5/6/8/9/14/15* were regulated by ABA/MeJA signaling. In addition, overexpression of *DfCCoAOMT14* in transgenic plants significantly increased the lignin content, xylem thickness and drought resistance of plants. Our findings revealed that *DfCCoAOMT14* can be a candidate gene that is involved in the drought response and lignin synthesis pathway in plants, which could contribute to the genetic improvement of many important traits in *D. farinosus* and other species.

## 1. Introduction

Bamboo forests, with about 1500 species worldwide, cover approximately 31.5 million hectares in tropical and subtropical regions of the world [1]. They help address the issue of global warming by fixing carbon dioxide in the atmosphere [2,3]. Moreover, bamboo resembles wood in appearance, weight, hardness, texture and strength, making bamboo an ideal substitute for many traditional wood products. *D. farinosus* is an important sympodial bamboo species for both bamboo shoots and materials; it is mainly distributed in southwest China, with the advantages of high fiber content, a large biomass, cold tolerance, rapid growth and high yield, which are also important for paper making and ecological greening [4].

Lignin is a complex polyphasic aromatic polymer that is not only a key product of the phenylpropane pathway, but also an important component of plant cell walls, playing an important role in enhancing plant mechanical strength and contributing to the defense against pests and diseases [5]. However, lignin is not conducive to paper making and lignocellulosic biomass fuel production. Lignin is mainly deposited in thick-walled tissues such as vessels and fibers of plants, and by firmly fixing cellulose and hemicellulose components in cell walls, it increases the difficulty to separate and utilize polysaccharide components such as cellulose [6,7]. Overall, lignin is essential for the growth and development of bamboo, resistance to pathogenic bacteria, timber properties of bamboo and quality of bamboo shoots.

The three main lignin monomers are p-hydroxyphenyl (H), guaiacyl (G) and syringyl (S), which are formed from phenylalanine by a multi-step redox catalysis through the phenyl propane metabolic pathway. The enzymes catalyzing lignin synthesis have been clearly identified [8]. CCoAOMT is a key methyltransferase in the lignin synthesis pathway and belongs to the methyltransferase family; it is closely related to plant lignin biosynthesis [9]. The alteration of the *CCoAOMT* gene causes the change of the ratio of S/G and affects wood material and forage digestibility. *CCoAOMT1*, the first identified true *CCoAOMT* family gene involved in lignin synthesis [10], affects plant resistance to *Pseudomonas syringae* DC3000 in *Arabidopsis* [11]; *AtCCoAOMT1* can also improve drought tolerance via ROS- and ABA-dependent signaling pathways [12]. Meanwhile, the overexpression of the *CCoAOMT* gene improved the resistance of plants to different pathogens [13,14,15]. For example, overexpression of the *Paeonia ostii CCoAOMT* gene in tobacco increased drought tolerance by enhancing lignin content and reactive oxygen species clearing capacity [16]. ZmCCoAOMT2 interacts with NLR Rp1 to regulate plant defense responses in maize [13]. More and more research suggests that CCoAOMT is important for the plant biomass and defense response. At present, the *CCoAOMT* gene family has been identified in *Arabidopsis thaliana*, poplar, tea tree, wheat, grape and other species [17,18,19,20], but little is known in *D. farinosus*.

Here, we analyzed the phylogeny, chromosome distribution, gene structure and motif composition of *DfCCoAOMT* family genes by using bioinformatics methods. We identified 17 *DfCCoAOMT* genes based on *D. farinosus* whole-genome data. Meanwhile, RNA-seq and qRT-PCR were used to analyze the expression pattern of *DfCCoAOMT* genes, combined with tissue sections to screen for candidate genes involved in lignin synthesis. *DfCCoAOMT* genes were involved in the ABA/MeJA signaling pathway in response to abiotic and biotic stresses. Overexpression of *DfCCoAOMT14* in tobacco promoted lignin biosynthesis and drought resistance of transgenic plants. Overall, this study laid a foundation for investigating *DfCCoAOMT* family genes in lignin biosynthesis and the drought response during plant growth and development.

## 2. Results

### 2.1. Genome-Wide Identification of CCoAOMT Genes in D. farinosus

Based on the reported protein sequence of the *Arabidopsis CCoAOMT* gene family and the methyltransferase structural domain from Pfam (PF01596), a total of 17 CCoAOMT genes were identified based on the *D. farinosus* genome and named as DfCCoAOMT1 to DfCCoAOMT17 (Table 1). Detailed information about these DfCCOAOMT gene sequences is presented in Supplemental Table S1 and Supplementary Document S1. These lengths of DfCCoAOMT proteins ranged from 220 aa (DfCCoAOMT13) to 311 aa (DfCCoAOMT17), the molecular weight ranged from 24.30 KDa (DfCCoAOMT13) to 34.00 KDa (DfCCoAOMT17) and the isoelectric point (pI) ranged from 4.88 (DfCCoAOM6) to 9.44 (DfCCoAOMT3). A subcellular localization prediction indicated that all CCoAOMT proteins were localized in the cytoplasm and chloroplasts.

### 2.2. Phylogenetic Relationship, Gene Structure and Conserved Motif Analysis of the CCoAOMT Gene Family in D. farinosus

**Figure 1:**
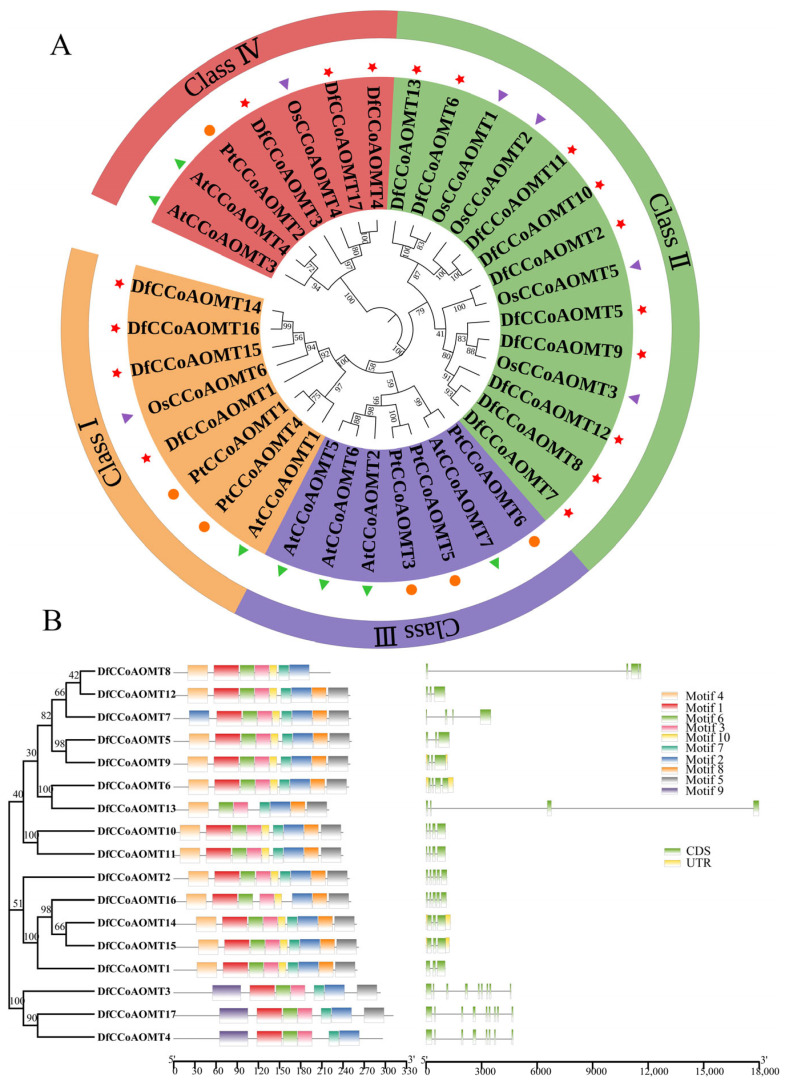
Phylogenetic relationship, conserved motif and gene structure of *D. farinosus CCoAOMTs*. (**A**) Phylogenetic tree construction based on amino acid sequences of the *CCoAOMT* family from *Arabidopsis thaliana* (At), rice (Os), *Populus trichocarpa* (Pt) and *D. farinosus*. The species are labeled with shapes of different colors: Df, (red pentagram); Os, (purple triangle); At, (green triangle); and Pt, (orange circle). (**B**) From left to right, phylogenetic tree constructed by MEGA analysis. The numbers beside the branches represent bootstrap support values from 1000 replications. Conserved motifs identified by MEME analysis. Each motif is represented with a different color. The protein length can be estimated using the scale at the bottom. Gene structures identified by GSDS analysis. Exons and introns are represented by boxes and lines, respectively. The sequence length of exons can be estimated using the scale at the bottom. The intron sizes are not to scale. CDS, coding sequence; UTR, untranslated region.

To clarify the evolutionary relationships and classification of the *CCoAOMT* gene family in *D. farinosus*, a phylogenetic tree was constructed using the full-length protein sequences of CCoAOMT from *D. farinosus*, *Arabidopsis*, rice and *Populus* with IQ-TREE v1.6.12 and the Model Finder algorithm (Figure 1A). The results showed that the *DfCCoAOMT* genes were divided into four groups (Classes I~IV); Classes I, II and IV contained 4, 10 and 3 *DfCCoAOMT* genes, respectively, and Class III had no *DfCCoAOMT* genes. However, in *Arabidopsis*, *AtCCoAOMT* genes were mainly clustered in Class III, only two genes (*AtCCoAOMT3* and *AtCCoAOMT4*) were clustered in Class IV and one (*AtCCoAOMT1*) was clustered in Class I. *DfCCoAOMT1*, *DfCCoAOMT14*, *DfCCoAOMT15* and *DfCCoAOMT16* were homologs of *AtCCoAOMT1*, *OsCCoAOMT6*, *PtCCoAOMT1* and *PtCCoAOMT4*, respectively. *DfCCoAOMT6* and *DfCCoAOMT9* were homologs of *OsCCoAOMT1* and *OsCCoAOMT3*, respectively, suggesting that they might have a similar function.

Next, the gene structure and conserved motif composition of *DfCCoAOMT* were analyzed (Figure 1B). The MEME online website was used to investigate the conservation and diversity of motifs in different DfCCoAOMT proteins. The results showed that all the proteins were found to be related to O-methyltransferase; motifs 2, 3, and 6 were common motifs in all proteins. Motif 1 was present in 16 DfCCoAOMT proteins except DfCCoAOMT13, and motif 9 appeared in 15 DfCCoAOMT proteins apart from DfCCoAOMT4/8. Motif 9 was only observed in Class IV, which might be associated with the functional diversity of *DfCCoAOMT* genes. Then, the exon–intron gene structure of *DfCCoAOMTs* were analyzed. The exon number of *DfCCoAOMTs* ranged from 3 to 9 and the intron number ranged from 2 to 8. Six genes (*DfCCoAOMT6/7/8/10/11/13)* contained four exons and three introns, and *DfCCoAOMT1/5/9/12/14/15* contained three exons and two introns; this difference in gene structure may be an evolutionary genetic difference. Meanwhile, the members of the same group had similar exon–intron structures and motif compositions.

### 2.3. Chromosome Distribution and Collinearity Analysis of DfCCoAOMT Genes

**Figure 2:**
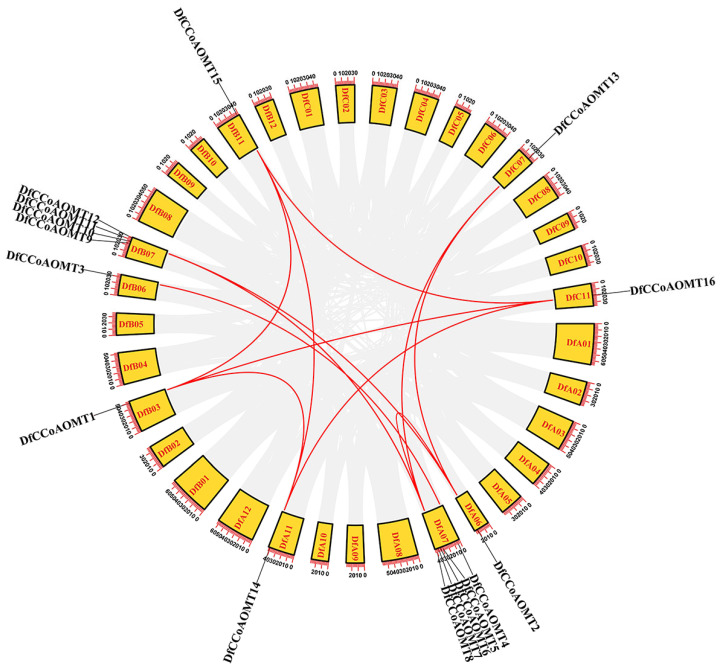
Chromosome distribution and collinearity analysis of *DfCCoAOMT* genes. The *DfCCoAOMT* genes are localized on different chromosomes. Chromosome numbers are indicated in the yellow boxes. The numbers on the chromosome boxes represent the sequence length in megabases. Gene pairs with sibling relationships are connected by a red line.

Gene shrinkage and expansion are important factors for plant gene functional diversity and environmental adaptation. Tandem repeats and fragment repeats are the main causes of gene expansion in plants. Therefore, the chromosome distribution and collinearity of the *DfCCoAOMT* gene family were analyzed (Figure 2 and Supplemental Figure S1). In *D. farinosus,* these *DfCCoAOMT* genes were located on chromosome groups 3, 6, 7 and 11, respectively; the *DfCCoAOMT* genes were most abundant on chromosome group 7 (10/17). Furthermore, there was an obvious tandem duplication in the seventh chromosome group, *DfCCoAOMT5/6/7/8* formed a tandem repeat gene cluster on chromosome A07 and *DfCCoAOMT9/10/11/12* formed a tandem repeat gene cluster on chromosome B07. Four pairs of putative paralogous *DfCCoAOMT* genes were identified and located on different chromosomes; these results suggested that there were fragment duplication events in the *DfCCoAOMT* gene family and led to expansion of the family members.

### 2.4. Tissue Expression Profile of DfCCoAOMTs and Analysis of Expression at Different Developmental Periods in D. farinosus

**Figure 3:**
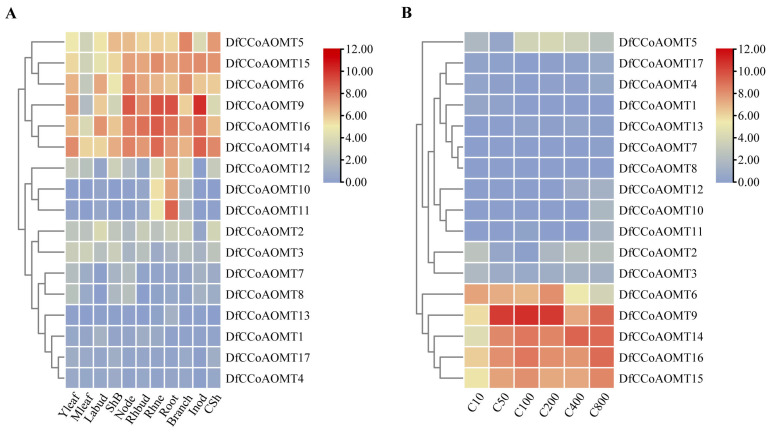
Expression analysis of *DfCCoAOMTs* in different tissues and developmental stages of *D. farinosus*. Hierarchical clustering analysis of expression levels of *CCoAOMTs* in different tissues. (**A**) Different developmental tissues of *D. farinosus*. (**B**) Different developmental phases of *D. farinosus*. The clustering was performed using TBtools. The red and blue colors correspond to the strong and weak expression of the genes, respectively. Dendrograms along the left sides of the heat map indicate the hierarchical clustering of genes. Young leaf (Yleaf), Mature leaf (MLeaf), Lateral bud (Labud), Sheath of bamboo shoot (ShB), Node, Rhizome bud (Rhbud), Rhizome neck (Rhne), Root, Branch, Internode (Inod) and culm sheath (CSh). C10 to C800 indicate bamboo shoots from 10 cm to 800 cm.

To better understand the function of the *DfCCoAOMT* genes in *D. farinosus*, we performed a transcriptome sequencing analysis of the expression levels of various tissues and different developmental periods in *D. farinosus* (Supplemental Table S3). As shown in Figure 3A, *DfCCoAOMT* gene expressions were different among the Young leaf (Yleaf), Mature leaf (MLeaf), Lateral bud (Labud), Sheath of bamboo shoot (ShB), Node, Rhizome bud (Rhbud), Rhizome neck (Rhne), Root, Branch, Internode (Inod) and Culm sheath (CSh). Among these 17 genes, *DfCCoAOMT10/11/12* were specifically highly expressed in the root, implying that they mainly functioned during root development. Eight genes (*DfCCoAOMT1/2/3/4/7/8/13/17*) showed a low expression in all tissues. *DfCCoAOMT6/9/14/15/16* showed a high expression level in various tissues, especially in the Inod, Rhne, root and node. These results suggested that *DfCCoAOMT* genes function in organ development and a variety of biological processes in *D. farinosus*.

Since lignin deposition and secondary wall thickening usually accompany rapid growth of bamboo, we then analyzed the transcript levels of bamboo shoots during different growth periods (Figure 3B). Only *DfCCoAOMT6/9/14/15/16* showed a high expression in fast-growing culm, indicating that these five genes might be involved in lignin synthesis during the rapid growth period of bamboo culms.

### 2.5. Prediction of Cis-Component Analysis of DfCCoAOMTs

**Figure 4:**
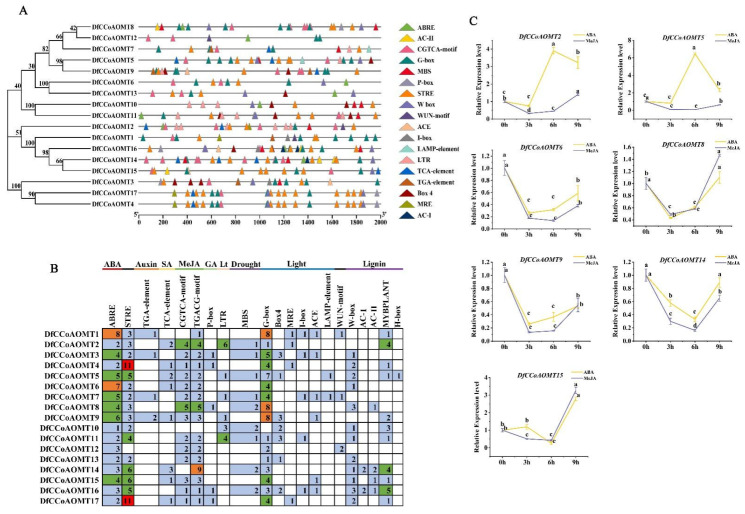
Predicted cis-element analysis in the promoter regions of *DfCCoAOMT* genes and the expression of *DfCCoAOMTs* under ABA and MeJA treatments in *D. farinosus.* (**A**) The phylogenetic tree of *DfCCoAOMT* gene family members. Putative cis-elements in *DfCCoAOMT* gene promoters. Promoter sequences (2 kb) of seventeen *DfCCoAOMT* genes were analyzed using PlantCARE and PLACE. Different color and shape boxes stand for different cis-elements. (**B**) Number and function of cis-elements. (**C**) Relative level of gene transcription of *DfCCoAOMT* in response to 100 mM ABA treatment and 100 mM MeJA treatment for 3 h, 6 h and 9 h, respectively. Error bars indicate standard deviation among three independent replicates. The values represent the mean ± SD; different letters indicate significant differences according to Duncan’s multiple range test and least significance difference (LSD) (*p* < 0.05).

To explore the mechanism of *DfCCoAOMT* genes in stress response and development, the cis-regulatory elements in the 2 kb upstream sequence of *DfCCoAOMT* genes were predicted by PlantCARE and PLACE (Figure 4A,B). In the *DfCCoAOMT* genes’ promoter, a variety of stress-, growth- and development-related cis-acting elements were identified, such as the low temperature response element (LTR), gibberellin (GA) response element, drought response element (MBS), light response element (G-box, Box4, MRE, I-box, ACE and LAMP element), MeJA response element (CGTCA-motif and TGACG-motif), salicylic acid (SA) response element and abscisic acid (ABA) response element (ABRE). Light- and growth-related cis-acting elements were present in almost all *DfCCoAOMT* promoter sequences. MeJA and ABA response elements were identified in the promoter of *CCoAOMT2/5/6/8/9/14/15.* Various lignin synthesis-related elements, such as MYB elements, were identified in the promoters of *CCoAOMT1/2/4/5/9/10/11/14/15/16/17*, especially *CCoAOMT14.*

We then examined the expression pattern of *DfCCoAOMT2/5/6/8/9/14/15* after ABA and MeJA treatments using qRT-PCR (Figure 4C). The results showed that the expression levels of *DfCCoAOMT6/8/9/14* decreased rapidly after ABA and MeJA treatments, reaching the lowest levels at 3 h and 6 h, respectively, and then gradually bound. In contrast, the expression levels of *DfCCoAOMT2/5* were significantly increased in response to ABA after 3 h, reaching the highest levels at 6 h. These results suggested that these *DfCCoAOMT* genes might be involved not only in lignin synthesis, but also in plant biotic and abiotic stress responses.

**Figure 5:**
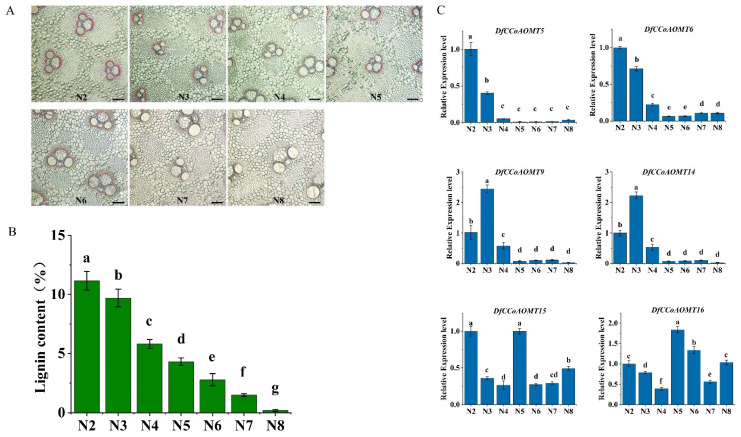
Shoot section diagram and lignin content determination of 2 m high bamboo shoots. (**A**) The sliced images of the 2 m high bamboo shoots from bottom to top; number 2–8 denote second to eighth internode; the slices were stained with phloroglucinol. Ten times magnification to observe sections, the scale bar = 100 μm. (**B**) The lignin content of bamboo shoots from the second to eighth internode. (**C**) The relative expression levels of *DfCCoAOMTs* in bamboo shoots from second to eighth internode. The values represent the mean ± SD, and different letters indicate significant differences according to Duncan’s multiple range test and least significance difference (LSD) (*p* < 0.05).

In addition, due to the differences in lignification between different internodes of bamboo shoots, the microscopy of individual stem sections of 2 m tall bamboo shoots was performed. Phloroglucinol-Cl staining showed a gradual decrease in internode lignin deposition from base to tip in bamboo shoots and measurements of lignin content in different internodes showed consistent results (Figure 5A,B). Furthermore, the expression levels of *DfCCoAOMT* genes in different internodes were detected and showed that *DfCCoAOMT5/6/9/14* had high expression levels in the first to third internode, consistent with the trend of lignin deposition. In contrast, *DfCCoAOMT15/16* were highly expressed in all internodes, suggesting that they are also involved in other biological functions of bamboo shoots (Figure 5C).

### 2.6. Overexpression of DfCCoAOMT14 Improves Lignin Biosynthesis in Transgenic Tobacco

**Figure 6:**
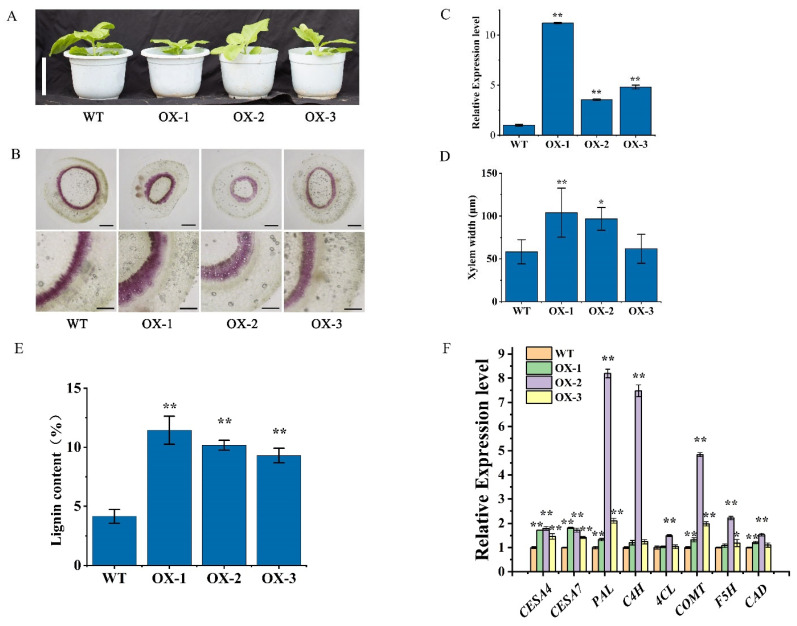
Overexpression of *DfCCoAOMT14* improves lignin biosynthesis in transgenic tobacco. (**A**) Phenotypic observation of WT and three transgenic plant lines. The scale bar = 10 cm. (**B**) Phloroglucinol-Cl staining of stem transverse section. The upper row images were observed using a 1.6-fold microscope, the scale bar = 250 μm, and the lower row images were magnified by 5 times, the scale bar = 100 μm. (**C**) Detection of the expression level of *DfCCoAOMT14* in overexpression tobacco and wild type. (**D**) Statistics of xylem widths of WT and three transgenic plant lines. (**E**) Determination of lignin content of the shoots. (**F**) Detection of expression level of secondary wall synthase genes including *CESA4*, *CESA7*, *PAL*, *C4H*, *4CL*, *COMT*, *F5H* and *CAD*. Data are means ± SD (*n* = 3). The asterisks indicate a significant difference compared to vector-transformed plants using the one-way analysis of variance (* *p* < 0.05, ** *p* < 0.01).

To determine the role of *DfCCoAOMTs* in lignin biosynthesis, *DfCCoAOMT14* was cloned and *CaMV 35S*-driven plant expression vectors were constructed and then transformed into tobacco. The expression of *DfCCOAOMT14* in transgenic plants and wild type was detected using qRT-PCR and we found that the expression levels of line 1, 2 and 3 were significantly higher than in wild type (Figure 6C). The phenotype of transgenic plants showed growth retardation compared with wild type (Figure 6A). In addition, the third internode of the 40-day-old transgenic plants was used to observe the anatomical cross-section; the results showed that overexpression of *DfCCOAOMT14* significantly increased xylem lignification in transgenic plants compared with wild type; the xylem widths in lines 1, 2 and 3 increased by approximately 46.5%, 9.2% and 12.3% compared with wild type, respectively (Figure 6B,D). Determination of lignin content in stems from transgenic plants also showed a significant increase (Figure 6E). Then, the expression levels of several lignin biosynthesis-related genes were detected using qRT-PCR and it was found that the expression levels of four genes (*PAL, C4H, CAD* and *COMT*) in transgenic lines were significantly higher than those of wild type (Figure 6F). These results suggested that the elevated expression levels of *DfCCoAOMT14* could enhance lignin biosynthesis in plants.

### 2.7. Overexpression of DfCCoOMT14 Improved Drought Resistance of Transgenic Plants

**Figure 7:**
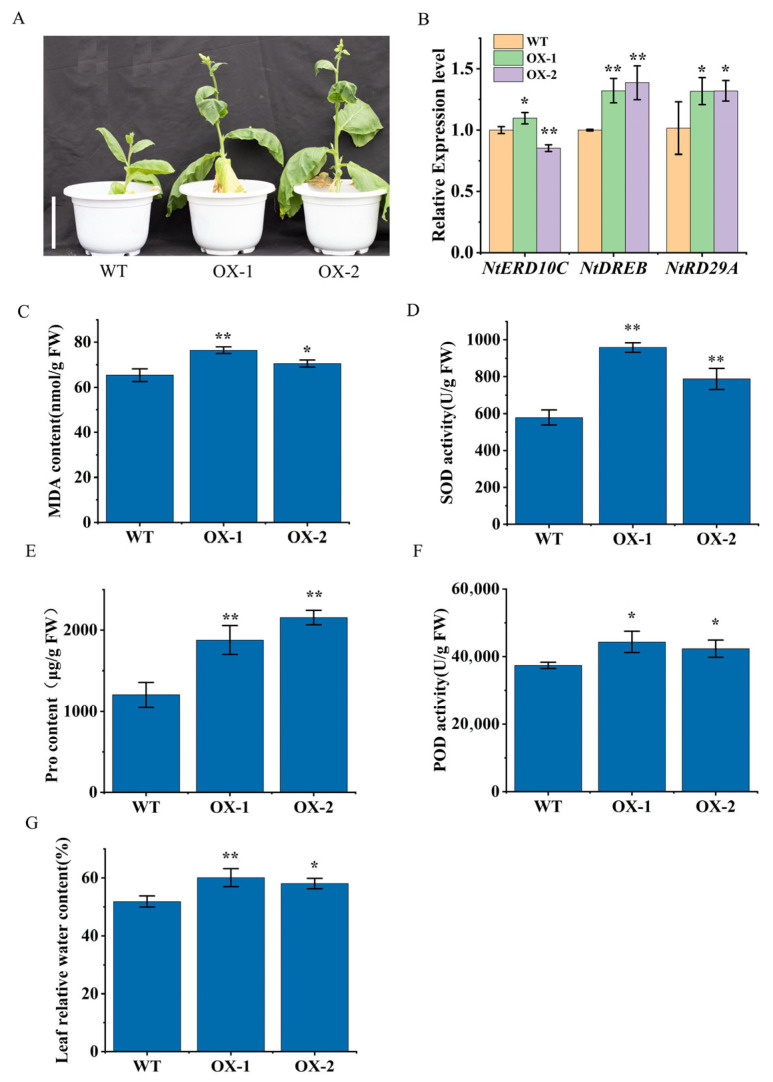
Overexpression of *DfCCoOMT14* enhanced drought resistance of plants. (**A**) Phenotype of wild type and transgenic plants after drought treatment. The scale bar = 10 cm. (**B**) Determination of the expression levels of drought-related genes including *NtERD10C*, *NtDREB* and *NtRD29A* in drought-treated plants. (**C**–**G**) The measurement of relevant physiological indices including MDA, SOD, Pro, POD and RWC. Data are means ± SD (*n* = 3). The asterisks indicate a significant difference compared to vector-transformed plants using the one-way analysis of variance (* *p* < 0.05, ** *p* < 0.01).

Previous studies have shown that lignin synthesis significantly affects the plant responses to drought [16]. We performed a drought treatment on transgenic plants for 37 days and found that the height and growth of transgenic plants performed better than wild type (Figure 7A). Moreover, we measured the drought-related physiological indicators in transgenic plants and wild type after the drought treatment; the levels of malondialdehyde (MDA), superoxide dismutase (SOD), peroxidase (POD), proline (Pro) and relative water content (RWC) in transgenic plants were significantly higher than in wild type (Figure 7C–G). qRT-PCR showed that the expression levels of the drought-related genes *DREB* and *RD29A* were also significantly higher in transgenic plants compared with wild type (Figure 7B). These results suggested that *DfCCoAOMT14* improved the drought resistance of plants.

## 3. Discussion

As a cross-linked phenolic polymer, lignin is not only a major component of plant cell walls, determining the degree of lignification, but is also involved in the plant’s ability to resist injury in response to abiotic and biotic stresses [21]. Lignin biosynthesis is a complex and delicate process; lignin monomers are synthesized from phenylalanine in the cytoplasm through a series of enzymatic reactions, and then transported across the membrane to participate in the synthesis of cell walls. As an important key enzyme for lignin synthesis, CCoAOMT catalyzes the methylation of caffeoyl-coenzyme A (caffeoyl-CoA) to feruloyl-coenzyme A (feruloyl-CoA), which mediates the biosynthesis of G-type lignin monomers to S-type lignin monomers, affecting the ratio of S/G lignin components [13]. We explored the number and function of *CCoAOMT* genes involved in lignin synthesis, which play important roles in improving fiber quality and lignocellulosic biomass utilization. The *CCoAOMT* gene family involved in the lignin biosynthesis pathway has been identified and analyzed in several species, including *Arabidopsis*, rice, tea tree, tobacco and poplar [22,23,24]. In this study, 17 *DfCCoAOMT* genes were identified based on the *D. farinosus* whole genome data, which is much more than the number of genes in *Arabidopsis* (7), poplar (6) and rice (6) (Table 1 and Figure 1A). According to the phylogenetic analysis of these CCoAOMT protein sequences from *D. farinosus* and other species, these *CCoAOMTs* could be clustered into four groups and no gene was present in Class III; *DfCCoAOMT1/14/15/16* clustered with *Arabidopsis CCoAOMT1* in Class Ⅰ, 10 genes clustered in Class II and 3 genes clustered in Class IV. Proteins clustered in the same group usually exhibit similar functions; *AtCCoAOMT1* has been shown to be the true *CCoAOMT1* gene and is involved in lignin biosynthesis, suggesting that *DfCCoAOMT1/14/15/16* in Class Ⅰ may also be primarily involved in this biological function [24].

In addition, the gene structure determines protein conformation and the protein structure determines function; genes with similar protein structures usually have similar functions (Figure 1B). According to our analysis, all DfCCoAOMTs have structural motif 2/3/6, which might be the key structure to maintain the functional conservation of this gene family. While motif 1 of the N-terminal region was also present in all the remaining DfCCoAOMTs except DfCCoAOMT13, since the protein sequences of the N-terminal and C-terminal regions are essential for enzyme activity and substrate binding, the deletion of motif 1 might lead to the specificity of the function of DfCCoAOMT13. DfCCoAOMT1/14/15/16 had the same protein structure, and the genes’ exon–intron distribution was also consistent except for DfCCoAOMT16. DfCCoAOMT3/4/17 had a specific structural motif 17, which might lead to a functional diversification of *DfCCoAOMT* genes. Gene duplication was considered to be a major factor leading to gene family expansion and gene functional diversity. Based on the chromosomal localization analysis of the *D. farinosus* 17 *CCoAOMT* genes, 9 *DfCCoAOMT* genes were found to be densely distributed on chromosome 7, showing local tandem and proximal gene duplication; this is similar to that in *Arabidopsis* [25] and *Populus* [26], which may be due to gene evolution. The other remaining *DfCCoAOMT* genes were scattered on chromosomes 3, 6 and 11 (Figure 2). In poplar, *CCoAOMTs* were mainly evenly distributed on chromosomes 1, 8, 9 and 10 [17]. Compared with *Arabidopsis*, rice and poplar, the *DfCCoAOMT* gene family was significantly expanded, which might lead to more secondary metabolites and enhance the adaptability of *D. farinosus* to the environment. Of course, it requires more research to prove.

Cis-regulatory elements in gene promoter regions are critical for regulation of gene expression patterns and transcriptional expression [27]. Different cis-acting elements usually represent that genes are involved in different biological processes. Previous studies have found that AC elements are commonly found in the promoter regions of lignin biosynthetic enzyme genes, such as *CCoAOMT, PAL, C4H, COMT* and *CAD,* and are important for the transcriptional regulation of the lignin biosynthetic pathway [28]. H-box elements were involved in the regulation of lignin biosynthesis [29]. The Mybplant motif in the promoter of the phenylpropanoid biosynthesis genes regulates lignin biosynthesis by binding P-box elements [30]. In our study (Figure 4), all *DfCCoAOMT* gene promoters, except *DfCCoAOMT3/6/7/12/13*, contained AC elements or Mybplant elements, especially *DfCCoAOMT2/14/16*, suggesting that these genes might be involved in lignin biosynthesis. Furthermore, other cis-acting elements were identified in the *DfCoAOMT* gene promoters, which were associated with ABA, auxin, SA, MeJA, GA, LTR, drought and light. It has been well documented that lignin synthesis is closely related to environmental stress and could help plants resist mechanical damage and pathogenic bacterial attack. In switchgrass, drought and injury treatments rapidly induced an elevated expression level of *PvCCoAOMT* [31]. In *Arabidopsis*, ABA regulated lignin synthesis by phosphorylating NST1 [32]. A MeJA treatment also rapidly increased the expression of lignin synthase genes (including *CCoAOMT*) to promote lignin synthesis [18,33]; the JA core transcription factor MYC2 could regulate secondary wall synthesis by regulating the expression of *NST1* [34,35]. Therefore, we sprayed ABA and MeJA on 1-year-old plants and then detected the expression of *DfCCoAOMT2/5/6/8/9/14/15*; we found that the expression levels of *DfCCoAOMT6/8/9/14* were significantly inhibited by ABA and MeJA and reached the lowest level at 3 and 6 h, respectively, while the expression levels of *DfCCoAOMT2/5* were rapidly induced in response to the ABA treatment and reached the highest level at 6 h (Figure 4C). These results suggested that ABA and MeJA signaling could regulate lignin synthesis by affecting the expression of *DfCCoAOMT* genes, and different *DfCCoAOMT* genes played different roles in response to ABA and MeJA, but how they coordinate environmental signals and lignin synthesis needs further investigation.

According to the analysis of transcriptome data from different growth periods/tissues of *D. farinosus* (Figure 3), the *DfCCoAOMT* genes had differential expression profiles in tissues. *DfCCoAOMT1/2/3/4/7/8/13/17* showed low expression levels in all tissues and different growth periods, suggesting that they were not involved in lignin synthesis during the normal growth and development of *D. farinosus*. *DfCCoAOMT10/11/12* were specifically highly expressed in roots, indicating that they were mainly involved in root development. *DfCCoAOMT6/9/14/15/16* showed a high expression in several tissues, especially in stems and roots, suggesting that they were involved in lignin synthesis in several tissues during plant growth and development. The elongation process of bamboo shoots is usually accompanied by secondary wall thickening and lignin accumulation. To further screen the key *DfCCoAOMT* gene in the lignin synthesis pathway [36,37], we analyzed sections of 2 m tall bamboo shoots from each internode and found that the lignin content gradually decreased from the base to the top of the bamboo shoots (Figure 5). qRT-PCR showed that the expression trends of *DfCCoAOMT5/6/9/14* were consistent with lignin accumulation, combined with the transcriptome data analysis, suggesting that these three genes might be the main enzyme genes involved in lignin synthesis during the development of *D. farinosus* shoots. According to our results, there were still a large number of *DfCCoAOMT* genes that might not be directly involved in lignin accumulation in normal development; they would be preferred to the synthesis of other secondary metabolites or participate in the stress response. For instance, *VvCCoAOMT4*, along with many *CCoAOMT* genes from other plant species [38,39,40], was shown to be involved in the synthesis of flavonoids, especially anthocyanins. Of course, whether the same function exists in *D. farinosus* requires further verification using transgenic technology.

Compared to *Arabidopsis* and *Populus*, the CCoAOMT gene family in *D. farinosus* has more members and more complex functions. *DfCCoAOMT14* might be a key gene that is involved in lignin synthesis in plants. To further validate our analysis, DfCCoAOMT14 was overexpressed in tobacco (Figure 6) and we found that the transgenic plants were slightly smaller than wild type, but the lignin content in stems was significantly increased and the xylem width was thickened. qRT-PCR confirmed that the expression levels of genes related to lignin synthesis were significantly up-regulated, indicating the function of *DfCCoAOMT14* involved in lignin synthesis. Drought stress experiments confirmed that overexpression of *DfCCoAOMT14* significantly improved the drought resistance of plants (Figure 7); this result is consistent with previous studies of *Pinus sylvestris CCoAOMT* [16]. Moreover, the content of lignin is crucial to the properties and quality of bamboo, which is a major limiting factor for bamboo pulp papermaking. Mutation of the *CCoAOMT* gene in *Arabidopsis* significantly down-regulated lignin content, especially G-type lignin [10]. Reverse repression of *CCoAOMT* in alfalfa significantly reduced lignin content but increased the ratio of S/G lignin components, which facilitated lignin degradation and separation [41]. So far, changing the expression levels of the key enzyme genes in the lignin biosynthesis pathway is still the main means to regulate lignin content. Therefore, this study can provide a theoretical basis for improving the wood properties of bamboo and breeding superior bamboo species with a high biomass or low lignin.

## 4. Materials and Methods

### 4.1. Identification of CCoAOMT Genes of D. farinosus

For the identification of the *CCoAOMT* family in *D. farinosus*, the methyltransferase domains (PF01596, CCoAOMT) were downloaded from Pfam (http://pfam.xfam.org, accessed on 1 May 2023) and they were used as a query in Hidden Markov Model (HMM) searches for candidate *CCoAOMT* genes in *D. farinosus*, with the cutoff at 0.01 [42].The Conserved Domain Search (https://www.ncbi.nlm.nih.gov/Structure/cdd/wrpsb.cgi, accessed on 1 May 2023) online software in NCBl was used to remove the sequences without the methylation domain. The online tool ExPASY (http://web.expasy.org/protparam/, accessed on 1 May 2023) was used in calculating the molecular weight (MW) and theoretical isoelectric point (pI) of DfCCoAOMT proteins. The subcellular localization of DfCCoAOMT proteins was predicted using Cell-PLoc 2.0 (Cell-PLoc 2.0 package (sjtu.edu.cn, accessed on 1 May 2023)). A total of 17 *CCoAOMT* family members were retained in *D. farinosus* for further analyses (Table 1). The candidate *CCoAOMT* gene sequences of *D. farinosus* were identified from the whole-genome data of *D. farinosus*, and all *DfCCoAOMT* gene sequences are attached in Supplementary File 1.

### 4.2. Phylogenetic Analysis and Sequence Alignment of the CCoAOMT Family of D. farinosus

The *CCoAOMT* family protein sequences of *Arabidopsis thaliana*, rice and *Populus trichocarpa* were downloaded from Phytozome (https://phytozome.jgi.doe.gov/, accessed on 1 May 2023) (Supplemental Table S1). Phylogenetic trees were constructed with IQ-TREE v1.6.12 using the Model Finder algorithm. We performed multiple sequence alignment analyses of CCoAOMT protein between *D. farinosus* and other species [43]. We constructed a phylogenetic tree between the CCoAOMT genes of *D. farinosus* by using the Clustal W program in MEGA 7.0 software and the neighbor-joining (NJ) method with 1000 bootstrap replications [44].

### 4.3. Chromosomal Localization and Collinearity Analysis of DfCCoAOMT Genes

The internal collinearity relationship was analyzed by using the Multiple Collinearity Scan toolkit (MCScanX) and we obtained collinearity gene pairs in the *D. farinosus CCoAOMT* family genes [45]. The collinearity analysis maps were then constructed using the Dual Systeny Plotter software (https://github.com/CJ-Chen/TBtools, accessed on 1 May 2023) [44]. Then, the Ka, Ks and MYA values were calculated using WGD v1.1.1 (Supplemental Tables S4 and S5).

### 4.4. Conserved Motifs and Gene Structure Analysis of CCoAOMT Family of D. farinosus

The conserved motifs of *DfCCoAOMTs* were determined using the MEME online tool (version 5.3.0, http://meme-suite.org/tools/meme, accessed on 1 May 2023) [46]; the number of motifs output was 10. The motif analysis and gene structure were visualized using the TBtools software. The Gene Structure Display Server 2.0 online tool (GSDS, http://gsds.gao-lab.org/index.php, accessed on 1 May 2023) [47] was used to illustrate the structure and exon/intron organization of *DfCCoAOMT* genes and the genomic length. The 2000 bp upstream promoter sequences of *DfCCoAOMT* genes were submitted to the PlantCARE database [48] (http://bioinformatics.psb.ugent.be/webtools/plantcare/html/) and PLACE (https://www.dna.affrc.go.jp/PLACE/) to predict cis-elements and to subsequently screen cis-elements manually.

### 4.5. Plant Materials and Hormone Treatment

All materials used in this study were from the Institute of Bamboo Research, Southwest University of Science and Technology. One-year-old young leaves of *D. farinosus* were treated with 100 mM ABA and MeJA, respectively, and sampled in liquid nitrogen before treatment and after 3 h, 6 h and 9 h of treatment.

### 4.6. RNA Extraction and qRT-PCR

Total RNA of *D. farinosus* leaves was extracted using the TRIzol reagent (Takara, Dalian, China). The RNA quality was monitored using 1% denaturing agarose gel and a NanoDrop 2000 spectrophotometer (Thermo Fisher Scientific, Beijing, China). Then, cDNA was synthesized from 1 to 2 μg total RNA using the FastKing cDNA First Strand Synthesis Kit (TIANGEN, Beijing, China). The specific primer used for qRT-PCR was designed by using Primer Premier 5.0 (Supplemental Table S2). *Tubulin* was used as an internal reference gene. The transcript levels of *DfCCoAOMT* were obtained using the SYBR qPCR Master MIX kit (Vazyme, Nanjing, China) on the CFX96TM Real-Time System thermal cycler (BIO-RAD, CA, USA). The relative expression levels of *DfCCoAOMT* genes were calculated with the 2-ΔΔCt method [49]. Each sample had three independent biological replicates—each with three technical replicates.

### 4.7. RNA Sequencing

RNA sequencing was performed on 1-year-old *D. farinosus* materials in a growing chamber. The samples of *D. farinosus* included Young leaf (Yleaf), Mature leaf (MLeaf), Culm sheath (CSh), Sheath of bamboo shoot (ShB), Lateral bud (Labud), Node, Root, Branch, Internode (Inod), Rhizome bud (Rhbud), Rhizome neck (Rhne) and shoots of different height. The analysis included three biological duplications. Total RNA was extracted by using the TRIzol reagent (Takara, Dalian, China); the quantity and quality of total RNA were detected by a NanoDrop 2000 spectrophotometer (Thermo Fisher Scientific, Beijing, China). Seventeen samples were sequenced by the NovaSeq 6000 platform (Illumina, Beijing, China). The clean reads of each sample were compared with the reference genome of *Dendrocalamus farinosus*, and the alignment rate ranged from 74.47% to 91.57%. The raw reads were further processed with a bioinformatic pipeline tool, the BMKCloud (www.biocloud.net, accessed on 1 May 2023) online platform. All of these RNA-seq data were mapped to the *D. farinosus* reference genome using HISAT2 and the fragments per kilobase per million (FPKM) were calculated using StringTie. The heatmap was generated by TBtools based on the transformed data of log_2_ (FPKM + 1) values. The details of the sample information and the FPKM values of CCoAOMT genes are shown in Supplemental Table S3, respectively.

### 4.8. Overexpression of DfCCoAOMT14 in Nicotiana Tabacum

Overexpressed tobacco lines were obtained by introducing the pCAMBIA1302-*DfCCoAOMT14* with the CaMV 35S promoter recombinant vector into the *Agrobacterium tumefaciens* strain EHA105 using the leaf disc method [50]. Infected tobacco leaf discs were inoculated on a MS medium containing 0.1 mg/L NAA, 0.5 mg/L 6-BA, 400 mg/L cephalexin and 9 mg/L Hyg in the dark at 27 ± 1 °C for 2 d. The regenerated buds were transferred to a MS medium containing 0.1 mg/L NAA, 400 mg/L cephalexin and 9 mg/L Hyg for the formation of complete plants. Genomic DNA was extracted from the leaves of transgenic plants and wild type and then amplified under the following conditions: preheating at 95 °C for 5 min, followed by 30 cycles of denaturation at 95 °C for 30 s, annealing at 60 °C for 30 s, extension at 72 °C for 1 min and finally extension at 72 °C for 10 min (Supplemental Figure S2).The PCR product was checked using 1% agarose gel electrophoresis. The expression of *DfCCoAOMT14* in transgenic plants was detected by qRT-PCR and *N. tabacum NtActin* was used as the internal references for normalization [51].

### 4.9. Lignin Analysis

For plant cell wall preparation, the second to eighth internodes of 2 m bamboo shoots were ground to powder in liquid nitrogen. The determination method of lignin content was based on the method of Li et al. [52] and was appropriately improved as follows: weighed 0.5 g fresh sample, added liquid nitrogen to grind to fine powder, added 5 mL 0.1 M phosphate buffer (pH 7.2), incubated at 37 °C for 30 min and centrifuged for 5 min. Then, added 5 mL 80% ethanol to incubate at 80 °C for 1 h, centrifuged for 5 min and added 10 mL acetone to extract once, centrifuged for 5 min and dried at 60 °C. For the thioglycolic acid lignin analysis, approximately 20 mg of dried plant cell wall was incubated at 80 °C in 750 μL of water, 250 μL of concentrated HCl and 100 μL of thioglycolic acid for 3 h. The mixture was centrifuged and the pellet was washed with 1 mL of water and resuspended in 1 mL of 1 M NaOH on a rocking plate at room temperature overnight. The mixture was centrifuged again, and the supernatant was collected and mixed with 200 μL of concentrated HCl. After being vortexed and incubated at 4 °C for 4 h, the mixture was centrifuged and the pellet was dissolved in 1 mL of 1 M NaOH. The absorbance of a 50-fold dilution of the supernatant in 1 M NaOH was measured at 280 nm. The lignin content of *Nicotiana tabacum* overexpressing *DfCCoAOMT14* was also determined by the same method.

### 4.10. Histochemical Staining

The complete and thin sections were stained in 2% phloroglucinol solution for 5 min, then were stained with 30% hydrochloric acid for 1 min, washed with water, observed and photographed under a Leica microscope.

### 4.11. Drought Treatment in DfCCoAOMT Transgenic Tobacco

In total, 45-day-old transgenic tobacco plants and wild-type plants were treated in drought stress for 37 days. The treated plants were then subjected to the measurement of relevant physiological indices, including malondialdehyde (MDA), superoxide dismutase (SOD), proline (Pro), peroxidase (POD) and leaf relative water content (RWC). The fresh weight (FW) of tobacco leaves was weighed. The fresh tobacco leaves soaked in distilled water for 24 h in the condition of 16 h light/8 h dark at room temperature and the turgid weight (TW) was weighed. The leaves were then dried for 24 h at 80 °C to obtain the total dry weight (DW) [53]. Leaf relative water content (RWC) was estimated according to the method of Turner (1981) [54]: RWC (%) = (fresh weight − dry weight)/(turgid weight − dry weight) × 100. MDA content, Pro content, SOD activity and POD activity were all measured using the related kit (Solarbio, Beijing, China). Subsequently, qRT-PCR was used to detect the expression levels of drought-related genes including *NtERD10C*, *NtDREB* and *NtRD29A* in drought-treated plants [55]; tobacco *NtActin* served as an internal reference.

### 4.12. Statistical Analysis

The data in this study were statistically analyzed by using SPSS 25.0 and Origin 2018. Data are presented as means ± SD of three independent replicates. The one-way analysis of variance (ANOVA) was used for the significant difference analysis and the significant difference of *p* < 0.05 was statistically significant.

## Figures and Tables

**Table 1:** Characteristics of the *CCoAOMT* genes identified in *D. farinosus*.

Gene ID	Gene Name	Protein Length (aa)	MW (KDa)	pI	Subcellular Localization
DfaB03G023550	*DfCCoAOMT1*	240	28.33	5.41	Cytoplasmic
DfaA06G007370	*DfCCoAOMT2*	249	26.75	5.33	Cytoplasmic Chloroplast
DfaB06G012070	*DfCCoAOMT3*	293	32.08	9.44	Chloroplast
DfaA07G002980	*DfCCoAOMT4*	296	32.24	8.35	Chloroplast
DfaA07G014340	*DfCCoAOMT5*	252	27.58	4.95	Cytoplasmic
DfaA07G014350	*DfCCoAOMT6*	248	27.03	4.88	Cytoplasmic
DfaA07G014530	*DfCCoAOMT7*	251	27.85	6.45	Cytoplasmic
DfaA07G014550	*DfCCoAOMT8*	222	24.33	5.16	Cytoplasmic
DfaB07G011900	*DfCCoAOMT9*	250	27.31	5.09	Cytoplasmic
DfaB07G011910	*DfCCoAOMT10*	240	26.75	5.56	Cytoplasmic Chloroplast
DfaB07G011930	*DfCCoAOMT11*	240	26.63	5.78	Cytoplasmic Chloroplast
DfaB07G012160	*DfCCoAOMT12*	250	27.52	5.83	Cytoplasmic Chloroplast
DfaC07G006610	*DfCCoAOMT13*	220	24.30	4.88	Cytoplasmic
DfaA11G016070	*DfCCoAOMT14*	262	28.95	5.22	Cytoplasmic
DfaB11G015250	*DfCCoAOMT15*	260	28.93	5.33	Cytoplasmic
DfaC11G007040	*DfCCoAOMT16*	259	28.86	5.33	Cytoplasmic
Dfa0G042980	*DfCCoAOMT17*	311	34.00	9.09	Chloroplast

## Data Availability

The data which supports the findings of this study are linked in https://www.ncbi.nlm.nih.gov/, PRJNA923443.

## Supplementary Materials

The following supporting information can be downloaded at: https://www.mdpi.com/article/10.3390/ijms24108965/s1.

## References

[1] Guo L., Sun X., Li Z., Wang Y., Fei Z., Jiao C., Feng J., Cui D., Feng X., Ding Y. (2019). Morphological dissection and cellular and transcriptome characterizations of bamboo pith cavity formation reveal a pivotal role of genes related to programmed cell death. Plant Biotechnol. J..

[2] Sohel M., Alamgir M., Akhter S., Rahman M. (2015). Carbon storage in a bamboo (Bambusa vulgaris) plantation in the degraded tropical forests: Implications for policy development. Land Use Policy.

[3] Paudyal K., Adhikari S., Sharma S., Samsudin Y.B., Baral H. (2019). Framework for Assessing Ecosystem Services from Bamboo Forests: Lessons from Asia and Africa.

[4] Imran M., Luo X., Hu S., Cao Y., Long Z. (2022). Epigenetic and somaclonal divergence in Dendrocalamus farinosus for physiological augmentation and lignin degradation. Biotechnol. Appl. Biochem..

[5] Barros J., Serk H., Granlund I., Pesquet E. (2015). The cell biology of lignification in higher plants. Ann. Bot..

[6] Vanholme R., Demedts B., Morreel K., Ralph J., Boerjan W. (2010). Lignin biosynthesis and structure. Plant Physiol..

[7] Kang X., Kirui A., Dickwella Widanage M.C., Mentink-Vigier F., Cosgrove D.J., Wang T. (2019). Lignin-polysaccharide interactions in plant secondary cell walls revealed by solid-state NMR. Nat. Commun..

[8] Vanholme R., De Meester B., Ralph J., Boerjan W. (2019). Lignin biosynthesis and its integration into metabolism. Curr. Opin. Biotechnol..

[9] Zhang G., Zhang Y., Xu J., Niu X., Qi J., Tao A., Zhang L., Fang P., Lin L., Su J. (2014). The CCoAOMT1 gene from jute (Corchorus capsularis L.) is involved in lignin biosynthesis in Arabidopsis thaliana. Gene.

[10] Do C.T., Pollet B., Thévenin J., Sibout R., Denoue D., Barriere Y., Lapierre C., Jouanin L. (2007). Both caffeoyl Coenzyme A 3-O-methyltransferase 1 and caffeic acid O-methyltransferase 1 are involved in redundant functions for lignin, flavonoids and sinapoyl malate biosynthesis in Arabidopsis. Planta.

[11] Kwon H., Cho D.J., Lee H., Nam M.H., Kwon C., Yun H.S. (2020). CCOAOMT1, a candidate cargo secreted via VAMP721/722 secretory vesicles in Arabidopsis. Biochem. Biophys. Res. Commun..

[12] Chun H.J., Lim L.H., Cheong M.S., Baek D., Park M.S., Cho H.M., Lee S.H., Jin B.J., No D.H., Cha Y.J. (2021). Arabidopsis CCoAOMT1 Plays a Role in Drought Stress Response via ROS- and ABA-Dependent Manners. Plants.

[13] Wang G.F., Balint-Kurti P.J. (2016). Maize Homologs of CCoAOMT and HCT, Two Key Enzymes in Lignin Biosynthesis, Form Complexes with the NLR Rp1 Protein to Modulate the Defense Response. Plant Physiol..

[14] Yang Q., He Y., Kabahuma M., Chaya T., Kelly A., Borrego E., Bian Y., El Kasmi F., Yang L., Teixeira P. (2017). A gene encoding maize caffeoyl-CoA O-methyltransferase confers quantitative resistance to multiple pathogens. Nat. Genet..

[15] Xia Y., Liu J., Wang Y., Zhang X., Shen Z., Hu Z. (2018). Ectopic expression of Vicia sativa Caffeoyl-CoA O -methyltransferase (VsCCoAOMT) increases the uptake and tolerance of cadmium in Arabidopsis. Environ. Exp. Bot..

[16] Zhao D., Luan Y., Shi W., Zhang X., Meng J., Tao J. (2021). A Paeonia ostii caffeoyl-CoA O-methyltransferase confers drought stress tolerance by promoting lignin synthesis and ROS scavenging. Plant Sci..

[17] Zhao H., Qu C., Zuo Z., Cao L., Zhang S., Xu X., Xu Z., Liu G. (2022). Genome Identification and Expression Profiles in Response to Nitrogen Treatment Analysis of the Class I CCoAOMT Gene Family in Populus. Biochem. Genet..

[18] Lin S.J., Yang Y.Z., Teng R.M., Liu H., Li H., Zhuang J. (2021). Identification and expression analysis of caffeoyl-coenzyme A O-methyltransferase family genes related to lignin biosynthesis in tea plant (Camellia sinensis). Protoplasma.

[19] Yang G., Pan W., Zhang R., Pan Y., Guo Q., Song W., Zheng W., Nie X. (2021). Genome-wide identification and characterization of caffeoyl-coenzyme A O-methyltransferase genes related to the Fusarium head blight response in wheat. BMC Genom..

[20] Lu S., Zhuge Y., Hao T., Liu Z., Zhang M., Fang J. (2022). Systematic analysis reveals O-methyltransferase gene family members involved in flavonoid biosynthesis in grape. Plant Physiol. Biochem..

[21] Xiao Y., Li J., Liu H., Zhang Y., Zhang X., Qin Z., Chen B. (2020). The Effect of Co-Transforming Eucalyptus urophylla Catechol-O-methyltransferase and Caffeoyl-CoA O-methyltransferase on the Biosynthesis of Lignin Monomers in Transgenic Tobacco. Russ. J. Plant Physiol..

[22] Hamberger B., Ellis M., Friedmann M., de Azevedo Souza C., Barbazuk B., Douglas C.J. (2007). Genome-wide analyses of phenylpropanoid-related genes in Populus trichocarpa, Arabidopsis thaliana, and Oryza sativa: The Populus lignin toolbox and conservation and diversification of angiosperm gene familiesThis article is one of a selection of papers published in the Special Issue on Poplar Research in Canada. Can. J. Bot..

[23] Xu Z., Zhang D., Hu J., Zhou X., Ye X., Reichel K.L., Stewart N.R., Syrenne R.D., Yang X., Gao P. (2009). Comparative genome analysis of lignin biosynthesis gene families across the plant kingdom. BMC Bioinform..

[24] Rakoczy M., Femiak I., Alejska M., Figlerowicz M., Podkowinski J. (2018). Sorghum CCoAOMT and CCoAOMT-like gene evolution, structure, expression and the role of conserved amino acids in protein activity. Mol. Genet. Genom..

[25] Kim B.G., Kim D.H., Hur H.G., Lim J., Ahn J.H. (2005). O-Methyltransferases from Arabidopsis thaliana. Agric. Chem. Biotechnol..

[26] Barakat A., Choi A., Yassin N.B., Park J.S., Sun Z., Carlson J.E. (2011). Comparative genomics and evolutionary analyses of the O-methyltransferase gene family in Populus. Gene.

[27] Hernandez-Garcia C.M., Finer J.J. (2014). Identification and validation of promoters and cis-acting regulatory elements. Plant Sci..

[28] Lacombe E., Van Doorsselaere J., Boerjan W., Boudet A.M., Grima-Pettenati J. (2000). Characterization of cis-elements required for vascular expression of the cinnamoyl CoA reductase gene and for protein-DNA complex formation. Plant J. Cell Mol. Biol..

[29] Patzlaff A. (2010). Characterisation of a pine MYB that regulates lignification. Plant J. Cell Mol. Biol..

[30] Tamagnone L., Merida A., Parr A., Mackay S., Culianez-Macia F.A., Martin R.C. (1998). The AmMYB308 and AmMYB330 Transcription Factors from Antirrhinum Regulate Phenylpropanoid and Lignin Biosynthesis in Transgenic Tobacco. Plant Cell.

[31] Liu S.J., Huang Y.H., Chang-Jiu H.E., Fang C., Zhang Y.W. (2016). Cloning, bioinformatics and transcriptional analysis of caffeoyl-coenzyme A 3-O-methyltransferase in switchgrass under abiotic stress. J. Integr. Agric..

[32] Liu C., Yu H., Rao X., Li L., Dixon R.A. (2021). Abscisic acid regulates secondary cell-wall formation and lignin deposition in Arabidopsis thaliana through phosphorylation of NST1. Proc. Natl. Acad. Sci. USA.

[33] Sehr E.M., Agusti J., Lehner R., Farmer E.E., Schwarz M., Greb T. (2010). Analysis of secondary growth in the Arabidopsis shoot reveals a positive role of jasmonate signalling in cambium formation. Plant J. Cell Mol. Biol..

[34] Zhang Q., Xie Z., Zhang R., Xu P., Liu H., Yang H., Doblin M.S., Bacic A., Li L. (2018). Blue Light Regulates Secondary Cell Wall Thickening via MYC2/MYC4 Activation of the NST1-Directed Transcriptional Network in Arabidopsis. Plant Cell.

[35] Luo F., Zhang Q., Xin H., Liu H., Yang H., Doblin M.S., Bacic A., Li L. (2022). A Phytochrome B-PIF4-MYC2/MYC4 module inhibits secondary cell wall thickening in response to shaded light. Plant Commun..

[36] Chen M., Guo L., Ramakrishnan M., Fei Z., Vinod K.K., Ding Y., Jiao C., Gao Z., Zha R., Wang C. (2022). Rapid growth of Moso bamboo (Phyllostachys edulis): Cellular roadmaps, transcriptome dynamics, and environmental factors. Plant Cell.

[37] Yang K., Li L., Lou Y., Zhu C., Li X., Gao Z. (2021). A regulatory network driving shoot lignification in rapidly growing bamboo. Plant Physiol..

[38] Liu X., Zhao C., Gong Q., Wang Y., Cao J., Li X., Grierson D., Sun C. (2020). Characterization of a caffeoyl-CoA O-methyltransferase-like enzyme involved in biosynthesis of polymethoxylated flavones in Citrus reticulata. J. Exp. Bot..

[39] Nakamura N., Katsumoto Y., Brugliera F., Demelis L., Nakajima D., Suzuki H., Tanaka Y. (2015). Flower color modification in Rosa hybrida by expressing the S-adenosylmethionine: Anthocyanin 3′, 5′-O-methyltransferase gene from Torenia hybrida. Plant Biotechnol..

[40] Widiez T., Hartman T.G., Dudai N., Yan Q., Lawton M., Havkin-Frenkel D., Belanger F.C. (2011). Functional characterization of two new members of the caffeoyl CoA O-methyltransferase-like gene family from Vanilla planifolia reveals a new class of plastid-localized O-methyltransferases. Plant Mol. Biol..

[41] Guo D., Chen F., Inoue K., Blount J.W., Dixon R.A. (2001). Downregulation of caffeic acid 3-O-methyltransferase and caffeoyl CoA 3-O-methyltransferase in transgenic alfalfa. impacts on lignin structure and implications for the biosynthesis of G and S lignin. Plant Cell.

[42] El-Gebali S., Mistry J., Bateman A., Eddy S.R., Luciani A., Potter S.C., Qureshi M., Richardson L.J., Salazar G.A., Smart A. (2019). The Pfam protein families database in 2019. Nucleic Acids Res..

[43] Nguyen L.-T., Schmidt H.A., von Haeseler A., Minh B.Q. (2015). IQ-TREE: A Fast and Effective Stochastic Algorithm for Estimating Maximum-Likelihood Phylogenies. Mol. Biol. Evol..

[44] Chen C., Chen H., Zhang Y., Thomas H.R., Frank M.H., He Y., Xia R. (2020). TBtools: An Integrative Toolkit Developed for Interactive Analyses of Big Biological Data. Mol. Plant.

[45] Wang Y., Tang H., Debarry J.D., Tan X., Li J., Wang X., Lee T.H., Jin H., Marler B., Guo H. (2012). MCScanX: A toolkit for detection and evolutionary analysis of gene synteny and collinearity. Nucleic Acids Res..

[46] Bailey T.L., Williams N., Misleh C., Li W.W. (2006). MEME: Discovering and analyzing DNA and protein sequence motifs. Nucleic Acids Res..

[47] Hu B., Jin J., Guo A.Y., Zhang H., Luo J., Gao G. (2015). GSDS 2.0: An upgraded gene feature visualization server. Bioinformatics.

[48] Lescot M., Dehais P., Thijs G., Marchal K., Moreau Y., Van de Peer Y., Rouze P., Rombauts S. (2002). PlantCARE, a database of plant cis-acting regulatory elements and a portal to tools for in silico analysis of promoter sequences. Nucleic Acids Res..

[49] Livak K.J., Schmittgen T.D. (2001). Analysis of relative gene expression data using real-time quantitative PCR and the 2(-Delta Delta C(T)) Method. Methods.

[50] Zhang H., Gao X., Zhi Y., Li X., Zhang Q., Niu J., Wang J., Zhai H., Zhao N., Li J. (2019). A non-tandem CCCH-type zinc-finger protein, IbC3H18, functions as a nuclear transcriptional activator and enhances abiotic stress tolerance in sweet potato. New Phytol..

[51] Wang C., Wang L., Lei J., Chai S., Jin X., Zou Y., Sun X., Mei Y., Cheng X., Yang X. (2022). IbMYB308, a Sweet Potato R2R3-MYB Gene, Improves Salt Stress Tolerance in Transgenic Tobacco. Genes.

[52] Li Y., Kim J.I., Pysh L., Chapple C. (2015). Four Isoforms of Arabidopsis 4-Coumarate:CoA Ligase Have Overlapping yet Distinct Roles in Phenylpropanoid Metabolism. Plant Physiol..

[53] Hu W.E.I., Huang C., Deng X., Zhou S., Chen L., Li Y.I.N., Wang C., Ma Z., Yuan Q., Wang Y.A.N. (2013). TaASR1, a transcription factor gene in wheat, confers drought stress tolerance in transgenic tobacco. Plant Cell Environ..

[54] Turner N.C. (1981). Techniques and experimental approaches for the measurement of plant water status. Plant Soil.

[55] Liu W., Xiang Y., Zhang X., Han G., Sun X., Sheng Y., Yan J., Scheller H.V., Zhang A. (2019). Over-Expression of a Maize N-Acetylglutamate Kinase Gene (ZmNAGK) Improves Drought Tolerance in Tobacco. Front. Plant Sci..

