# Supplementary material for: Genome-Wide Identification and Expression Analysis of Dendrocalamus farinosus CCoAOMT Gene Family and the Role of DfCCoAOMT14 Involved in Lignin Synthesis
Source: Int J Mol Sci. 2023 May 18;24(10):8965. doi: 10.3390/ijms24108965 (PMC10219071; doi:10.3390/ijms24108965)
Supplement: Supplementary file 1 [file ijms-24-08965-s001.zip › supplementary document S1-The candidate CCoAOMT gene sequences of D. farinosus.pdf]

Supplementary Document S1 The candidate *CCoAOMT* gene sequences of *D. farinosus*

>DfCCoAOMT1 (DfaB03G023550)
TACAACTTCTGTGCCAGGTTAGAAATCATAACAGCACGTTTCAAATATCCATTAGTTGG
GACTTTGCACTGCTGTTTGTAGAATGCCTTAATTTTTGACACTTTGGTGAACATATTTGA
CTTCTTACTGACCTTCCCTTTGCTCCTTCAATAGGACAGCAGTCAAAATCTTTTTGGCA
GGAAACGCCCATAGTGAGTGCTGATTGTTTCGATAGAGGTTCACTCAGGAAAGAACAA
TCACAAGATATGAAGCCAAAAAACACAGAAAGTGTACTGTTCATGACGCACTTGCATA
GGTAAGTTTCCTTTTTGGCACGTATGCCTTTAAAGGTGGGAGATGTTTTTATGTCAGTA
ATAGCTGCCCTTTGATTGTAAGCATTTTTTACCAAGTTAGTGGTTTGAAGGATGATCTGT
CTGGTATTAGTTCATATTAGTTGAAGGAAGGTTTGTTCGTTCAAGCTAGACTTCCTTATA
TTCTGGAAAAGGCATTAACCTGTTTGAGGGGCTCCCAGAAGCTTAAGTTCAAGTTTGA
TTTTTTGCCTTGAAAAAAAGTTATTGATTTTGATTTACTAGTAAAGTTTCCTAGATTATG
CTAGCTTCTAACCTATGTGAAATCGATGAAACTATTCCACAGCCATCTTGCCACTTTCCA
TGGAACAGTCTACTTTAGGTTTCTTAAGTTACACAAAACAGTATTCAGCTATACATCATC
CCTCCGCTCTTTCTCAAATTAGTATGTGATATGCAGCATATGCTCAGTCCTATACTCCAG
AATTCTGATGCCTCTACTTTTTAAGGTCTTGATTACCCCACATTAGTTGACAGTTCTTCT
CTTTCCAATCCTGGACAGCTTGCAGAGCGGTGACACTGAAGCAGGTCTGAGGACAGG
AACAAGGTAACCTTATAATCAGATGGGCTTTCTGAATGATTTGCCCATTGGGGTGCTTC
GTTTTGGCGGTGCAGAGCAGAGGCTATGATAATAGGAAAGTAAAGTAGTGGTGATCAT
CAGATGCACCTTGTCTTGTACTAGCTAGCAGTTAAATCAGTCAGATTGTACTCGGATGT
GAAATGTTCTGTAAAGCGACAGCTCAATCTATGAGCTGCCAACGACAGCAAATATATCC
GCTGTTACGAGATAGGGAGATGTCAATCAGTCATATAAATAGTACTGGTGTATTAAGGC
CATAGGGTGACCACATTTTGGTGGGAGCTGGAACACTGGGTTACAAGGCCATCTCCAA
TCCCACTGCTGTAATGAACGCAATCTAGCGACCATGCATATGCTAGCAGTAATTTAGGG
ATGAACCTGGAAGTGCTTGTTTCTTCATTATGCAGCACGTCTTCTTCTTTTTTTAATGGG
AGTGTCTACACAACATTCGACTGTTTTTTAGGACAAAGGCAGTCAGATTTTGAGCCGTT
AGATGGAGATCCAACGTCCTAGATTATTTTTTTTAGAGGGGTCATTTTTTTTAGGGGGTC
TTGTATGGATGGATCCGTTGGATGGAGATCTAACGTTCCAGATCAATTTGACTGTTTTTA
TCCTCAAAAACAGGCAATGTTGTATAGCAATACTCTTTTTAAATTTGGTCCTAGTGGCA
GCGAGAAAATGCGCCGATGACCGTGAAGCACGTGAAGCTACCCGGCCCTGAGCGCGT
GTCACGGAGGGAGGCAGAGTACGTCCGCCGGCGCGCCTCCATGCACGAGCTCACCCG
CCGTCGCCGTCGCCGCCACCGCACCCGCCCCCACCTTCCACCGTCGTGAGTGCTCAGC
TCGGTGCATGCGAAGCATGGCACCACCACCCCGCGCCACTTGTTTGATGCCCTCGCCA
ACAATCCACTGCACATGCACATACACCAAACCGCTCTAATCTCGCCTTCCTTTCCACGT
ATGTGATATATGTCCCAGCCCACGGATTAACAGCAGCCAGCCACGAGATCGAGTGAGG
AATATCGCGCGCACGTACGATATGGCCAGCGCCGACGGCGAGTCCGCGGGGGCGC**ATG**
CAGAGCAACGGCGGGGAGGAGCAGACGCGGCACCAGGAGGTCGGACACAAGAGCCT
CCTCCAGAGCGACGCGCTCTACCAGGTGACGATTTGATAGATCGTTCGCTGCAAAACA
GCTAGCGTGATTGCCCGTTCTGCTCTCTGATCGGTCTGCTCCAAGCTGATGGTTTGATAT
GCGATTGGTCTGCAGTACATCCTGGAGACGAGCGTGTACCCGCGCGAGCCGGAGTGCA
TGAAGGAGCTCCGCGAGATCACCGCCAAGCACCCATGGTGAGTGGCCGTGCACCACC
TGTGCGTGCGTCTCGCCTGACGATCAATTCTGTGGCTGATTTGTGCGAAATCTTCGCTT

CCCTTTGCTGCAGGAACATCATGACGACGTCGGCGGACGAGGGGCAGTTCCTGAACAT
GCTGCTCAAGCTCACCGGCGCCGAGAAGACCATGGAGATCGGCGTCTACACCGGCTAC
TCCCTCCTCGCCACCGCGCTCGCCATCCCACAAGACGGCACAGTTAGTAGTAGTCCGT
AGTACCCACTGCCCACATGCACACTCTTTCGATCTTGACCACAAGGCTCATGGCATCCG
TGCACGCGGATCCTGGCCATGGACATCAACCGGGACAACTACGAGCTGGGCCTGCCGT
GCATCGAGAAGGCCGGCGTCGCCCACAAGATCGACTTCCGCGAGGGCCCTGCCCTCC
CCGTCCTTGACCACCTCCTGGAGGACGTGCGTAATACATTAAAAATCATGCATCCATCC
ATCAATTGATTTGATGCTAACGAACAATGTGCAATGATCTGTGCGCACGCAGGAGAAG
AACCTGGGGTCGTTCGATTTCGTGTTCGTGGACGCGGACAAGGACCTCAACTACCACG
AGCGGCTGATGAAGCTGGTGAGGGTTGGCGGCCTCGTCGGCTACGACAACACGCTCT
GGAACGGCTTCGTCGTGCTCCCCGCCGAAGCGCCCAAGCGCAAGTACATCCGCTACTA
CCGCGACTTCGTGCTCCAGCTCAACAAGGCCCTTGCCGACGACGACCGCGTCGAGATC
TGCCAGCCCCCCGTCGGCGACGGCATCACGCTTTGCCGCCGAGTCAAG**TGA**

>DfCCoAOMT2 (DfaA06G007370)
AAGAAAATGCACTGAAAATAATAAAATGACAGCAATAATACCATTTTTCAGCCGGCCCT
AAAAAGATTGGCCCAACAAAAAATGCGTAAAAGAAATGGCCCAGCTCGGGGCCCAAA
AAACGAAATCGCGCAAGCGGGCCCAACATTTCACAGACGCGCGCGCAGCCCGGTCGG
AAAACGCGCGCGGCGCGTGACCTGAAATGCCCGTACGGGCCAAAATCGTCAGCGAGG
CCCAATGATCCCGCGTCTTACCTGCGCGCGCCGTCGGATGAAATCTAACGGCCGTAAG
AGCACGCGGCAGGCATTAAGTCCCCGAAACCCTAACCCTAATCATTTGCACCCGGTTCT
TCCCGAAAATACCGGAGCGGCGACAGCTCGCGCGCTCGCGCGGAGAGCGGCGGCGGC
GGCTCTACATGGCGGCATCGGCGGCAGCCCCGCGCGCACCGGCGACGGCCCCACGCG
CAATGGCGGCGCTCGGGCGGCACCGGCGAAGCCCCAGCGCGAAGCAAGGCGGCGCG
CAGCGACCCCGAGTGGAGCAAGGCAGCGCGCGGCGACGGCGGCGGCGCCCGCACGG
AGCGGCGGCAGCGTGAGGCGGCGGCGGCCCCGCAGCCGACGGTAGCGGCGCCCGCC
GAACGGGCGGTGACGGCGGCAGCGGCGGCGCCCAAAAAAAAAGGCCGTAAGTCCGA
TCCATTTTTTTTTTGTTCTTGGTTTGCCAGCGCCGATCTAATTTCTTTCGGGTGAATTGG
GTTTTTAGGGGACTTACATTAGGGTTTGCAAAATAAAATCAAATCACCTCCTTCTAAAT
GCTAAGCCGAATCGTTAACAAAATTAAATCCGAGCCTCCAAAACCAGACCAAAAATCG
GGTTGGGTAATAAGGGTTCATGATGAGCTAGGGGTGCTCTGATACCAAATGTTAGGTTT
AATCTAAGTCTAAAACATTAACAATAGCCTTAACCCTAGATCAAAGGCATGAACACCTA
GCCAAAACATCACTGCGGAAGCGTCATGAACATACCCGAAGTTGAGGCGGCCATCTTG
AGAGGTCCTCGGTTGGTGCGGACGGAGGAGGTCGGCGTCGGTGAAACGGAGGTCGCC
GGCGTGGACGACGGAGCCGCGGACGGAGATGAGTTTCGGCCTTCGTTCACCACAACG
CCCATGATAGATCGGTTCTTGTTAGGGTTAGAGAGACTTAGGACAACGCGTGTGTTTGT
CTTTTCCGAAACCCTCGTTTTTATATGGCGCTGGGTGACCGGGACCGAAACCAAACGG
GTTGGTTGCCGTCTCCGATCAAGACGTGCGAGTGGAGGCGTGCCTCCCTGAAAACTCG
GAGTTGGTTAGCCGAGATCAACCCAACATTAACCACTTACATATGCGGATGGAAAGAAT
TGACAAAGCGCCTGTTCATACCAACAACTGATGTCTCTTTGGTACTATTTAAGTCTTACC
CGAGAGAGAAGAAACAATACAACATAACACAAGACAGAACAAACAGCAAGTTCATTC
CGCATTAGGATAAGTAGTATTTTTCCTGTAATTGTTAATTTAGAGCAAGTTGTAATCTTC
ATATAGCGACCAATTTTTTTTCATTCTTCTGTGAGGCTCAAATCTCTCTATTATTGGTAAG
ATTGACGACTCAATCGTCGACATCATGTCTCAAATGTATTTCCCTTGTATGACTCTGAAA

TCAAAAGAAATTTTGACAAAATACATTTCTTAGTTAAAAAGTGCGGAAGTTTATGGCAC
TCATTCACAGCTTAAAACTTACACATAGATCATACCCCTTATGTCTTATTTAAGGACGAT
CTTAGATGTTGGCATGTTGCATCAGGATTCAGGCATGACCAAACCTTCAGCCAAGATAT
TATTCTATGAAGAAAAAAAGCGTTCGCTAAAATATTTTAACTGATGGTGATGGATACCA
GATGGCAAGGACTAGGCGTCTTAGGTGCTGCGATCGGCGGGGCTTCTTGTTTTTTGCTT
TTTTACTTCTGCGTAGGAGA**ATG**GCCGCCGCCGACGCCGGAGAAGGGAAGAAGACGG
CCGCCGGAAGCAGCCTCCACAGCAAGACCCTCCTCAAAAGCGAGCCACTGTACCAGG
TAAACCATCTGAAGCACCAATATCCCCACGCGGCACGCGCCCAAAGGCCTTCACCTGA
CAAGGCTAACGCTTCTGTCTTCCGTTCCCTGACGTTTCTCTTCCAGTACATACTAGAATC
CACGGTGTTCCCTCGCGAGCCAGACTGCCTGCGCGAGCTCCGCCTCGCCACCGCCAA
GCACCCCATGTAAGGCTTGCTCGATTCGGTGCTGCTTTTGTGTGTTGGGTCAGTGCACG
GCTGGTGTTTGACGCTTTGTCCGTACGGGTGAGGCGTGCTGCGCAGGGCTCGCATGGC
GGCCTCGCCGGACCAGGTGCAGCTGTTTGGGCTTCTGATCGAGGTGCTCGGCGCCAGG
AACGCCGTCGAGGTCGGCGTGTTCACCGGGTACTCGCTGCTCGCCACCGCCCTGGCGC
TCCCTGACGACGGCAAGGTTAGTATCTCTAATACTTGCTAGTCTGTTTAGGTGTATGTAT
GATCTTGGCTTGATCTCCGTGCTCGACGGCAGATAGTAGCCATCGACGTTAGCCGAGAG
AGCTACGACGAGATAGGATCGCCGGTGATCGAGAAGGCCGGCGTGGCGCACAAGATC
GACTTCCGCGTGGGGCTCGCGCTGCCGGTGCTGGATCAACTGGTAGCCGAGGTACACT
ATTCCTCCGCGTTCGTGTGCGCGTCCGGCGTCCCTGTGCAAATGCGCAGCGCAACGAG
ATGATCGTGATGTGGCCACGGCTGGCTCGACTTGCAGGAGGGCAACAAGGGCAGGTT
CGACTTCGCGTTCGTGGACGCGGACAAGGTGAACTTCCCCAACTACCACGAGCGGCT
GCTGCAGCTGGTCAGGGTCGGGGGCCTCATCGCCTACGACAACACGCTGTGGGGCGG
TTCCGTGGCCGTGTCCGCCGACGAGCCGCTCTCGGAGAGGGACCGCGCGCTCGCGGG
GCTCGCCAGGGAGTTCAACGCTGCAATCGCCAACGACCGCCGCGTTCAGGTGTGCCA
GCTCGCGATCTCCGACGGGGTCATGCTGTGCCGCCGCGTCGCC**TGA**

>DfCCoAOMT3 (DfaB06G012070)
ACAGTTACTGGCTTTTAAAAGAAATATCTTTTTAAGGCGAGGTCTACAAGTTGATTATA
GTACATACTGTAGCGACTTCATTTATATTGGTAAAAACCGCACACGAGTTTATGTTACTC
CGTACTTTTCCTCACGAACAGTCCAAAAGCCATAAGGCACAACCAAAGCTATAAAACC
GAAGCAAAAGGAACACACCCTATATCTACCCCTGTAGTTGTAATTGCCCATGTCTACAT
GAAGTCCAACAAAGCCTAATTAGTTCATCTATCTTAGGAATAGCTTAAAAAAGTCATGC
CAATTAATTTACAACGATGTAGACTACCTTAAATTTGTTGATATAGACCACTTCAAATCT
ATCCACCGTGCGTGTGCTTATGATCACATAGTATTCGATAAAAAAAAGTGCGCACTATCT
TGATAGGCATCTCATTAATTTTTCTCACTTTTTTTTGAGACAAATGTCGATAAATGGTTG
TAATATATACATTTAACCATTATGTCTATAGCACACTACATTAATAATAACTATAGAAAATG
ATTTTAAGTACCTTCGATAAACTGCAGCGATGTTAATTATTTTGACGTCACAAACCTGAT
TTTTTGGTGTGTACTTCCTCGCAAAAAATTATTATTATGCATATTAACTTTTGAAGTTTCT
AAAGTTTGCCTAGACATTAGAAAGCATCTATATATACCGGTGAGATCGATGGCAAGAAA
TTCATACATCCAAAATATTAAGCATTTGGTGTTGACTAAGGGTACATTCGAATAGACTTG
TGACTTATGGCTTTTCATTTCTTCTATAGTAACTACCTTTTAAACGACGTGTTTTTTAAGA
TTGAGTCTGCTAGTTAATTGTAGTATATAATGTACTAACTTTAGCTATACCGACTAAAATC
ATATACTACTATATCTTTTTTCGTATTTAAAAATCAAAAGCTACGGTCAAAAAACCACCT
TTAATCTGAGTGCACGCGTGCTTACTATGATGCATCCAGATACTAATTTGACTCTTGCAT

CATCCGTCGTACTACTTGTGTACGTATCTGCTCCAACGCACAAGGACAAAAACAAATG
CAACACCTTCTCCTTGTTTTAAAATAAATAAATAAAAAGGCGGGGTCTGACACGTTGCT
TAGATAAATAAATATTCAGGTTGTAGCTCAGTTGGTGAGCCGCATCCGCACCATGCTTG
CTCAAGAGCCGTACCGGACAATGACACGACTCAGTGTCTTCCTCGGCGAGGTCGCGTA
GAATACAGGCAGGAGCCAGGGACCGGCTTGTAATTTTCTTTACTTCCAAGGCTCCTTCT
GTAATTTCTGTGGTATATACTCTTGTTGTACTTTAGGGGCTAGTTGCCTAAAAAAAAGTC
GCGTGATCAGATAAGTCGTGTGCCAGTGCTTGTTTGATATGTGCCATAACTAATTGTTTC
CTTGTCTTCGATCAACTCGGCTTCAGCTTCTGCCTTCTGGCTTATTTTTTACTATTTATAA
GTTTCGTCTTTTTCTGAACTTTTGGCTTTTTGTTGAGTTTTTTTGCCGGTTCTTTTTTTCC
CGTATTTGTTTGTATTATACGTAATGGGCTAACATATTTTTATATTTTTAAATTTTTTAAAA
TTTAATTATGCTAAATAAGCCTCTGTCAGCTACAGTGTTTTCAGCCATAAGCCATGAGCT
TTTCAGCCGAACCAATGACCACCTTAACAAAAGTTAATATGCTTCCAGCATGTCATGCA
AGTAAGCAGATCGCCGTATATAAGACTTGCTTGAAAGTTGAAACACATGAAGTTCTCTG
GAATCCTGCGTAAATTTATTTTTGGAACACATGAATTTCGCTAGAATTCTATGTAAATTTT
ATAAAACACACAGTTTGAGTTATAAAGAAAAAAAATTCCCATACTTCTCGGGCCTAGA
GTGTCCGCACCTCACTGACATGTGGGCCCCGCAGACGGCAAGCTAATCTGCGTTTCTG
TTTCCACTTGGCGGCTGCCTCCGGCGACG**ATG**CCTCGCTTCGCCGTGGCCTCCTCCTCC
CTTCCTCCGTCGGTGGCGGCTGCTGCTGCTCTCGGGTTCGGCTCCAGGCATCCTCGCGT
CCTCCAATCCTTAACCCTAGCCGCCGCTCCGCCCCGCCGCGGCTCTCCGCCATGGAGCC
GCCGCCTGTGCATGTGCTCCTCCTCATCCTCCTCGACAGCGGCGGCGGTGGAGGAGGC
ACGGCGCGGGCGGAAGCAGCTGGGAATGACGCCGCAGCTCTACGGCTACCTCCTCGC
CAACGTCCGCGAGCACCCCGTGTGCCCCTCGCCCTCTCTCACTCTTCTGTTTCGTTTTG
AGCCAATTTGGGGAAATTTTGCTAAACTCTGGCCACCTCATGGATGCTTTTTAGATCCT
TCGGGAGCTCCGCCAAGAGACAGCGGCCATGAGAGGAAGCCAGATGCAGGTACATTA
GCATACATAAACTTAAAGAGTACTACTCTTTAGAATTTAGATGGAGGGTATTCGTTTTGT
TGCATTGTGTCCATAAGGTGTATTTTTGGGATAACTCTATCGTCCGTTCAAGTGTCAAGA
CTCGAAACATCCGATGCGCTAATTATACTGCAGCTCCAGTGCGGCATTTGCGATTTGCA
GTTTATCAACATTGCTGGGTTTTCTCAATGCGCCATGGAACGGGCTGGCACTTGTTAGC
ATGTTGCATGCTTTCATGGACATTCTGGGTATATAGGGATTGCATGCGTATAATGCTGTCT
TTTGATGATACGCATTGTATTGCTTCTACAGCTAGGTCTTTTTGAGAGTTTTTACTATTAT
TAGAAGGTAACCTTACGACCAAGTCACATATTTATCAATAATATGTGATTTGATGGTAAA
AACTATCTTTTTTTGGAGACATTATGTCCAAATCATTATGCGAGGATGAGTCTACTATTCT
TTTAGTGCCATTTTCTCGCTCTTCCCTAGAACAGTCTTAATTTTTTTCCTAGGGACATAC
CGAAAAGAAATCCATTTGTACATATTTATAATTTGTTTTCTGAAAATATAATGATAGTCTT
TGGATACGTTACCAAAATTCTGTATAATGGTAATCACTTTCAGCCGGTTGCTTTGTCTTG
GTAGGTTTCTCCCGCACAGGCTCAGCTGCTAGCGATGCTTGCACAAATTCTTGGGGCA
CAGCGATGCATTGAAGTTGGCGTCTATACTGTATGTGAACCTAGTGTTTTGTAATTTAGA
TTTTCCTACCATCCACTCTCAGTCAGTAAACACTGAACAGTACTGAGTTATTGCTTCTAC
TTGGAACCAAAGCATCCTACATTGATGTTATATTAGTTGTTGATGTGTTGTCCTAGCTCA
CGAAATATAGAGTTCCAAAAACCGGAACCGGAAGAAATCCCGGTCGGTGGCCGGTAA
AGGTAACCGGGCGGTTACCGGAAAAACCGGTCACAATTTGAAAAATCAAAATTTGAAT
TTTGAGATGTTTTGAAACCGGTAACCGGAAAACCCAGAAAAAACAGGTCAAAATTCG
AAAATCAAAAATTGCTACCGGCCGGTTTTTTCGGTTACCGGTTGCCACCGGTAAAAAC
GGGGCCGGTAACCGCCGGGATTTTGAACCCTGGTCCACACATATGTGCGAGAAAAATA

AGTATACATGAGTGGGTTAACATATCATCTGTGACATCTATGGTATCTATTGTTTGCCCTG
GAAGTGATACCATAATGGGCACTTCATATTTTCAAAGCACACAAATGGTACTTCAGAAT
TCTGAAGCCTGAACGTTGCACATATTTATGAGAGCCGTGTAAGAGCATGTACAATGCAG
GCGCTTAGAGAGGTGCATTACTGTTTCCGTCAGGGACAATAATGTCATGCATGATGTGA
ATCCGTCATATACGCATGCATGCATGCATCCATATGTGCTTACTGTTTAGTAATGGTGATA
CCTCCATGATGTAGCGGACCCATATGCGGCTGCATTGTACATGCTCTTACAGTAATGTTT
GTGTCAACTTGTCAAGTGCTGCGATGATTAGTGATCATTCTAGACCTTGTGAAGGAGCT
AATTTTGTACGAAAGCAACAAGCATTGCAAATTCACTAACCATATCTTAGCACTCCTTT
AATCTTTTTACAGGGATATTCATCATTAGCTGTTGCACTAGCTCTTCCTGAATCAGGTCG
TTTGGTTGCTTGTGAAAGGGACGAAAGATGTCTTGAAGTTGCCAAGAAATACTATCAG
CGTGCTGGTGTTGCACACAAGGTGATTATTTTCGGCCCTTTTTGCTTGATGAGATCGGT
TGATCATAACCAATGATTCTGAACTACATAATTTTCTATATTTGTACTTTACTTCTCTATCC
TTGGCTATAAATTTACAATTAATTGTGTGTGATGTTTCAGTAGGTTGAAGAACTTTTTTG
GTGCAAAGATTAATTCTCCCAACAATATAAATCATGGGAAAAATAATTTTTCAGAAGCA
GGGCAAAATCTTCTTTTTAGTTTCACCAATTTCCCCTTTATGTCTTTGTTATTTTACACCA
TTGTTCTTAAATGGTTCCACTTTTACTGACATATGTAGCACACTACAGTTCATGAAATTA
GGAATGCAACCACTTAATACATTGCATTTGTTTTGTAGGGGATCTAATGTGTGTAGGCTT
GATCAATATGTATTTCCTCTTTAATGATTTCTAAGCACTTAAAACTGAAACGTTGACCTT
TTTATGGAATCGCCTTGAATGTATAGAATATTAGTTTTCAAGGACCAGTTTGTATTCTTG
CATATTATATTTTCTATGCTGATCTGTCATTCTGTCTTAAAATTGCTTAGGTTTATGTCAAG
CACGCTTTAGCTGCAGATTCCCTAAGGTCATTGCTCGATTGTGGTGAAGCTTCCAGGTA
TAGTAATTTTAGTGTGGTATAATCCCCGTCATCATATTAGTTGACATCATACATGTTCTAA
CTAATCCTTTCCTTTTTCTGTTTTTTCCCCTAGTTATGACTTTGCATTCGTTGATGCAGAC
AAGAGAATGTATGAGGAATATTTTGAACTTCTACTTAAGCTAGTAAGATTCACCATGCA
GCTTATCTTCTGGGTTATTTATAACTCATTCTAGTCTATAACACATGTGGATGTGGTCCTC
CACTCTCTGTTCCATAGTGTTTTCTCATGATAATGTAGAAGATCCCCCATTCTTGCCACG
AGTTTTACGATTTTATCTATAATTTCCGCACATGATGATCATAGCATTTGTGTTTTTTTATA
TGTTGAGCAATACATCTTGTTGTGTTGTAGGTAAGAGTTGGTGGTTTAATTGTAATGGA
CAATGTCCTTTGGTATGGAAGGGTTGCTGATCCGCTAGTAAGTTCTTCTTTCTTTTTGAG
CTTTGACATGATAAAGATGTTACAGGGTAGACATTAGATAATACTTCTCTGGCATTTTTT
CTTAGGTGAATGATCCAAAGACAATCAGTATAAGGAACTTCAATAAGAGACTTTTGGA
GGATAAACGTGTCAATATCAGCATGGTCTCTTCCTCTCTCCACCTCTCATATGCTTGGTC
ATTGCACAGTTATTAGTCATTACTTGCGAATGTTGATTGTGAAGCCTTAAAAAATCTTTA
TATTTCTGCATAATTATTCTGTCGTCTGGAGTATGCAAAGAGCACCAACAGTGATGTGTA
GTAGCGGTGTTGCAATGCCAGCACAATCAAGGTTCCAAAAACAGGAACCGGAAGAAA
TCCCGGTCGGCGGCCGGTAACGGTAACCGGGCGGTTACCGGAAAAACCGGTAAAAAA
TCGGTCACAATTTGAAAATCAAAATTTATATTTTGAGATATTTTGAAACCGGTAACTGGA
AAAAAAACCGGCCAAAATTCGAAAAATCAAAAAATTTATACCGGCCGGTTTTTTGGTT
ACCGGTCTCCACCGGTAAAACCGGTAACCGGCGGTTACCGGCCGGTAACCGGCGGTTA
CCGGCCGGTAACCGCCGGGATTTTGAACCATGAGCACAATACATATCCCAAGTTCAAA
ATTTTTATTTGCCTAATATTGTGGCACATTCAAGCCATGGTCATGTATACCTGTTCGGATG
GGGAATTTGGATTCGAATCCGTAGGATTTAGTTCAAATCTGCAAGAATTTGAACCAAAT
CCGAAAAATTCACAAAGAAATCCCCTTCATCCAAACACTCCCTAAATGTGTAGAGTCC
TCTTCACTATCTTTATACATGGTCAACTGCTTCAAAACCTTTATACCCCCTTGTATCCAA

ACCTTCTTTTGATATACCGTTGACCATCCGTTTGGTCTATGTACAACTTCTGTGAGAAGA
AAACAGTATGCGTAGCATTGCTCCGATTACAAGTGTACTCTTGCAACCAATATGCAAAT
GAGAACCATAATATTTGTATCACTTAGATTTTCATTTGATACGCTTTTACCATGATTCAGT
GCCTCAATATCCCATGCCCTTCTCATGTGTTATATGTTCATACCATCTGTATGTGCTTAAG
TACTGGATCTTGATTAACAATGGTGTTATATTTTCGTGAGATTTGCAGGTGCCAATTGGG
GATGGGATGACAATTTGTCGGAAGCTAGTAGATACT**TGA**

>DfCCoAOMT4 (DfaA07G002980)
GTATTTCTAAATCAAACACTAAAAACATATGTATTTCTAAACATTCGTGTGTATTTCTAA
ATCAAATCCTAAATCTAAACATTCATCAATCTCGCTCAAACCAAACCCTAAAAACATGT
GTATCTCTAAATCAAACCCTAAATCTAAAACATTCATCAATCTCGCTCAAATCAAACCTT
AAAAACATATGTATTTCTAAATCAAACCCTAAAAACATATGTATTTCTAAAAATCAAACC
CTAAAAACGAATCTATACACATTACTACTTTTACATTACGCATATCTATTGAAAATCCAA
CCTAAAACCAAGCCAAATTTAACTAATTTCATCTAGGGTTTCAAATCTTCCCACACGAA
ATGTACCTCAAATAATCCATATTTATTGCAAAAAATCAAGGGAAATGAGGGGGTTCACC
TTGCTTACTCGATTTGGAAGTAAAATCGCGAGAAAAACGATGGATTTGAGTAGATTTTG
AGGGGGAAGAGAGTGGCGGCGGCGGCGGTTGCTTCGGCTCGGACTGAACAGAGTGA
GGGGGTGGGGGGCTGCGCTCGGGCTTAAGTCCCAGAGCACGGCGCCACGGCCGTTAG
CGCCGTTGGTACACAGCACGACGCCATGAGCTTTGGCGCCGAGCTCTGCCATCGTGGA
TCCTACGTGGCCAAGCACGGCGCCAAAGCATATGACGCCGTTCTGTGTACCTTGAGCG
CCATATACTTTGGCGCCGTGGTAAGGGTCCATTTTTGCAAATCTTTTCTCCAGGGATCCA
TATGTAAAAAACTTTCAAAATTTGGGCTAACAAGTAAAAATTTTACCATATTATTCTTCT
CGAGAGCCTAGCGTGTCCACATCTCATTGACATGGGGGCCCCGCAGACGCCAAGCTAA
TCTGCGTTCCCGTTTCTACTTGGGGAAAATTGCTCTCATACCACAAAGTTAAATACCAT
CCTCCCTATTGCCACATACTTAAGTGGCTTTGTTATTATGCCACAAACTTGAGTGTTTTT
CCTTTCTTTTGCCATTTCCTCCTCTTAAAGTCTATTTTAATTTTATTTTGCATTTCCCTAAC
GTGAATTGACCTTTTTGCCCTCGCTCCCCTACCTCGCCCTCAACGCTAACCTCCTTCCC
CGCCGGCTGCCAACTCCCCCCCCCCCCGCCTCCCCTCCATCCACGCCACCGTCTCCCTC
TTCAGCTCCGCCTCTGGGGTCCCCCTCGCCTCCGTCGACGGCTCCGCGCTCACCCTCCT
CCGCACCGCCGCCGTCTCCGCGCTCGCCGCATCCCCCATCTCCCCCGACCGGCCCCCC
TGCACCCTCCCCCACCCGCATCGCCGGCGTGAAGGACCCGACCAGGTCCAGGTGCGC
CCCCGGCCGCAGCTGTTCCCCCGCACGATCGGCTCCTACGACCCCATCGCGCAGCTCA
CGACGTCCGCCGCGGCCACCGCCTTGTCCATGCCGTCGACCTCCTCGACGGTGATCCT
CGGGTGCGCATCCCGAAGCTTCGAGGCGAGCACGGTGGATTTGGCCCTGGTGCGGTTC
CAGACGAGGATGCGGGAGATGGAGGAGAGGGCGGCCAGGTGGGCTTCGGCGAGGTA
GGGCGCGAGGGCGCCAGCGGGACGACGAGGCGCTCCGGTGGGGGAGGGTGCAGGGG
GGCCGGTCGGGGGAGATGAGGGATGCGGTGAGCGTGGAGACGGCGGCGGTGCGGAG
GAGGGTGAGCGCGGAGCCGTCGACGGAGGCGAGGGGGACCCCAGAGGCGGAGCTGA
AGAGGGAGACGGCGGCGTGGATGGAGGGGAGGCGGGGGGAGTTGGCGGCCGGCGGG
GAAGGAGGCTAGCGTTGAGGGCGAGGTAGGGGAGCGAGGGCAAAAAGGTCAATTCA
ATGCAAAATAAAATTAAAATAGACTTTAAGACGAGGAAATGGCAAAAGAAAGGAAAA
AACACTCAAATTTGTGGCATAATAACAAAACCACTTAAGTATGTGGCAAAAGGGAGGA
TGGTATTTAAGTTTGTGGT**ATG**AGAGCAATTTTCCCTTCTACTTGGCGGCTGCCTCCCG
CGACGATGCCTCGCTTCGCCGTCGCCTCCTCCTCCCTTCCTCCGGCGGCGGCGGCGGC

GGCTGCTCTCGGGTTCGGCGCCACGCCTCCTTGCGTCCTCCAAGCCCAAACCCTAGCC
GCCGCCGCTCCGCCCCCGCCATGGCGCCGCCGCCTGTGCATGTGCTCCTCCTCCTCGA
CAGCGGCGGCGGTGGAGGAGGCGCGGCGCGGGCGGAAGCAGCTGGGAATGACACCG
CAGCTCTACGACTACCTCCTCGCCAATGTCCGCGAGCACCCCGTACGCCACTCCCCCTC
TCTTTGTGTATATCTCTATCGTTTTGAACCAGTTGCTGCAATTTGGGGGGAATTTATTTTG
CTAGGTCCTGACCCCTGACCAATGTGCGTGTGTTGTTTATCCTACTCTGGATACGCTTTT
TAGATCCTTCGGGAGCTTCGCGAAGAGACGGCGGCCATGAGAGGAAGCCAGATGCAG
GTACATTAGCATACATAAACTTAGAATACTACGCTTTAGAATTTAGATGGAGAGTGTAAC
GCCCCATTTCTGAGATCGTTGGATTTCCCATTGCTGAGACACGGCCCCATTTTTGAGGT
CGTTGGATTTCCCCGTAGCTGAGTGTGCATCAAATGTGCTCATGCTTTATTCATAAAAGC
CACACTTAGTACAGTAGATTATAGCATGTCAGCCAAGTTTACAATAAAAAGGGCGTCCA
ACCTCAAAATTTATATTTCATCACAAAAGAACTTCTAAAAGATCCACAAGACGTAAAAC
CAGTAATATCTCCTAGGGAATCATCAAAGTCTTCTTCGAAAGGCTCTTCATTGACTTTCG
GAGTTTCTTTTGTTGATATTTTAGCAAGTATGAGTACGACCGGCGCCTCAGCGGGGTGG
CACGACTTCGCCGTCCGCCTCGTATGCATTGCCTCGACGGGACAGCATGCCTTTGCCAG
GAGACTTCGCCACCCGGCTTGGAGGCATCGCTTCGGCGGGGCCGCATGACTTCGTCGC
CCTCCTTGTAGTCATCGCCTCGGCGGGATGGCCTGGCTTCGGCATAAGACTTCGCCGCC
TGCCTTGGAGGCATCGTCTCGACGGGGCGGCACGATGTCGACCACCTCGTACGCATCA
CCTCGGAGGGACGTACCGGTTTTGGCGTGAGACTTCGCCGACCTCGTAGACGTTGCGT
CGGAGGGACGAAGCCACCCCTGCTCTGCTTCCATAATTTCTTGGTGGACTCTGCAGTC
GCATTATGATCAGTAAAAGAAAAACCCTCTTCCAGGGGCTGCAAGCTATCAGTTGCATC
GTCTCACCAGTCAGCACCCTTGCGCTTACTATTGGGGGACGGAGGGCTGGGATAGTTT
GTCACGTCAGCTCGCCCATCAGGACCTCCGCGCTTGCTATTGAGAGAGTTCTGTTGTAG
AGGTGGTCCAACCCCCTGTGACTGTGCCTTGCAACTGGCTTAATATGTAATAGGCTCAA
TAGTATAAAGGAATATACCAATGTACTCCTACCTTATGTAATTATTAAGGGATAGGAAGA
ATTTCTCATCCTACTCCACTTATATATATAAACCCCCAAGAGGGGCATGAGGAGGGGAG
GTCCACTCATTTCCCACATACCATCTCTTTGTTGGCTCTCCTCTTCTAGGCTCCCTCTTTA
CTATTTGGTCTGTATCCATAACATCTAATACTCTTTTAGTGTCGTTTTCTTGTTTTTTTCCC
TAGAATAGTCTTAATTATTTCGTAGGTATATCCCAAAAAAGAAATCCAATTTCACATATTT
ATAATTGTTTTTTCTGAAAATATAATGATAGTCCCTGGATACATTACCAAAATTCTGTATA
ATGATAGTCACTTTCAGCTGGTTGTTTTGTTTTGGTAGGTTTCTCCCGCACAGGCTCAG
CTGTTAGCGATCCTTGCGCAAACTCTTGGAGCACAGCGATGTATTGAAGTCGGCATCTA
TACTGTATGTGAACCTAGCGTTTTGTAATTTAGATGTTCCTACCTTCCACTCAATCAGTA
CTCAGTAAACACTAAATAGTACTAAGTTATTGTTTCTTCTTGGAACCATAGCATGCTATA
TTGATGTTATGTTAGTTGTTAACATGTTGTCCTAGCACACCAAACATAGAATCCACACAT
ATGTGCGAGACAAATAAGAAGTATATATGAGTGGGTTAACAAATCATCTGTAACATCTC
TGGTATCTGTCATTTTCCCTGGAAGTGATAGCATAATAGGCACTGCATCTTTCCAAGGTA
TATACCCAGTAAGATTTTAAATAGTACTTTCAGAATGCTGGTACTCTCAGAATGCTGAAG
CCTGAATCTTGCATATGTTTTCCTTCCTTCAAACAATATTTTCTGTCAGGGACAATAATG
TAATGCATGATGTATTGTTTATGTCAACTTGTCAAGTGCTGGAATCATTAGCTACTTTTGT
ACAAAAGCAACAATCATTGCAAATTTGGTAATCTTATCTTCTCACTCCTTTAATCTTTTC
ACAGGGTTATTCATCATTAGCTGTAGCACTAGCACTTCCTGAATCAGGTCGTTTGGTTG
CTTGTGAAAGGGACGAAAGATGTCTAGAAGTTGCCAAGAAATATTATCAGCGCGCTGG
TGTTGCACACAAGGTGATTAATATATGTCTCTTCTTGCTTGATGAGGTCGGTTCATTATA

ATCAATGAACTACATAATTTTCTGTATTTGTAGTTTACTTCTAGATTCTTGGCTAGAAATT
TACAATCAATTGCATGTGATGTTTCAGTAGGTTGGAGAACTTGTTTTTGATACAAAGCT
AAAATTCTTCCAAGTCCCAAGTTCCCAACCATAAAGATCATGGAAAAAAATTGTTCA
GAAGCAGGGCAAAGCCTTCTTTTTAGTTTCACTAATTCCCCCTTTATGTATTATTTATTTT
ACATCATTGCTCTTGTATTTGTTTCACTTTTTACTGACTTATGTAGCACACTATAGTTTAT
GAAATTAGGAGTGCCGCCATTTAATGCATCACATTTATTTGTAGGGAAACTAATCTGTG
TAGGTTTAATCTATATTTATTTCCTCTTTAATGATTTGTGAGCACTTAAAACTGAAATGTT
GACCTTTTTTTGGAATGCCTAGAATGTATAGCATATTAGTATTCAAGGACCGATTTGTATT
CTTGCATATTAGATTTTCTATGCTGATCTGTCTTAAAATTGCTTAGGTTGATGTCAAGCAC
TCTTTAGCTGTAGATTCCCTAAAGTTGTTGCTCGATTGTGGTGAAGCTTTCAGGTATAGT
AATTTTGGTATGGTATAATCCATCATTATATTAGTTGACCTCTTGCATGCTCTAACTAATCT
TTTCCTTTTCTGCTTTTTTCTAGTTATGACTTTGCATTCGTTGATGCAGACAAGAGAATG
TATGAGGAATATTTTGAACTTCTACTTAAGCTAGTAAGATTCAACATGAAGTTTACCTTG
TGGGTTATTTATAACTCATTGTGGTTTACAACGCATGTTTTCACTCTCTGTTCCCCATAGT
GTTTTTTTCATAATATTATAGAAGATTCCCCATTCTTGCCATAAGTTTTATGATTTTATCTA
TACTTTCCGTACATAGTGATCGTAGTACTTATGCTTTTTATATGTTGAACATTATGTTTTTG
TTGTGTTGTAGGTAAGAGTTGGTGGTTTAATTGTAATGGACAATGTCCTTTGGTACGGA
AGGGTTGCTGATCCGCTAGTAAGTTCTGTTCTCTTTTTGAGTTTTGACATGATAAAGATG
TTACAGGGTAGACATTAGGTAACACTTTTCTCTGGCATTTTTTCCTTAGGTGAATGACGC
AAAGACAATCAGTATAAGGAACTTCAATAAGAAACTTTTGGAGGATAAACGTGTCAAT
ATCAGCATGGTCTCTTCCTCTCTCTCTACCTCTCATATGTTTGGTCTACCACATAGTTATT
AGTCATTACTTGCGAATGTTGATTGTGAAGCCTTAGAAAATATTTTATATGTCTGCATATT
TATTCTGTCGCCTAGAGCATGTGAAGAGCACCAACAATGATGTGTAGCGGCACTGCAAT
GCCAGCACAGTAAATATCCCAAATTCGAAATTTTTATTTGCCTAATAATGTGGCACATTC
AAGCCATGGTCATGTATACCTGTTTGGATGGGGGGTATGGATTCGAATCCTTAGGATTTA
GTTCAAATTTGCACTAAATCCCAAAAATTCACAAGGAAATCCCCCTCATCCAAACACCC
CCTAAATGTGTTGTCCTCTTCACTATCTTTATAGTTGTTCAACTGCTTCAGAACCTTTATA
ACCCCCTGTATCCAAACCTTCTTTTGATACACCATCGTTTGGTTTTAGTACAACTTCTGT
GAGAAGAAAACGGTATGTGTAACATTGCTCCAATTACAAGTGTATTCTTGTAACCGTAT
GCAATGAGAACCATAATATTGGTACCATTTAGATTTTCATTTGACATGCTTATATCATGAT
TCAGTGCATCATATCCCATGCCCTTCTCATGTGTTGTCTGTTCATACCACCTGTCTGTGC
TTTAGTACTGGATCTTAATTAACAATGGTATTATATTTTTGAGAAATTGCAGGTGCCAAT
TGGGGACGGGATGACAATTTGTCGGAAGCTAGTAGATACTCCCCTTCCGAGGTCAAGC
TCTCAA**TGA**

>DfCCoAOMT5 (DfaA07G014340)
GCTCTAGCTCATCTCGACATGAGGCTTATATTTACGAATGCGTTTTGCAAATAGTCGAAA
CTCATATGTTGAATTCTAGTAACATGGGACCATGCATTGCAAATGTGTTTTCTAAGTGCT
TGAAGTCCATGCACCTACTCCTTGTGGCATGATCTAAGTTTGCAAATGTGTTTGTAAAA
GTTGGAGATCATGCATTATCCCTTGGCATGGTCTAAGATTGTTTATTGCTATTTCCTTAGA
CTTGGACCATGGTAGTGACTCCTTGGATGTTCTTTGATTTCTGCATTGATCTACTGTATTT
GAGGTTATGCAAATAGATGGGTTGTGTAGCATCAGGCACTTCGCCGCGTCGGACATCTT
CGCAGCATCATGTCTGCACTCGTCTCCGCGTCAACTCTGCATCGACTCCAGGCCGCCA
GGCCGTGCCGCGCCGAAGTCTTCATCGAGCGATGTCGTCGCGCGTTGGCCGAAGACTC

GATGTCTCCGCGCGCTGGCCGAAGACTCATGTCCCGCTCGGGGTCCGTGCCGACGCGA
AGTCCCCGGTCGCCGAAGCCCCCCGCTCGGAGTCCACGCAGGCACGTCTCCGAAGTT
TATAATCCAGGCATGCAGCACCTTCGCGTATGCATGCAGGGAAGCATTCGTCCACCCAT
GCATGCAAATAACTATTCACGTGTGATCATCCATCCGTCCATGTATGCATTGTATTTGTCA
TATACTGCATGCAGGAAAATACTCCAGGCGCCTCTACGTATCCCTCGTACACGTGAGTT
CGTGGTCCAGCTCTCTGCGAGGAAGCGCGCGGATGGGTGGTGGCCCCTCTCCGGATGC
ATCATTTAGTGTCGACCAAGCCATTAACATATTTGTCTTCTAGCGCTTGGAAGTCCACTC
TTGAAAGTCTTTTAGTTGTTAAGAGTAGGCCGAGATACTTCAAGGGGAAGGACACAAG
CTGCACAGGTAGGTCCTGCAGAACCTCTTGTAGGTCAATGTGGTCGCAGTTGATTGGC
ACCACCTAGCTCTTGTAAGGTTGGTGTGAAGGCCTGTGGTATCCCTGAATTTTTGTAGA
ATTATGACCAAGGTGCAGACGTCGTCTTTCTTTAGTGCTAGAAAGATGGCAGCATTGTC
CGCATAAAGGGAGGCGCGAAGGGTCGCCGCACGGCCCCCTAGAGGTCGTAAGCCCCC
AAGTTGGTTTGCAATATCAAATATTTTCTTCAGTGGATCTATAACTAGGATAAAAAGGAA
AGGGGATAGGGATCCCCTATCGCAAGCCTCTGCGGTGCTGAATATATTCACTAGGGATC
CCGTTTAGGAGGACTGTAGAGGTAGCGGTGGAGAGAAGGCAGGAGATCCAGTCTCTT
TATCACAGTGGAAATCTCTTGTTCTGTAACAGGTCGAGCAGGTATTCCTAGCGGACCGA
GTCAAATGCTTTCGTAATATCAAGCTTGAGAAGCAGGGACAGGGTTATGTTTCGATGGT
GTCTTTTAGCTAAGTTGCGGATATAGAGTTAGTTGTCTTGGATTCATCTGTCTATGATGA
AGGCGCTCTGACTTGAGGAAATAAGATCGTCAATGAAGGGTGTTAGTCTTAGAACCAA
GATTTTTGCTATAATTTTGGATACGTTGTGTATAAGACTAATGGGCTTGTAGTCTCTTGGC
AACCGTCCCTTTTTTCGGTGGGCCATTTTTCTAGTCAATCCACGACAAATTGGCTGGGT
AGATTGCGGCTGATGTGGTGGTAGGAATGTGATGCGCATGGACTACCACGACGGAAGG
ATGGGCACGCAAAGACTAACGAAAAACGCCTTGCGTTACACATGCATTGATCTAATTG
GTCGGTCAACAATCAAAGCAAGTGATTTATTTCTTTTCAAAAAGGAAAAGAAAAGCAG
CACCTAAAAGCAAGTGATTTTCCATCACTTGATAAATTAATACAACACGCCTAAGCTGT
GGGAGAGAGCTCAGGTAGGCAGCGCGAACGAGAGAGACACAGAGAGAGATCGAGCC
ATGGCGAACGGCGGCGACAGCGTGCCCAACGTCCACAGCGACAGCGACAACGACAG
CAACAGCCACACGCTGCTCAAGAGCGACGCCCTGTACAAGGTAACACGCTAAACACG
CAGATAGTACGTACGACGAGCAAGTAGTGAACGTTCGTTCGTTCTTTCTTGAGAAGGA
CTAGGGGACGCTACCGTCCAGCCCTTCGCCGCCGTCCGTCGCCAATCGTCCTTCGCTGT
CACCCACCACTAGACCACCGCCAGACCGCCTCCTAGGTCTCCCTCTACGTTCAGGGGC
ATAGTGAACCAGTTGGCGACGGTGGAGGAAGAGGCGTCGGCTGTGAGTCGTCGTCGT
AGCCGAAGCAAGAGGAGCAGGCGTCGGCAAAATTTCTTTGTATGAGAGGAAAAGAGA
TGATAAGCCGTCGGGATGGAATACGATGGTGTACCGTTTTCTAGCATCTCTCTTCTTTCT
TTGCTGATGGTGTCATGGTCGTTGCCGTTGCCGTGCAGTATGTTCTGGACACGACGGTG
CTGCCGCGCGAGCCGGAGTGCATGCGCGACCTGCGCCTCCTCACGGACAAGCACAAG
TGGTACGTGGTTTACTTACTAGCCTCTCGCGTGTTTTTTTGTCGTTAATTCGATGCTGTT
CGGTTGGGTGTGATGAAATCATGGCATGGTGATCTCAGGGGGTTCATGGCGTCGTCAC
CGGACGAGGCGCAGCTGCTGGGGATGCTGATCAAGATGGCCGGCGCCAAGAACACCA
TCGAGGTGGGCGTGTTCACGGGCTACTCGCTCCTGGCCACCGCGCTCGCGCTCCCCGA
CGACGGCAAGGTGGTGGCCATCGATTACGACCGTGAGAGCTACGAGGCGGGGCGGCC
GTTCATCGAGAAGGCCGGCGTGGCGCACAAGGTGGACTTCCGCGAGGGCGCAGGGCT
CGAGAGGCTCGAGGAGCTCCTCGCCGAGGAGGCCGCGGCGGGGCACGAGGCGCGGT
TCGACTTCGCGTTCGTGGACGCCGACAAGCCCAACTACGTGCGGTACCACGAGCAGCT

GCTGCGGCTGGTGCGCGTGGGCGGCACCATCGTGTACGACAACACGCTCTGGGGCGG
CACGGTGGCGCTGCCGCCCGACGCGCCCATGTCGGACCTGGACCGCCGGATCTCGGTC
GCCATCCGGGACCTCAACGCAAAGCTCGCCGCCGACCCGCGCATCGAGGTTTGCCAG
CTCACCATCGCCGACGGCGTCACCATCTGCCGCCGCCTCGTG**TGA**

>DfCCoAOMT6 (DfaA07G014350)
TGGATGGATGAGGATTACCATTTTCAATGGGCTTGGGGCACTTGGAAAGAGAATGTCC
AGATTCACCACAGTTACAGCAGAATTTCTTCAAATTCCCATCACTTTTCTCAGGGCAAT
TCTTCAGACTATGCCCGCCCGCTCGCTGCAGAGCAAGCAGATCTGAAAAAGAACATAA
CATTATTACATAGTTAAAAGATAGTGAAATTGAACGCTTGCAAATAGAGGAATCGGACA
TAAAACCAGCTGATGAAAAAGGACATGACCAAACACCATTTACTCTCTGAAACATTTG
CCATTTTACTCTCTGAACATTTGTTCATCAATACAATCCATTCATACGACAGCCAGGAGA
TGCCAGATCATATACGCACTGATCAAAGTGGCACATGTTTTCCAAAAGTTACGTTTGTG
AGTTTGCATGGTAGTCATAGCTCATTGCTCTCACGGAACACACACACACACACACACA
TAGTCCTTGCCTAATGAAAATCTCACCCAGCAACGTCAAACTAAAGGAAAATCATCATT
GCTTCTGCCGTTTTCTACGGTTCTAATACCCCCAACAATTAGTACCAAAGCTGCTCTAA
ACGTAATCCCCAGGCCAGCTTATAAAAGACAGCATTTTCAGCTAAGTGATGGTGGGCT
GGTACATTGTTCTTGTTCCAGAGGGCCTTCTTGGGGCAGGCCTTGGCGACGTGGCCGG
CGGCCTTGCAGATGAAGCATCGCTCCCCGGGCCACATCCCGGGAACCCGCAGCGGGT
GCTTCGACCTCCCCGCGCCGCCCGCCTCCTTGAACATGCTCTTCTTGTTCTTCTTCTTG
GTGCCGTCGGCGGGAGGGGCGGGTTTGGGTGGGAAGAGGTCGGGGTTCGCCTCGCGG
AAGCGCTTCCGCAACAGCCGCTGACGCTGGCTCACCATCGATTCAAGATTGCTACGGC
CGCCTTTCTCTCTCGCGCGCACGTGTTGAGACTTGAGAGACGGGGCTGTAGTGCCGGC
CAAGACGGCAGCGCCATTGGGGATCGGGAGCACGGCCGGGGGCCAGGCCAGGGGGA
CGACGACACGGGGAGGACGGGGCGCGGCAGCGCGGTACACGGGGCTGCGGTGGCGG
CCAAGACGTCGGCGGTCGGGGGGATCCCGATTTGGGAGGACGGGCTTAATAGCGTGA
GAGGAGGATTAGGGATTCGCGATTGGTGATCGACTGGCGTTCACGATCGGGTATTCCG
GTATAACCTATACGATTGGGCCTTCGTAGTCCCGCGGCGATGCACGAGCCAAACCAGCC
GCGCGAGTGGCTCCAAGCTCGGCTCGATACGGGCTCGATTTGGCTCGAAAAGTCACGA
GCTCGTTAGAGCCAAGTAGACTCGTAAGTCTCCAACTATTTTTTCAGTCTTACTTTACAT
TATTGAAATCTCCATGTGTAAAATATGCCCACAGACCTGCACTCGTCGGATCGGAACAA
AGGCAACGTGCCAACGTCTAATCCCGACCAGATGGTCCACAGGCCAAACTGCATGCGC
AAGCTCTCTCCCCGCAAAAGATCACAGAAAGCATTCCAAACAGCAATCACTCCTAATT
TTTGAAAACCAAAAGCTATTTAAAATGTGTTAGATGACTTTTTTTGGCCTGTAGCTTGT
GGCTTAAATTATTGAATTCTGGTTGTTCGTGAGAGGTAGTCCATAGAGAAAAATAATCA
CATACGATTTTAGCTGGTACAGCTGAACTCAGTATACCTTTTATTACAATCTTCCTGTTG
ACCCCGTTTTGAAAAAAATACATTGTTTGAAAGCAAGTCACTATAACGTAAGTCATATG
CCGCAAGTTATAAATGAAAAAGAAGTCACATGTTATCTTTTTAACATGTTTTTTTAATTG
TTTATAGGGACAGAATGATATTTTTATTTTTTTTCCTATTTTTTTAATGGAAAGGTCGAGA
GAAGGCCGAGTGGCGCAGCCCGCATCTCCAAGCATAAAGACGCGGGGGGGGGGGGG
GGGGGGGGGTTGTTAACTCGACAAAGGCATCGTGCGGCCGCAAAGGAACGAAGCAC
TGAGGTAATTTTGGCAGTTTGCGGAACGAAGCGCTGAGCTAGGCTGGCAGCCACTTGC
CGTTGTACTCGCGCTTTATGCTCTGCGTTTGTTACACTTGTGATATGATCTGTCTTGTTAT
CTAGAGCAGTGAGTGCTTCGGTTCTCCTGCGCGAATCGCTCACCGGCCTAGCACGGAC

ATGGCTGCGAACGGCGACGCCGGCGGCGAGGTCAAGGACATCCACACCAACGACAG
CACCAAGACCCTGCTCAAGAGTGACGCCCTCCACGAGGTACGCAGAATCATCCATCCC
TGAATCGCAGTTCGGCGCGTGGATCGATCTGCAACGCCTTTTCTTTTTCTTTTCTCTGTT
GCAGTACATGTTGAACACGATGGTGTACCCGCGGGAGCACGAGTGCCTGCGCGAGCTC
CGGCTCATCACCAAGGAGCACACCTAGTACGCGTGGCTAATTAATTCGTTTCTCTTTCT
TTAATCTCGCGTGATTAGTTTAATGTTTCCATGGATCATGGTGGATTGATCTGGGGTTGT
TTTTTTTTTGTTAGTGGGTTCATGTCGTCGCCGCCGGACGAGGCTCTTCTGCTGTCGCT
GCTGCTCAAGGTGATGGGCGCCAGGAACACCATCGAGGTCGGCGTCTTCACCGGCTG
CTCCGTCCTCGCCACCGCGCTCGCCATCCCCGACGACGGCAAGGTGGTGGCGATCGAC
GTCAGCCGGGAGTACTTCGACCTCGGCCTGCCCGTCATCGAGAAGGCCGGCGTGGCG
CACAAGGTCGACTTCCGCGAGGGCCCCGCGCGGCCCATCCTCGACGAGCTGCTCGCC
GACGTACTGAACTAACCGCCGCCAAGTCATTCTTGATGCAATGCCGCCGGCCCGTGGC
TTCAACGGTTGCCATAATTACTTGCTTATCGATGTGATCTTGGTGGGTGCATGCAGGAA
GGAAACGTGGGGAAGTTCGACTTCGCGTTCGTGGACGCCGACAAGGGGAACTACGTC
AACTATCACGAGCAGCTGCTGCGGTTGGTGCGCGTCGGCGGCGTGCTCGCCTACGACA
ACACGCTCTGGGGCGGCAGCGTGGCGCTGCCGGACGACACGCCGCTCACGGAGGACG
ATCGGGAGATCCGGGGTTCATTCAGGGCGTTCAACGCCAAGATCGCCGCCGACCAGCG
CGTCGAGGCCGTGCAGCTTCCGGTCGCCGACGGCATCACTCTGTGCCGCCGTGTCGCC
TGATCCGTGCTTGTCTGGCGCGCGGCGCGATTGCGAAGTGTGTATGAAATATGAATAA
ACAATTGTGTGATTGTATCAACATCCAGGTTTGCCTCGCCAGTTGTGGAGTTATTGATGT
TATCTATTCATCGAAGAACATGTTGGTCTATGTAATGTCATCTGGTAGCTGCTTGCTTTGT
TGATTTGATCTATCATGATTCAGCTTCTCTGGTCTAAAATATGATGTGAATTGTGATGGAT
CTTTTTCTTTATTGTAACATAAATATGGTAATGAAAGATGTCAGAATCA

>DfCCoAOMT7 (DfaA07G014530)
ATTAAACCAAATTTTTCCAGATATTTGAATTCGCATTCAAATAGAATTAACAAAGATATG
ATAGTATGATTCAGCAAAACAAAAGTTAGATTTAATGTGCAAGGAACAACACGGCTGC
GCATCACAAATAAAATGTTTCCACATGAATACGCATGTGAGTGCGCACGTGCATGTGTG
TGATTTTAGCAATTGTACTCATGCCACCACATTATATGTGTCACATATTTAAAGGGTAGC
AAAAACTCTAGTTTTATATTACACTCCTACTTGTACAAGCAATGCGGGACTAAGTCATAC
TATAGAATTTTTAGGAATAAATTGGCATAACACTCGTGGGTCCAATACTAATAAGTGGTC
GGCGAGTTCACTGGTCACTCGCCACTGCCCTCACCCTCCCCGATGATAGCAAGGTCATT
GCGATCGACGTTAGCTAGGGGTACTTCTTGTTACAAATGTGGGTAATATTTTAATTCCAA
GTTTTATGAGGAGACAAATACAATAAACTAGTGATGCAAAACTTACTACTAACAGCAGG
CTACTCATCATACTTCAGAATATAGTCAAGCAAAGTACAGTAAGAAAGTCATACGTGTG
CGCGAACGACTTACTTGTTAGCCTGAAGAAGAACAGGAAGCCGCGCAGGCTTGACGC
AGTACAGATCGTAGATGTCGCAGTGCAGTGAGCGGTCGCGCAGATGCGCTACGCAGAA
ACCTTATTGCCACCCCGTGCAGGTACTCTCTGCGGCAACGGTTCGGAGATAGCCGCTCT
CCCTCCTTCCAGGTGCATGCCGGGAAGAAAGGTCGACGAGGTTCAGACAGTACAGTA
CTTGAGCACAAAATCACAGGGAAGATGAAAATGGAGGCAGTGAGATTCTCTCTTTTCT
GGACGTCTCCAGGCATATATAGACAGACTGATAACTGGCATGATTAAAACAGCCATTTA
CCCACTGGTAACTGGCACATTCAACCAGCCATTTATGAACTAGTAACTGTCTATCATCA
AAACCCCAGTAAAGCCTCAAATTCAAACGTTTGAATCTCATTCAAACAAAGAGGAGGT
GCAGGGTTCAACAAAAATAAAGATGGACTTGATCTGCCAAGTGGCGCGCCACGTCAC

GGCACAGTCGCGCGTCGCTAGTCACAAGTCACGCGATTTCATGCGAATATGCGTGCGC
ATGCGTGCGCGCGTGTGTGATTAGACAGAACCTTACGCATACCATCTCTATCCATATTCA
AGGAGTGGTAAGGATTTCACCTTATAAATCGGTCTTCCTCACCTTTCCTTAACAATATGG
TACTAACCATGCATGCTTTGGAATTAAGGAAATGAGCCATAAATAAATGGGCCAATTTC
TAACACTTCCACCCAGGCCTACCTGTCATACAAAAGTGTTGGGATACGATAGGTTATAC
TAGTGCAAATTAAAATTTCTACCGCGTAAACTAGAAAGCCATATGAGTAAAAGATAATA
GATCATTACCACTAGACGCGTAGTGTAGCGGAAGAATAGTCGAAACAGCCTAGTTCAA
CGTAGCAGGTATCAATATAGTGGGAGTAGTCGATTATCCAACGTTTGTGAAAAGCGATA
TTTGGAAGCCAATTGATCTCCATGTGCCAATTGTCTTTTCGTCGAATCATCCCGATTAGT
GTTGCAGTTGCATCAGCGCTTTACCGAAGTCCACACATCTAGGGATGGAACATTGTGC
GCTGGTGTGCTAGCACTAGCGCACACAGTAAGGTCTTTCGGAAGGAGATGGCTGGGGT
TTAGTAGGTGGTGCAATGTCTCTCACTGTCTCACACATAAACCCATAGCCACAATACTC
CCCTTATGTAGAGCCTCCAGCCGAATCATAATCCAACATGAGTAGAGATTATATCCTACT
TGGAGAGCGTTCAACGAGATCCTTATCCAATACGAATAGATATTGTATCCCATTTGAAAA
GTCTCCAACTAGATCCTTTACTGTTCAAATCCGTTAAGTGTGTGACCTTATAAATTC**ATG**
TATATTTTGTTGTACCCAGAAAACATCTTTCCTATCACTAGTCGGTAGCGGTCCATAGCA
GAACATATTGACTCTAAATATACACAAAGATCAATCTGGATGAACCTTATGCAATATGAT
CTGCAACTAAGTCTTGGTCTCTCACAATTAGTTCGTAACTCTTTATGACCAAATCGTATC
GCTTACGACTAGTTTATATCCCCATACGACCAGTTCGCTGACTATTTTATGATATGTTCGA
CTAGCTCACAACCAGTTAGTATATCCATACGATCAATTCGTGTCAGATCAGACTTAACG
ACCTGTTCGCATATTTTCCTTTTACCTCACGATATCTGATCGAACTTCAGATGAGAAACA
TATCATTCATGTACTATTCAATCTTTTTCCGATCTAGTGATAGATAACTTCATGCAGTCGA
CTCTTCAATCACTCGTGGTATGACCATGCATATCCTTTATCTAACCACATCGAGGGGTCC
AGAGATATCTCTCCTATTATGTAGGAGGGGTAAATTCCATATTCGATCAGTCATATTTCTC
CGCATGTTTTATAGCATACCCGAAAGATATCTTTATAACTACCCAGTTATAGAGTAACGT
TTGCTTGAGACCAAATGATGGCATATAAAAATACAATACCATGTTCTCTAACATATGTCT
ATATTATTAATGTGATATTCTATATCTATGATTGGTGAAAGGTGGTCATCAATTAATATATG
TGCTGGTCTATAGGTCACTAATGTCCTGATTAGTGACATCTGACTAAAGATCTTTTAGAA
TAACACCATGATAAAGAGCTTCATTAATAAGTCATATACTTGCTAATATCTATAAATGATA
ATTTGTTCAAAGAACAAAATAATAATTATTAAAATAATCATAAATATAGTAATAGTATAAT
CAATTTTATGATCGACTCTAGAACATGTCGCTAACAAAAGGCTGGCATCGCTCACAAAG
CCGACTTTCTGCGAGGGAGCTGGGCTCGCACGACTCGAAGGGCTCCTCGCCGAGGAG
GCTGCGGCGGGGCAGAAGCTCGGTTCGTGGACGCGGACAAGCCCAACTACGTGCGGA
ACCACGAGCAGCTTATGAGACAACACGCTGTGGGACGGCACGGTGGCGTTGCCGCCC
GACGTGCCCATGTCGGATCGCGACAGGCGGTGCTCGGTCGTGACCAACAAGCGCGTC
GAGGTGTGCCTGCTCGTCATTGCCAATGGCGTCACCATCTGCCGCCGCCTTGTGTGACC
GACACGAACAGTGGCCACGACTCAACGTTTCGGTGGTTTCAGTAAGATTTCACGAGTT
GAGGGACTGAGGGTGAATGTAAGAGTGATCGATCACCGTGTTTGGGATCGACTCCTTT
AAAAGTTAAAATAGCTTCACGATCATCAGCTTATGAGATGGAAAAACTGTGTCAAGAA
AAACGTATGGTTGCCAATGCAAAATGGGTGCAAGCATGCAAACGTATGGTTGGCTCGT
GCGCCCAGCCTATCAGGCCGAGGCGGCGTACACACAGGCCGAGTGAAATGAATTCCTC
CCCAAAAAAAGTGCAAGTCGAGAGAATCAAAAGGCCTGCAATGGCAGCCGATTCTCG
GAGTATCAGGTAACATGCATAGTGCATTGAAGGCTTTCCTTACATTTTAGTTAGCTTTTA
GCTTCTTGCCCACACGAAGAACAAGAGTGGGACTCATGATTTTTCCTTCCGGGGGA

AACTACATGAAACTTCCCAAAAGTAGGCACTGAAATTTTTGAATTTTGTCTGTTTATGG
TACAAATTACGGAGAGAGAGATTTTTGAATTTTGGCTGTTTACGGTACAAATTTTTGAA
ATTTTTGAAATTTGGATGATACAGATGTTAGACTTTTAACGTGTACATCGTTTAACTTGT
AGACCCCGTTTTAAGAAAATGCTCCGTTTGAAAGTTAGGTACTATAGCAAAAGTCAAA
AGCCGTCAGCTTCGGAGGAAAAAAAACAAGCCTGTAGTATCTAGTGCACATCTATGTT
AAGACATGGTATCATGCCCTGATACAAGTAGTTATGACGGCAACATAAGTTCCTATTGTA
GGCTGGATCCGGTTGTAATACCGTACAGCCCAAGACTCGCTCAAACCCAGCTCCCAAG
TGGAATGGGCCTACGCATGTGCAGCAATCGTTTCCATTTTTCTCATCGTTTTGCTTAAAA
GGCTTAAGCTATTGTTTTGGACGAATAAACATTATATGATCATTTAACTCATTTCAAATTG
GTAGTGTGGTGGTTCTAAGGGACATGATATTACACCCGTGTTATGAAACACGATCTGAG
TGCACATGATGATGTGAAGAACAAAAAAGTGCCAAATAATAGCAACTAATTAAGCAAC
CACAGCAAAAAGGAAAGAGTTAGCAAAGCAATTAGGTAAGCACTACTTCGAAGGGTC
ATAGGTTCCCGTCCCTACTTTTTTTAAAAGAAAAAAAACTATCCTTCCCGTTCATATTTT
CTGCTGATGTCCACAGCAAGTTCAACAGCAGTAACAAGACGCTGCTCAAGAGCGAAG
CCCTGTACCAGGTAATACACTAAGTAACACGACAGTGACACACCTAGCTATGTCAAGA
ACATTCGACGAGTCAGTGATCGTTCGTTCCTCTCTTTTTCTGCTGATGATGATATGGCCA
TTTGCCGTCGACGTGCAGTATATTCTGGACACGATGGTGCTGCCGCGCGAGACGGAGT
GCATGCGCGAGCTGCGCCTCCTGACGGACAAGCACGAGCGGTACGTACTGCGCGCGC
TATACATCTCACTCGTTTTTAGTAGCAGTCGCTTTTCACTTGCATTGCCAATTTGCCCAG
ACATGCATGATGGATGATGCGTGTTTTTTTTTGCTTAATTAATTTTGCTGTTGTTTGGTCT
GTGTGATGAAATACATATTGGCATGTGTAGGTGTTCCATGGTGTCGTCCCCGGACGAGG
CCCGGCTGCTAGGGATGCTGATCAAGATGACCGGCGCCAAGAACACCATCGAGATCGT
AGTGTTCACGGGTCACTCTGTCCTCGCCACCGCGCTCGCGCTCCCCGACGACGGCAAG
GTGGTGGCGATCGACGTCAGCCGGGAGTACTTCGACCTCGGTCTGCCCGTCATCCAGA
AGGCCGGCGTGGCGCACAAGGTGGACTTTCGCGAGGGCGCCGCGCTCGCGAGGCTCG
ACGAGCTCCTCGCCGAGGAGGGGAACGAGGCGCGCTTCGACTTCGCTTTCGTGGACG
CGGACAAGCCCAACCACGTGCGGTACCACGAGCAACTGTTGCGGCTGGTGCGCGTGG
GCGGCACCATCATGTACGACAACACGCTGTGGGGCGGCACGGTGGTGCTGCCGCCCG
ACGCGCCCATGTCAGACCTGGACCGGCAGCGCTCAGCCGAAATCCGGGATCTCAATGC
AAAGCTCGCTGCCGACAAGCGCGTCGAGGTGTGCATGCTCGCCATCGCCGATGGCGTC
ACCATCTGCCGCCGCCTCGTG**TGA**

>DfCCoAOMT8 (DfaA07G014550)
TGATCGGTCAACAATTAAAGCATGTGATTTATTTTTAAAAGAATAATTAAATACTAAAGC
AGTGGTTTAACACACAGGGAGAGTAATGGAAAGAGCGATCGAGGGATTAACCAGCAA
GTCTCTTAGAGTCGATAAATATCACGCCGAGCTTGTGGGGGTTGTTGGAGCTAGTGGG
GGTGGGTCCCGCACAACTGCACAAGCTAAAGCGGAAAGAGCAGCGAGCGATCTACGG
CGACCGGCGGCGACAGCGTGCCGAATGTCCACAGCAACATCGACAGCGGTAACAAGA
AGCTGCTCAAGAGTGAAGCCTGTACCAGGTAACACACTAATTAACACGACAGTCGACA
CATCTATGTCAAGAACGTACGAGTCAGTGATCGTTCGTTCGTTCTTTTTTGGTGGCCGT
CGACGTGCAGCGCTTGGCGGAGAGCATGGCACGAAGGTCATGTATAGACTGAACGAA
GTCCTCGGCGGAGACCATGCGAAGTCTCCACAGAGGAGTTAGAGGCAACATTCACATA
GAGCTTAGCGAACATCATACGAAGTCTTGTGAAGACCGGACGAAGTCTTCGAAGACC
AGGTCAAGTCCCCGCAGATAAGTCGGAGACGACATCCGCGCGGAGTTTGGCGAAGGC

CATGCGAAGTCTTGCAGAGCCCGGACGAAGTCCTCGGCGGAGATTGCGCGAAGTCTC
TGCGGAGGAGTCGGAGGCGACATCCACGTGGAGCTCGGCGAAGACCACACGAAGTCT
TGCGTAGATTGGGCATAGTCGTCAAAGACCGGGTGAAGTCCCAGCGGAGAAATCGGC
GAAGTCCTCGGCGGAGACTATGGAAGATCCGTGGAGGAGTCAGAGGTGACATCTGCA
CAAAGCTCAGTGAAGATCATGCGAAGTCTTACGGAGACCGGGCGAAGTCCCCGCAGA
GAAGTCGAAGGCGACATCCGGGCGGAGCTCGGCGAAGACCATGCAAAGTCTTGCAGA
GCCCGAGCGAAGTCATCGAAGACCGGGTGAAGTCCCCGCAGAGAAGTCGGCGAAGC
CCTTAGCGGAGATCATGCGAGTCGGAGGCGGATGTTCCGTCCCCAAGTGTCAACCACA
GAGAAGTCGGCGAAGCCCTTAGCGGAGATCATGCGAGTCGGAGGCGGATGTTCCGTC
CCCAAGTGTCAACCGCAGAGAAGTCGTGCCTATTGGCAAAGACCAGACCACAACGCG
GTCAGAGGTGTCACCAAGGTCATGACCGTGGCGTGGTCGTGGATGTTGACGCAGCCG
GCGGTGGGGCAAAGACTTTGGAGTGTGAGCTCCGTGCAGGATCCCCGCCGCCAGAGA
GGCTCGACATTCACTGGCGTTGCTCAGAGGCCGACTCCTTGGTGTGGAGGTGACGGCG
CGGGCATCGCGGTTGACGGTCGCACCGGCGTAGGCGTCGTGGTTGACGGTCGCTTCCG
CTGCATCTCTCTGTCGCTTGGGTGCCATCGCTTTCTAGCATAGTTTCTTGCGCGGTCCCC
ACGGATGGGTACAACAGTGGGTACAAGGTACCCTTCTTCATAGGAGATAGTTAAGAGA
TATAGGGTGAGAGAGAGCTGGTGGTAAAATGAGTCAATCTCCCCTCCTCATACTCCTCT
TAGGGGTTTATATACATGAGTGGAGTAGGATGTGAATTTTTCATCCTACCTCTTAATAATT
ACAAAAGGTAGATATATATTGGTATATTTCTCTACATTATTTGGCACATTACATATTAGAC
TAATAAGATCCAGCCACGGAGGGTTAGATCACCCCTACAACAGTAAGGATGAAAGTGT
AAACACCAATAAAATAGCTTAAGCGTTGATCAAACGCCACCTTTGATCGGTCAACAATT
AAAGCATGTGATTTATTTTTAAAAGAATAATTAAATACTAAAGCAGTGGTTTAACACAC
AGGAAGAGTAATGGAAAGAGCGATCGCGAGAATCAGCCCGCGGTGCCGCACAACTGC
ACAAGCTAAAGCTGAAAGAGCAGCGAGCGAGCC**ATG**GCGACCGGCGGCGACAGCGT
GCCGAATGTCCACAGCAACAGCGACAGCGGTAACAAGACGCTGCTCAAGAGCGAAGC
CCTGTTCCAGGTAACACACTAATTAACACGATAGTGGACACATGAAATCTATGTCAAGA
ACGTACGACGAGTCAGCGATCGTTCGTTCGTTCTTTTTTTGTAAGCGTCGACGTGCAGT
ATATCCTGGACACGACGGTGCTGCTGGGCGAGCTGCGCCTCCTCACGGACAAACACGA
GCGGTACGTACTGCGCGCTATATATCTCACGCTCTTTTTAGTAGCAGCCGCTTTGCATTT
GTATAGTTGCATTGACATCAATCATATGTCAGTATGTGGTCATTAGTCTTCTGTTGTTTGA
TTGATACGAGGCCCAGCTGCTGGGGATGCGGATCAAGATGACCGGCAATATTTGTGGG
AAATATTTTTAATTTTAAATACTAAAAGGTGGCATGTATTATAAAATACTAGTGATACGAA
ATTCATAACTAAAGGCAGCAGACGAATCATATTTGTACTTTTAATATACGCAAGAACAG
AATTATAATAGAGTCGGTCGGTCGGTCATACGAATAACGTACAATGGGTTGATAAAGAA
AATGGAAGAAGCACAAAGACGTGCGGTCGTGAAAGTAGTCGATGAAGAAGACGGGC
GGCCGCACAGGTTGCGCTGTCTAGAAACCTTATTGCCACCCTATGTAGGCACTCTCTGC
GGCAACAACTTGAAGATAGCCATTCTCTATCCTTTCCAGTGCACGCCAAGAAGAAAGA
CCGACGAGGTTTGATGACTCAACACCCTAGCACAGTTCACAAGAGGGAAAAGATAAT
GTGAGATAGGAATGGATGAATTCGATGTCACTAGCGTGGACATCTGTAGGTCTTTTATG
GATGTTTTTTTTAGATAATGGAAAATAATTTCCGGCCTCTGCAAGCGCACACAGCCAAG
TTCTTACAAGGTGTTCAGCTAATTGCTGTTAAAAAAAAGGAAACGAGAATACAAGTTG
ATCAAAGTGATTAAAAGCAAAGTCTATTATTGCTCCTCCATCCGCTCTTGGCGAAAAAG
TCCAATGTGACAACTTCCAGTGCTCAGCTCGCCAGTCGAATGGTCTCCTTTGCATCCTC
ACGCTGCAGCAATGCCCAGAACCGCAGCCAGTATGTTCCCCTGAAGGTAGTCTGCATA

AAAGAAATAAATCGTTTCTTTTCAAAAATAACATCATTCCGTGTACACCAGATAGCCCA
CAGAAGAGCGGCGACACCCAATAAGATCAGCTTTCGAGCACTATTCCCAACGCCAGTT
AACCAATTCCCCAGCATATGAGGTATAGACCTAGGAGGGGTTAACCCAGTAGCTATGTG
CATTATACGCCAGATAGTCTTAGCATGTTGGCAATGAAAGAAAAGATGATTAATTGTTTC
ATTGCAATTGCAGAAGCAACACTTTTGATTCCCATGCCAATTCCTCTTAGCTAAGTTATC
CTTTGTTAAAATCACCCCTCTTTGAAGATACCAAAGGAAGATTTTTATCTTCAAGGGGA
GTTTCAGCTTCCAAATGATAGTATTCACAAAAGGGTAGCCCTGGTTCATAAGATAATTG
TACATAGATCGTACTGAAAAAAATACCATTTCTATGTAAATCCCAAGAAAAAAGTCCTTC
ATATTGGTAAGCTGAATATTAGCAATTTGAGCTACTAGATTAAGCCAAGCGGTCAATTTA
TCTCCCACTAAAGCTCTTCGAAAAGACACATTCAATGGCACCTGGTTCATAACATGTGC
CACTGTGACCTGGGGGTAATGGACTAAACTATACAGGCTAGGAAATTGTTCTTTAAGGG
ACTTAGAACCCACCCAATTATCCTCCCAAAATCTAGTTTGTGAACCATCTCCTATCATGA
AGGTACCACACCCTAGAAAATCTCCCCTAACCTTTAAAAGACCTTTCCAAAAATGAGA
ATCACCAGGTTTGATATGTACCTGTGATAAACATTTCGAACTCAAATATTTATTTTTAATT
AAGGTCTGCCAGGTTCCTTCTGCATTAAGAAGTTTAAATAACCATTTACTGAGAAGGCA
TTTATTTTTAATCAAGAGATCTGTTATGCCTAGACCACCTTGATCTTTGGGGCGACAAAG
AATATCCCACTTGGCAAGGCGGTATTTATGTTTCTCATGATTACCTTGCCAAAAGAATCT
TGATCGATAGAAATCAAGTGTCTTTAGAACACCTTTTGGTATTTCAAAGAAGGACATCA
TAAACATAGGTAGGCTACTAAGGACTGAATTTAGTAAAACAAGTCGGCCCCCATAGGA
CATATGTTTTGCCTTCCAAGTACTTAATTTACGTCTAAATCTTTCCTCAATTACACCCCAG
TCTGCATTGCAGAGTTTGCGATGGTGCATCGGAATGCCTAAGTACCGGAATGGATATGA
TCCTATTTTACAACCAAAAAGCTGAGTATATTCCTGCTCATAATGCTTTGCCTCTCCATA
GCAGAAAATCTCACTTTTGTGGAAATTAATTTTTAGACCAGATAATTGTTCAAAAACAC
TGAGCAGCAATTTCATATTCTTAGCTTGCTCCAAATCATGGTCTATAAAAAGGATTGTAT
CATCAGCATATTGCAAGATGGACAATCCATCATCCACCAGGTGAGGTATGATTCCTCTA
ATTTGGCCATCCTCTTTTGCCCTCTTAATCAAAATTGAAAGCATGTCTGCCACGATGTTA
AACAGAATGGGTGACATAGGATCTCCTTGCCTAAGACCTTTTTTCGTTTGGAAATAGTG
CCCAATGTCATCATTAATCTTGATGCCCACACTACCTCCAGTTACAAACTTTTCCACCCA
ATTACACCATTGCTGGCAGAACCCCTTCATTCGCAAGGTTTGTTGTAGAAAGGACCATT
TGACTTTATCATAAGCCTTCTCAAAGTCAATTTTAATTATGACACCATTTAGTTTTTTGGT
GTGTAACTCATGTATGGTTTCATGCAAAATCACCACACCCTCCATGATATTTCGGCCAGG
CATGAAAGCCGTTTGAGTAGGTTGTACTACCTTACTAGCCACTCCCATTAGTCGATTTGT
GGTGACCTTTGTAAAAATTTTGAAGCTCACATTGAGTAAACAAATAGGTCTATATTGTT
GGATTTTTGTTGCCTCTTTAATTTTTGGAAGTAGGGTAATCACTCCAAAATTGAGGCTG
AATAAAGGAAGGCGATCTGCAAAGAAGTCATTGAAGAGCGCCAAAAGATCATCTTTTA
TAATTTCCCAAAAGACCTGATAAAATTCTGATGGGAAGCCATCAGGGCCAGGAGATTTA
TTGTGTTCCATTTGGAACACCGCCTCTTTTACTTCTTTATCCGTAAAAGGAGCGATTAGA
ATGGCATTTTCCTCCTCTGTGACCTGTGAGATATCTTGTATTTGATTCTCCAGTAAGCTA
AAATTGTTATCAACTGAAGGCCCAAACAACTCTTTATAATATTTAGTGATATATGCTTTTA
ATTCAGCATCACCCTCTATTATCCCATCCTCTTGTTCTAGCTTGAAAATGCGTTGTTTTCT
ATGTTTACCATTAGCAACCAGATGGAAATATTGGGTATTATTATCACCCTCCAAAAGGTC
CTTAGTTTTTGCTCTCTCATACCATTTAATTTCTTCCTCCCTCAACAAATGGACTAATCGT
TCATTGAGGTAGTGTTTGAAATTTAATTCTTGGTTAGAGAGAGTGGCTATTTCTGCCTTT
TTATCCAAAGCATCAATCATAGTGATTAATTTATTCTTCTCTTTTTTATAATGGCCAGCTAT

ATTTTTGGCCCAGCCTCTCAAAAATTGTCTAAGGGATCTTATTTATTTGCCATCTCTCT
ATAGCTGCGGCACCATTACTTCCTAATTGCCACATATTAGCAACCAGATCATAGAAACC
ATCTCTTAGTAACCATCCTCGTTCAAATTTGAACAAAGGACGTTGCCTATTATGAGAAG
AAGCACCTGTATTCAATAACAAGGGTGTATGATCAGAAGTACTTCTGTCCTTGGCCATT
ACTGTTGTGAGCGGGAATTTTTGTTCCTATTCTGTACTAACCAAAATCCTATCTAGTTTC
TCAAAGGTCGGATTATACCCGCTACTCGCCCAAGTATATTTGCGACCCGACATTTCTAGT
TCTCTGAGTTGTAGAGTCTGAATGACCATATTAAATAAATCTGGCCATCTGGCATCAAA
ATTATCTTTGTTTTTATCCTCAGGACAACGCATAATATTGAAATCTCCACCAATAATATAA
GGTAGCGATTCTTTACCACAAGTCTGGGCCATCTCAGTTAGAAACCTTTCCTTATATTGA
TGTTGTGCCGGGCCATAAACTGCAATCAACGACCATTTGAACCCATCACTCTTATTTCG
AAGAGTGAACTTAACATAGAATTCCCCTTCATCAATAGTACCAATGTCATATACCATCAT
GTCAATACCGAGGAGAATTCCCCCGGATCATCCATGTGGTGCCATGCAGTGCCAAATAT
AGTCCTTGCCACCACATAGTTTTTTAGGGTCCCATCTGGGAATTGATCCCTACCCGTTT
CCATGAGAGCAATAAAATCAATTTGCTCCTCTTTTACAATATCTTCCAAATATCCATGTTT
AGCCAAGTCTCCAAGACCTCTACTATTCCAGAAGATCCCTTTCATTTGAGACTCAACTT
GGAAGGTGTAATAGGTTTTTAGGTGTTAATCCCACCTTGTTTTTCTTTCCTGAATTATAT
TTCTTCCTGCGAGGAATAGCATTAAGCTCACAGCATATGTGATCATATCCCTCTTCATGC
ATGTTCTCATCAAAATCCTCACATGCAACATTGAGTTGTGCATCTAGAATATCTTCTTCC
TCATCCAAATTTATCTTAGGAGAGGAAGATTTATTATTAGTTGTTTTATGGATGTACAAAT
TATTCTGCAGAAACTAACACCATTAACTTAATTAAACCGAATTTTTCCAACCATTTGAAT
TCGCATTCAAATAGAATTAAGAAAGATATGATAGTATGATTTAGCAAAATAAAAGTTAG
ATTTAATGAGCAGGGAACAACACGGCCACGCATCACAAATAACATGTTTCCACATGAA
TACGCATGTGAGTGCACACGTGCATGTGTGTGATTTTAGCAATTGTTCTCCTCATGCCA
CCACATGCTATATGTCACATGTTTTAAAGGGTAGCAAGAACTCTAGTTTTATATTACACT
CCTACTTGTACAAGCAATGTGTGACTAAACCATACTATTCTTTAGAATTTTTAGGAATAA
ATTGGCATAATACTCATGGGTCCAATACTAATATGAGGTCAGCAAGTTCACTGGCCACT
CGCCACTGCCTTCACCCTCCCTGACGATAGCAAGGTCATACAGAAGTGTTGGGATACA
GATAGGCTACACTAGTGCAAATTAAAATTTCTACTGCGTAAACTAGAAAGCCATACCAG
TAAGAGATCATATATCATTAGCACTAAATGCATAGTGTAGCAGAAGAAGAGTCAAAATA
GCCTAGTTCAATGTAGCACATGTCAATATAGTGGAAATAGTCGATTGTCCAATGTTTGTG
AAAAGCGATCTTTGGAAGCCAGTCGATCTCCACATGCCAATTGTCTTTTCGTTGAATCA
TCGCGATTAGTGTTGCAGTTGCATCAGTGCTTTACCGAAGCCCACACGTTCTAGGGATG
AAACGTTGTGCGCTGGTGTGCTAGCACTAGCGCACATAATAGGGTCTTTCGAAAGGAG
ATGGCTGGGGTTTGGTAGGAGGTGCAATGTCTCCCACTATCTCACACATAAACCCATAG
CCACAATACTCCCCTTATATAAATCCTTCAGCCGAATCATAATCCAAAATGAGTAGAGAT
TATATCCTACTTAGAGAACATCCGACCAGATCCTTATCCAATACGAATAGATATTGCATCC
AACTTGAAAAGTCTTTGACTAGATCCTTTACTATTCAAATCTATTAAATGTGTGAGCTTA
TATGTTCATGTATATTTGGTTGTACGCTGAATACATCTTTCCTATCACTAGTCTTTGGTAG
CGGTCTCTAGTAGGACATATCGACTCTAAATATACATAAAGATCAATCTTGATGAACCTT
ATGTAATATGATCTGCAACTAAGTCTTGGTCTCTCACAATTAGTTCATAACTCTTTATAAC
CAAATTGTATCGGTTACGACTAGTCCGTATCCCCATACGACCAGTTCGCAGAATATTTTA
TGATATGTTTGACTAACTCATGACCAGTCAGTATATCCATACAACCAATTTGTGTCAGAT
CATACTTAACAACTTGTTTGTGTACTTTTATTTTGCCTCACGAATTTGATCGAACTTCATA
TGAGAAACGTATCATTCATGCATTGTTCAATCTTTTTCCGATCTAGTGATAGATAACTTTA

TGTAGTCAACTCTTCGATCACTCATGGTATGACCATGCATTTCCTTTATCTGACCACATC
GAGGGGTCTAGAGATATCTCTCGTGTTATGTAGGAGGGGTAAATTCCACACTCGATCAG
TCATATTCCTCTGCATGTTTTATAGCATACTCAAAAGATATCTTTATAACTACCCAGTTAC
ATAGTAACGTTTGCTTGAGACCAAATGATAGCATATAAAAATATATTACCATGTTCTCTA
ACATTTGTCTATATTATTAATTTGATATCCCTATAACTATGACCGGTGAAAGGTGGTCATC
AATTAATATATGTGCTAGTCTATAAGTCACTAGTGTTCCGATCAGTGATATCTGACTAGG
GATCTTTTAGAATAACACCATGATAAAAAGCTTCATTAATAAGTCATATACTTGTTAATAT
TTATAAATGATAATTCGTTGGAAGAACATAATAATATTTACTAAAATAATCATAAATATAG
AAATAGTATATTCAATTTTATGATCGACTCTAGAACATGTCGCTAACAAAAGGCTGTCAT
CGCTCACAAAGTCGACTTTCTGCGAGGGAGCTGGGCTCGCATGACTCGAAGGGCTCCT
CGCCGAGGAGGCTGCGGCGGGGCAGAGGCTCGGTTCGAGTTCGCGTTCGTGGACGCG
GACAAGCCCAGCTACGGGACGGTACCACGAGCAGCTTATGAGACAACACGCTGTGGG
ACGGCACGATGGCGTTGCCGCCCGATGTGCCCATGTCGGACCGCGACAGGCGGTGCTC
GGTCGTGACCGATAAGCGCGTCGAGGTGTACCTGCTCGCCATCGCCAACAGCGTCACC
ATCTGCCGCCGCCTCATGTGACCGACACGGCGACACGAACAGTGGCCACGACTCAAC
GTTTCGGTGGTTTCAGTAAGATTTCACGAGTTGAGGGACTGAGGGTGGAGGGTGAGTG
TAAGAGTGATCGATCACCGTGTTTGGGATCGACTCTTTTAAAAGTTAAAATAGTTTCAC
GATCGTCAGCTTATGAGATGGAAAAACCGTGTCAAGAAGAACGTATGGTTGCCAATGC
AAAATGGGTGCAAGCATGAAAATACTTGGAGGTTGGCTCGTAAGCCCAGCCTGTCAGA
CCGAGGCGGCGTACACACAGGCCGAGTGAAATGAATTCCTCTCCCAAAAAAAGTGCG
AGTTGAGTGAATCAAAATGCCTGCAATGGCAGCCGATTTTCGGAGTATCAGGTAACAT
GCATAGTGCATTAGAGGCTTCTCTTTACATTTTAGTTAGCTTTTAGCTTCTTGCCCACAC
GAAGAACAAGAGTGGGGCTCATCATTTTTTTTCTTCTCGGGGGGAAACTACATGAAAC
TTCCCAAAAGTAGGCACTGAGATGTAGTATCTAGTGCACATCTATGTTTAAGACATGGT
ATCATGCCCTGATGTAAGTAGTTATGACCGCAACATAAGTTCCTATTGTAGGCTGGATCC
TGTTGTAAACCGTACAGCCCAAGACTCGCTCAAACCCAGCTCCCAAGTGGAATGGGCC
TACGCATGTGCAGAAATCGTTTCGATTTTCTGATCGTTTTGCTTAAAAGGCTTAAGCTAT
TGTTTTGGACGAATAAACATTATATGATCATTTAATTCATTGCAAATTGGTAGTGTGGTG
GTTCTAAGGGACATGATATTACACCCGTGTTATGAAACACGATGTGAGTGCACATGATG
ATGTGAAGAACAAAAAGGTGGAGGAATACCGTAGAGTGCCAGATAATAGCAACTAAG
CAACCACAGCAAAAAGGAAAGAGTTAGCAAAGCAATTAGGTAAGCACTACTTCGAAA
GGTCATAGGTTCCCGTCCCTACTTTTTTAAAAGAAAAAAACTATACTTCCCGTCCATATT
TTCTTCTAGGGTAGTGATAAATATCACCCGGATGTTTTCTTTCTAACTATCATCTAAATTT
ATGACTATTAGATTAGAGATCTATGACCATAAATTCTTCCCCACCCCTTCCAAAAAAAAT
CTCCCTCTCCTAAAAAACAATTCTTCTGATGTCCACAGCAAGTTCAACAGCAGTAACA
AGACGCTGCTCAAGAGCGAAGCCCTGTACCAGTGTAGGACCGAAATGGCGACAGAGG
GGGGTGAATGTCGATCGCTTCAAAAATGTCCCCGAGAATTCTTACACAGATTTTTCTAC
AAGAAGCAAAACAAAGCACCTCACAGAAAATTAAAAAAAAATTTGCTGAAATTTGGA
ACGCATGCAACACTGAAAAATTCACCATAAATCAGAGATCATCCAGAAAAAATTTCAA
CTGAAAATATCGATGTTTGATCTGCATGCAAATTGGTCAAAGACAGCGCTAAAAATAAA
AGAGAGACAACTAAAGCATCTTTTCATTTGGCCATTCGGTTTGGCTGGAGGGAATACTT
CCAAGAATAATACAAGTGCAGAGCAAAAAGGGCAGCAAGAACTCCTAGCCGAAACAC
AGAACAGGCTGCTCCAAAAACAGAGAAAATGTTGATTTCTAGAGCCAATCAATGTGGT
GGCCGAGTGGAGTTAAAAAGCAATTTTAATGCTGCAGTCCGATCTGCAAATAAAGGCC

AATATTTGCTGTAGCTGCCGAATGGGATTTGAGGCCCCAAAATGCTATCAGCACTGCAG
TAAACAATAAACACTATGCTGTCCAAAAACTGAACTAAAAGACTGAATACTGAAACTC
ACTTTAACCAAAGGGATTGATGCTAAAAACCTATTCTCCTCAGATCACAAGATCAAGAA
CCTGCTCCACATACTTCTAGGACCTTTCAAAACAAGGGAAGAGAACACAAACACAAA
AATTTCAGATTATGGCAAAATCTGAAACTTTTTGCAACCCTGAACCAAAACCCAAATTA
CTCCATGGTCTTAAGTCCATTGCACATGAGATTTTGCAGGTATGTTAGCAATAGCATAAG
GACTACTCAGACCAAAAATCGAGTCAATCCCTCAAGAGAATCAAGATTAGCAGCAAAC
CCTAGAAACTGCAAAACATGAACAGGGACCTTTCAAAAATCCAAATTTCCAACTCTTT
ACTGATCTTTTGGCTATGAGATTTTAGAGCTAGATACTAGATAGCCTCAGAGCTCTTCAT
ACCAAATCTCATGCCACCTCTCCAAAGGAATCAAGAGTTCCACCAAACCCTAGAAACT
CAAATGCAAGAACAGAGGAACTCAAATGAGGAAATTCGTGATTCATCTTGGTTTGCTC
TTTGGATCAACTAGCCAAATGGCTTTTGAGCATCTCCCATGTCATAGCAACCCATTTTG
GGCCTTTCCAACAAGAAACAACAGGAAACACAAAATCTCACAAATCAAGTCAAAAGA
GATATCGAAGAACAGAGGGCACCAGAGAATAAACACTTCAGATTTCCATTCATTGGAT
CAAATGGAATACAAATACGTCCCGATACGCCTGGACATTAGGACATAAGAAGTGATTAC
ATGATCCTCAACTCATCCTCACCCTCTCCCACACAAGAACACACCCATACATGATTGGA
GAAGAGGAACACCACTCTGTTCTATCACCACTCAAGAACACATCGTAGGGGACAACA
GAGGGAGCACAGCCCTGCTGTATACACCAACCCTCCACCAACTAAAGCCCTAAAGCCA
AAACAACCACTAAAGCCTATTTTGACTAAAGGAAACCCTAAAACTCTTGGAGTGCTCA
AGTGGACATGCAGCCCCTCCACTCCTTTTATAGCCCATGGCTAACGGGTCTCCGCCCCG
TTACGCCCGCTATTTCCAACGGAGCCCGCTCCCGCTTGTGCAGCCTTGCGAAATTCCCG
TTTCGCCCCTCCTGCACCTTCCGGGAAGCTTCCGCGCTCCTTGTCTCGCACGCCTCGCA
ACCACCTCGAGTCCACGTCGTCTTCGACACCTCCTGACTGTTCTGACCTCAGCGCCAC
CTGCGCCTAGTCCAGTCCTCCGTGTCTCGCCACGACTCCGTCTCGACGTCCTGCAGTC
ACACAGACAAGAAACACGTCACATAGCACCGCTCGATACATGAAAGCATAAGTAACAT
CATACTTACCATGACGAAGTCCAAACCTGTCGAATCATCGGAATAAACAGCCTAATCCC
ATACAACCAAGTTTGACCAAAGTCCGACCGAGTCCAATTGCGCCACGCAATGACTCGA
CTCCGAACCACTCAAACCAAACTCGACTATCCTAAACCTTTGTCAATTACTCATCATTC
ACATGGAGTCACGACCACCTGGTCTCTCAACCAGTTAACACACAAAGTAACACGACA
GTGACACACCTAGCTATGTCAAGAACATTCGACGAGTCAGTGATCATTCGTTCCTCTCT
TTTTCTGCTGACGAAGATATGGCCGGCCATTTGCCGTCGACGTGCAGTATATTCTGGAC
ACGATGGTGCTGCCGCGCGAGACGGAGTGCATGCGCGAGCTGCGCCTCCTGACGGAC
AAGCACGAGCGGTACGTACGTACTGCGCGCGCTATACATCTCACTCTAGTTTTTAGTAG
CAGTCGCTTTTCACTTGCATTGCCAATTTGCCCAGACATGCATGATGGATGATGCGTGT
TTTTTTTTTGCTTAATTAATTTGCTGTTGTTTGGTCTGTGTGATGAAATATATATTGGCAT
GTGTAGGTGTTCCATGGTGTCGTCCCCGGACGAGGCCCAGCTGCTAGGGATGCTGATC
AAGATGACCGGCGCCAAGAACACCATCGAGGTCGGAGTGCTCACAGGTCACTCTCTC
CTCGCCACCGCGCTCGCGCTCCCCGACGACGGCATGGTGGTGGCGATCGACGTCAGCC
GGGAGTACTTCGACCTCGGTCTGCCCGTCATCCAGAAGGCCGGCGTGGCGCACAAGG
TGGACTTTCGCGAGGGCGCCGGGCTCGCGAGACTCGACGAGCTCCTCACCGAGGAGG
GGAACGAGGCGCGCTTCGACTTCGCGTTCGTGGACGCGGACAAGCCCAACCACGTGC
GGTACCACGAGCAACTGTTGAGGCTGGTGCGCGTGGGCGGCACCATCGTGTACGACA
ACACGCTGTGAGGCGGCATGGTGGTGCTGCCGCCCGACGCGCCCATGTCAGACCTGG
ACCGGCAGCGCTCAGCCGAAATCCGGGATCTCAATGCAAAGCTATCGCCAGTTTTGTT

TCGGCTGATTTTT**TAG**

>DfCCoAOMT9 (DfaB07G011900)
TTATGCCATACAATGAGAAATTTCATTGGATTTTAATTGTAATTATACCAAAATAGAACA
AGGCTATATAACTTGATTCTCTAAGTAAGAACAAATCCAAGTACACAGCTATCGCCCAA
ATACTCGATAGGTACGTACATTGTACATTGTACTCTTTCTTTCCTTCCGAATAACTAATCT
AACTTTTTCGAATAACGAAGGTCGTTCGCCACATACGTTCAACAAACACGTGACTCGA
CGACAGGGGCCAAGGCCTTGAAACATGATCGCAAGTTTCGGGTATTCGCTAGCTACAC
ATTAATTAATCATATACAACACATAGTAGTAATGCCACTTGATTTAACACATTATATTTTT
GCAGTGCCACCAGCAGGAGCCGGGCAACACGTGTGGATACTACGTGTGCCACCATATG
AGACTACTCCTCGATTCCACCAAGATCACTAATTCTAAGGTTTGTCAATCTTCCCACATT
TATTTCATACATGTCAAGTTTTATATATGCGCGCGTTGATATTCCTATCGACAGACTGCGT
CCATCTCTACATCTGCATTGACACAGCCGAATTTTGCAAAAATTCGAGGAGAGCTCATT
TGGTTTTTGTGGACAAAAGTCGTAAGATCAGATGGAGAGTTCCACTGCCCAGAATGAT
TTTTTATTGTAATTCTAAGTGCAAATACAATGTAGATCTTTTACAATTGTGTATTTGTTAA
TGGATATTTTATTTCAATTCAATGAATTGATTTGCGAAATGGATGCTTTATGTTTGTGAAA
TGAAGTGCGTTGTCAATTGGATGTTTTATGTCTGTGAAATGAATTGAGATGTGAAATGT
ATCTGAAATGAAATGAGATGTGTGAATTGTATATGTTTTATGTCTGTGATTATCTGCTTGT
TTCAGAGGGCATGGCTAGCATTTCCTGAGGCTAGCCAATAAACAGAAATATATTTAGGG
GTTGAAGGGAATATCACAGACGGTTTTGAGTTAAAAACCGTCTGTGATAGCTGATAATC
ACAGACGGTTTTTAACTCCAAACCGTCTGTGATTATCAGCTATCACAGACGATTTTTAA
CTCAAAACCGTCTGTGATTATCAGCTATCACAGACGGTTTTTAACTCAAAACCGTCTGT
GATTATCAGATATCACAGACGGTTCAAAAACCGTCTGTGATGACCGTCGATTATTACTG
ACCCATAAATACAGACGGTTCACAAAACCGTCTGTGATGACGATTTCGAACCGTCTGT
GATGACCTTTTTCTGTAGTAGTGTACCCTCTTGACAACTCTGCATTCGTACGGCATGATG
CTCTTGCTGATTAATTTTCTCAGCACGCGGGGTATGCTTCCTTTAAATTACAGGTTTTTC
CTCCGAGCGCGCAGTGAACTAACAAATAAGGGTTTTATTAAACGTCATCTAGATGTTTT
CCCGTCATCCATCGCGATATCACACCCAAATCGCGGTGAATGGAGCATTGGAAGATTCG
AATCTGCGCTGTCGTTGAAGCAGCAGCTTCTTGGAAGGACCGGGACAGAAAGCTAAG
AGGTTACTTTGCCATTTTTTTCTAGTCAATCCACGATAAATTGAGTGCGTAGGTTGCAG
CGGTTGGTGGCGGTAGGAACGTGATCGCGCATGGACCGGCACGACGGGAGGACGAGC
ACGCAAAGATTAACAAAAACGCTTGACAGTACTGCACATGCATTGATCAAACGCCACC
GTTTGGTCTGGCGATTGGTCGGCCAACAACCAAATTATGCAAGTGGTTGATTTCTTTTC
AAAAAGGAAAAGAAAAACATCACCTACACACATAGTGGTTGATTTCTTTTCAAAAAGG
AAAAGAAAGACATCACCTAACACGGTGATGATTTATTAGAGAGAGAGAGAGGGGGGG
GGGGGGGGGGGGCTAGTGGGAAGAGCGAGTGATCTGGCGACACCAACTCCTCCGTT
AGTTGATATTGATAATATAAATAACACGCCTAGCTAAGTAGTGGGTGGACACGCCAACC
CGCACCACCAGCTGAAGCAGAGGAAGAGCTCAGGGAGGCACCTAGCCAGCCAGGCA
GCCAGAGAGATCGGACC**ATG**GCGACCGGCGGCGACAGCGTGCCCAACGTCCACAGCG
ACATCGACAGCAGCAACAAGACGCTGCTCAAGAGCGACGCCCTGTACAAGGTAAACA
CACTAGCTAACACGCAGATCGAACGTAAGATCGACGAGCCATTAGTGAACGTTCGTTC
TTTCTTTGCTGATGGTGGTATGGTCGTTGCCGCTGACGTGCAGTATGTCCTGGACACGA
CGGTGCTGCCGCGCGAGCCGGAGTGCATGCGCGACCTGCGCCTCCTCACGGACAAGC
ACAAGTGGTACGTGGTTTAATTACCTGCTAGCCTCTCGTCTCGCATGTTCTTTTGTGGTT

AGTTCGATGTTGTTCGGTCAGTTGGGATTCGGTGTGATGAAATCGTGTCCTGGTAATTT
CAGGGGGTTCATGCAGTCGTCGCCGGACGAGGCGCAGCTGCTGGGGATGCTGATCAA
GATGGCCGGCGCCAAGAACACCATCGAGGTGGGCGTGTTCACGGGCTACTCGCTCCTG
GCCACCGCGCTCGCGCTCCCCGACGACGGCAAGGTCGTGGCGATCGACTACGACGGC
GAGAGCTACGAGGTGGGGCGGCCGTTCGTCAAGAAGGCCGGCGTGGCGCACAAGGT
GGACTTCCGCGAGGGCTCCGGTCTCGATCGGCTGGACGAGCTCCTCGCCGAGGAGGC
CGCGGCGGGGCACGAGGCGCGGTTCGACTTCGCGTTCGTGGACGCCGACAAGCCCAA
CTACGTCCGGTACCACGAGCAGCTGCTGCGGCTGGTGCGCGTGGGCGGCACCATCGTG
TACGACAACACGTTATGGGGAGGCACGGTGGCGCTGCCACCCGACGCGCCCATGTCG
GACCTGGACCGACGGTTCTCGGTCGCCATCCGGGACCTCAACGCGAAGCTCGCTGCC
GACCCGCGGATCGAGGTGTGCCAGCTCGCGATCGCCGACGGCGTCACCATCTGCCGCC
GCCTCGTG**TGA**CAGATACAGCGGTCGGGTGCTGTGGTCTAGTTCTTGCTGCTTTAATTT
CGTTGAGCGTTTGGTGATTTCAATAAAATTTCGCGAGTTGGGAC

>DfCCoAOMT10 (DfaB07G011910)
CGCCGATATTCACCAACCCTTCCTATTTTGGATGGCACGAAGATCGAGATTTGATGGAA
AAAGCCTTGCGTCCATTGGTGTTGCTTGATGAGGCGTGCGAGAAGACGCCTAGTTGAT
GACTCGGCAGAGTACGACTCGCACGGCGAGGAGCGTTTCTCTTGAGATCGAAGCCAA
CTCGGGAAAACTAAATCGAATCAGAGCCTGCCGGTTTGGGCTAAAAAAATATAACTTG
TAATTAAAATTATACTATAATTGATAATATTTTATTTGATATAATTAAATTTAAAAAATTAA
AAACAAGTTTGCCTATTAGAGACATGCAAATGAATATAAAAAAAGAACTGAAAAAAGC
TAGGAAGCCACAAAAAGACAAAATTTAGAAATAGTAAAAAATAAAGAAAAAGTCGGA
AGCTGAAACCGAAATGAGCGAGGCATCAATCTCCCTTGTGGAGAACGAAGCCGACTT
GTCTTCTCTAGCTTTAATATCAATGTTGGATTTTCTCTAACCTTAAATAAATTTTGTGAAG
GCTTTCGGTCTACTCTCTATTCCTCTGTCTTATCCGTGATGCCGTTCACAAATACATAAC
AAGGCTAGAGGGTAGAGACCTATTTATAGACTCCACTTTCAGGTGTTATAAAGTGACCC
ACCTGTTTTCGGTATCCCATGAGACTCTTGAACCATGTCACCAACCACGATTAGACAAG
CCAGTTTTATAAGTCAAAAATATATTAGTCTCAAGCATACATGTGTGAGTCATGCGCCAT
AGATAGACTAGTTTTATGAGTCTAAAATATATTAGTCTCAATCACACATGTGTGAGTCAT
GCGCCATAGATAGACTAGTTTTATGAGTCTAAAATATATTAGTCTCAATCACACATGTGT
GAGTCATGCGCCATGTCAGGTTGTTTATGAGATCAATTCCAATAGGATCATATGGGTCAA
CAGCTGCAGAGCCGATCCTGCAAAACAAATCCAGCTATTCTGCCCTGTGCTGTGCCCG
GGTTAAACAAATGCATGCATATCAAATTCCAGTAAACCTGATCTCAATAAATATGTCTAC
TATATTCCAGCACAGCCGTCTTCGGCACCTCGAGTATTAATTTCTCGAGTTAAATTCCAC
ATCAAATATTCCCTATCACTTGCATCAATAATATGGATAATTCCACTTTGAGTAAAATAGA
CAATAACCATAATAAATATTGAAAGCCACATAAAATTTCCAATAGGGCGAACCCGTAGC
GGGGGGGGGGTGGATTTTCCTCCTCAGACGCCAGATCCGGCGACGGCACCCCTCTAGG
CCACCGGATCCAGCGCGCAGCAGAGCGGACTGTAGAGGAGAGCTAAGGGATGGCGGC
GGCGAGGGAGGGAGAACTAGAGCTAGCGGGGGAAAAAAGAGCGAGAAGGGAAGAA
AGGAGCGGCTACGGGAGCGGAGCTATGGATGAGCGGAAGTAATCAGTGCATTGAGATC
CGTGCGTTAGATCCAACGGTTGTGGAGCATTCCATCAAAACGGTATAGGGTGCTCTAGT
TTTTCTCTTGTAACTTATTATAGATAGTTTGAAAAACGTCTACAATAATCGTATGTTATTA
CAGATATATAAATTCATACGATTCCAAACTGTCATAACGGTTTTGAACCGTATGTGATGA
CCAGTTCGATAGTAGTGCATGCTAGTCTGCCATAAAAAACATGTATGCTAGTGTGCTAG

CTCCTTACATTAATATTTATGTCGCCAGCCACGCGTTGGTTGGTTTTATTCAAGACGAAG
GGATCGATAGTTCAGTTGGGCACAAGGCACACGCAATTAAGACGATAATTATAATAAGG
GATCGATAGTTCAGTTGGGCACAAGGCACACGCAGTTAACATCCTGTATTTTATGTAGT
ACTATCCCTGTAGGAATGAAGCGTCTTTAGTTTGTATTGGAGAGGGAGGGGGCGAGAG
AGAGAGAGAGAGAGAGAGAGAGAGAGAGAGAGAGAGAGAGAGAGATCATCGGAGCC**A**
TGTCGACGTACCGGCCGGACAGCAAGACCCTCCTCAAGACCGACTCGATCTGGGAGG
TACGTGACGACCACAGTTTAGCTCCCTTTCTTGCTCAGTTTGCCGAGTGCGGCATCACG
CGCTGCTGAGATTAGCATGCACGCATATATGCTTGCAGTACGTGCTGGACACGACGGTG
TATCCGCGGGAGCACGAGCGCATGCGCGAGCTCCGGCTCATCACGAAGGAGCATCCGC
GGTACGTGCCTTGCTACGTGAAGAGTATATATATACCAATCACGTACGGCCGGCGAGAT
TGTGTATATCAATTACTCATGTTGCGCGCAGCTGTTTACGTCAATTGTTGCTGGCCAGGT
CGTTCTTTGCGTCGTCGCCGGACCAGATGCAGTTCTTCTCCGTCCTGCTCAAGATGATC
GGCGCCAAGAACACCATCGAGGTCGGCGTGTTCACCGGCTACTCGCTGCTCGCCACCG
CCCTCGTCCTCCCCGCCGACGGCAAGGTCAGTTAGCTCACAAGACGGAATTAGTGGAC
CTTGACTACTTCATCCAGCCATGCATGCATGTTTAGTGTATAGGCCGCGCGTGCATGATA
TCTGCACAAGTTTGCATATGGCAGGTGGTGGCAATCGATGTGAACCGGGAGTACTACG
AGCTGGGCCGGCCGGTGATCGAGAAGGCCGGAGTCGCGCACAAGGTCGACTTCCGCG
AGGGTGACGGGCTTACGGTGCTGGACCAGATGCTGGCCGAGGACGGCGCCGGCGCCG
TGGCCGGCACGTTCGACTTCGCGTACGCGGACGCTATCAAGATGCACTACGACGGCTA
CCACGAGCGGCTGCTGCGCCTGGTGCGCGTCGGCGGGGTGATCGCCTACGACAACAC
GCTATGGGGAGGCTCCGTGGCGATGCCGCGCGACACGCCGAGCAGCTCGGAGCACGA
CCGCGTGGTGCGGGGTTACATGATCGAGTTCAACGCCATGGTCGCGGCCGACGACCGC
GTCGAGGCCTGCCTGCTCCCCTTCGCCGATGGCGTCACTCTGTGCCGCCGCCTCAAG**T**
GA

>DfCCoAOMT11 (DfaB07G011930)
CGTTTTTTTAGATCCAAGCCAACCCGGAAAAACAAAATCGTGCGAGAGAAACGAGGA
GCGTTTTTTTATTTCGAAGCCAACTCGGAAAAACAAAATCAGGGCCTGTTTTAAGTCA
AAAACTGGCAGCTTTTAACTTTTACTATAGTACCTAATTTTCAAACGTAGTATTTTTTTA
AAATGGGGTCTACAAGTTAAACAATTTACACGTTAAAAGTCCAACATCTGTACCAGCC
AAAATCGCAAATGAGTATCTATCTCTCCGTAATTTGTACCATAAACAGCCAAAATTTTAA
AAATTTCAGCCAAAAGTCATAAGTCGCAGCCGAAAAAAACAAGCCCTCAGAGCCTGC
CCGTTTGGGCTAAAAAAATATAATTTATAGCTAAAATACACTATAATTGATAGTGACTTAT
TTAAATAATTAAATTTAAAAAATTTGAAAATAGAAAAAAAACAAGTTAGCCTATTACAG
ATAATGCAAACGAATACGAAAAAAAGAACTGGCAAAAAGCCAGAAAACCAGAAAAA
GACGAAATTTAGAAAAAGAGAAAAATAAACCAGAAGTCAGAAACTGAAACTGAAATG
AGCAGGGCCTCAATCTCCCCTGTGGAGAACAACGCCGACTTGTCTTCTCTAGCTCTGA
TATCAATGTTGGATTTTCTCTAACCTTAAATTAATTCTGTGAAGGCTTTCGGTATACTCTC
TATTCCTCTGCTTTATCTGTGGTGTCGCTCACAAATGCATAATGAGGTTAGAGGGTAGA
GATCTATTTAGAGACTCCACTTTCAGGTATTATAAAAGTGACATATTTGTTTTCGGTATCC
TATGAAACTCCTGAACCATATCATCAACCACGATTAGACAGGCAATTTTTATGAGTCTAA
AATATATTAGTCTCAAGCACACATGTATGAGTCATGCGCCATGATCAGGTTGTTTATGAG
ATCAATTCCAATCTGATAATGTGGGTCAACAGCCTGTAGATCAGCCGATCCTGCAAAAC
AAACCCAACTATTCCGCCCTGTGCTGCGCCTGGGTTAAACAAATGTATGCATATCAAAT

TCTAGTAGACCTGATTTCAATAAATATGTCTACCATATTCCAGCACAGCCGTCCTCGGCA
CCTCGAGTATTAATTCTTCGAATTAAATTCCACATCAAATATTCTCTATCACTTGTATCAA
TAATATGAATAATTTTACACTGAGTAAAGTAGACAATAACTATAATAAATATTGAAAGCC
ACATAAAATTTCCAATAGGGTGAACCCGTAGCGGGGGAGGTGGATTTTCCTCCTCCGA
CACCAGATCCGGCGACGGCACCCCTTTAGGCCACCGGATCCAGCGTGCGGCAGAGCC
GACTATGGAAGAGAGCTAAGGGATGGCGGCGGCAAGGAAGGGAGAACGAGAGCTAG
TGAGGGAGAAAGGAGAGCCAGAGGGGAAGAAAGGAGCGGCTACGGGAGCGGAGCT
GCGGAGGAGCAAAAGTAATCAGTGCTTTGAGATTCGTCCATTGGATCCAATGGTTGTG
GAGCATTCCATCGAAACAGTATAGGGTGCTCTAGTTTTGCTCTTGTAACTTATCATAGAT
GGTTTGAAAATCATCTATGATAACCGCATGTCATCACATGTACATAAATTTATACGATTCG
AAAACCGTCATGACGATTTTGTACCATCTGTGATGACTATTTCGGTAGTAGCGCACGCT
AGTCTGCCTCAAATAAATATATACGCCAGTGTGCTAGGTCCTTACATTAATATTTATGTCG
CCAGCCACGCATTGGTTGGTTTTATTCAAGACGTAGGGATCGATAGTTCAGTTGGGCAC
AAGGCACATGCAATTAAGACGATAAATAAGGGATCGATAGTTCAGGGCACAAGGCACA
CGCAGTTAACATCCTGTAATGTATATGGTACTATCCCTGTAGGAATGAAGCGTCTTTAAT
TTGTATTGGAGAGAGAGGGGGCGAGAGAGAGAGAGAGACAGAGATCATCGGAGA
C**ATG**TCGACGTACCGGCCGGACAGCAAGACCCTCCTCAAGACCGACTCGATCTGGGA
GGTACGTGACGACCACAGTTTAGCTCCCTTTCTTGCTCAGTTTGCCGAGTGCGGCATCA
CGCGCTGTTGAGGTTAACATGCACGCATATATGCTTGCAGTACGTGCTGGACACGACGG
TGTACCCGCGGGAGCACGAGCGCATGCGCGAGCTCCGGCTCATCACGAAGGAGCATC
CGCGGTACGTGCCTTGCTACGTGAAAAGTATATATATACCAATCACGTACGGCCGGCGA
GATTGTGTATATCAGTTACTCATGTTGCGCGCGGTTTACGTCAATTGTTGCTGGCCAGGT
CGATCTGGGCGTCTTCGCCGGACCAGATGCAGTTCTTCTCCGTCCTGCTCAAGATGATC
GGCGCCAAGAACACCATCGAGGTCGGCGTGTTCACCGGCTACTCGCTGCTCGCCACCG
CCCTCGCCCTCCCCGCCGACGGCAAGGTCAGTTAGCTCAAAGACGGAATTAGTGGACC
TTGACTTACTTCATCCAGCCATGCATGCATGTTTAGTGTATAGGCCGCGCGTGCATGATA
TCTGCACAAGTTTGCATATGGCAGGTGGTGGCAATCGATGTGAACCGGGAGTACTACG
AGCTGGGCCGGCCGGTGATCGAGAAGGCCGGAGTCGCGCACAAGGTCGACTTCCGCG
AGGGCGACGGGCTCACGGTGCTGGACCGGATGCTGGCCGAGGACGGCGCCGGCGCCG
TGGCCGGCACGTTCGACTTCGCGTACGCGGACGCTATCAAGATGCACTACGGCGGCTA
CCACGAGCGGCTGCTGCGCCTGGTGCGCGTCGGCGGGGTGATCGCCTACGACAACAC
GCTATGGGGCGGCTCCGTGGCGATGCCGCGCGACACGCCGAGCAGCTCGGAGAACGA
CCGCGTGGCGCGGGGTTACATCATCGAGTTCAACGCCATGGTCGCGGCCGACGACCGC
GTCGAGGCCTGCTTGCTCCCCTTCGCCGATGGCGTCACTCTGTGCCGCCGCCTCAAG**T**
GA

>DfCCoAOMT12 (DfaB07G012160)
AGCATATGGTATTACAAGTGTAATATATTCTAGTTATCACAACTCAACATGAGAGATCTA
CTGTAGCTCTGCTCCATTGACGGTACAAGAGTTACTTTCTTCTAAATTTTTTCTATAGGA
GCAGTTTTCAACATGTCATGAAATTTAGTTACTGTCGACAGTTTGATGTACATGATTCAC
ATGTGCAAGTTACTCTATGATAGACATTGTTTGCTGAGTTATATGTTCTAGTGGAGCATG
TAAAAAGTTCATAAGATAAGTTCTTTACTATAGTTATCTAGCGTCACAAGAGAAGTAAA
GACCGATCCAGTCTTACTCATCGATTAGTCCTATGGACTTTTAACACATAAGTATCATTT
ATGGTGGATTATGATTTAAGGATATTTAATTTGTTTTGGCTACATGAGGGAATTTGGCTTA

ATATGGAGATTGTTGACTTTTTTTTATCCTAATTCTATTTGGCCTAATTTCGTTTTCAACA
CCAAGTTTTACTTTTACCTAAAGTTCCTACATAATTACTATAAATATATTCCTGTAGACCA
TAAAAAAAGTAAGAATCTCATTGTAAATATCTCTTTATATAGTAATTTTCGTCTTCATGTG
TTTGTAGTTTTTTGGTATCTTCTATTTTTTCTTTTAAACATCTATTTTTATAGTGCATCTTA
GGACAGTTTGGAATGTATGAACTTCCTCGCTAAGTACACGAAATCAACAGAGTCCAAC
GAATGATGTACTCGAAACGGAAAGATATCCCTAGATATATAATATAATAGCGTTGTTATTG
AGCCCAAAACAAAACATCATCGATGACATATTGCCGACAACTACATGAAACGGAAAGA
GATGCCTAGATATATAATATAATAGCGTTGTTGTTGGGTCCAAAACAATACATCATCGAT
GACATGTTGCCGACAACTACATTTCCTGGCCTATTCCATACTGCGGTCTGCCTGATTCCT
CAATAGCCCAAAGCATGAGCATGTAATACGCCACAATTTGGTCCCCATCATATTCATACA
TCCATGGTGCTGTGTTACGATTTACGGGGAGGATAACACTTTGAGAGATTCGATTTTGC
TCTGTACTACTTGGATGAAGTTTCCGAATAATAAACAAGATGAAGACAGGGAAAGAAA
ATAACACCTTACGCTTTGATCAAAGGCTACCTTCAGGTAAAGAAAGTTATTTATTTTTAA
AAGAACTAGCCAAATTTCCACCACAATTGCACACGATGCATATATTTCTATCTACACATA
GAGTTGTAATTGCACACGATGCATATATTTCTATCTAAACATAGAATTGTACTTATTATAC
AACTTTATTGCTTACGATGGGAATACAACAGTGTTCATTATACAATTTTAGTAGGTCTTAT
ATCTGTATATGTTTAATCTGCACCGTTATAATCTGGAGCCACCTCAATCGACGATGTCGA
CCTTTCTTTTTTCTCCTTCAATTATTAACGTGATAATTTTTAGTTCTTGAGAACGGACGT
GGTAGCTTCTTTTTGTATGTTATAGTCTAAAGAGTGTTTTTTATGATAGAAAGACTTATG
GAAAGGGCCACCTCAATCAACGGTGTAGATCTTCTTTTCTTCGATTATTAACATAGTCAT
TTCTAGCCTCTAATAACTTGGTAGCATTTTTTTCTGCCGTTATAGTCTAAAGAACATTTTC
TTACTTATAGAAAGACTAATGGAAAGAGGGACCTAGAACCACCACCAAGAACATTTCT
TCCCTTACGCGTAAGCGTAAAACTGAAGATTGTGAGGCGTTTCCTCTTGATCTGGTATT
CCGCAAAGGAGGTGGATAGGGTCAGCGAATGAAGTGGCGAGGGTAGAAAAAAAAGG
TTTGAAATTCCAGGTTCCCGCGGTATTAAGCAAAGGACAACAAGTCTAAGTCTTTTGC
AGTAGATAAATATCACGCCGAGCTAGTGCGGCGGACACTGAGTGCAGCTAAAGCAGAG
CAAGAGCTCAGGGAGGCAGCGAGCGAGCC**ATG**GCGACACACAGCGACAGCGTGCCC
AATGTCCATAGCAACATCGACAGCAGCAACAAGACGCTGCTCAAGAGCGAAGCCCTG
TACCAGGTAACACACGGCACATCTATGTCAAGAGCGTTAATTTGACGAGCTGGTGAGT
GTTTGTTCATTTTTTTTTCTGATGGTATATATGGACATGGTTGTTGCCTTTGACGTACGTG
CAGTATATCCTGGACACGACAGTGCTACCGCGCGAGCCGGAGTGCATGCGCGAGCTGC
GCCTCCTCACCGTCAAGCACGAGAGGTACGTACTGCGCGCACTATTTATCTCTCTCTCT
CTCTCTCTCACATACACGCACACTCACTCACTCTTTTTAGTAGTAGTGGCTTCTTCTTTT
ATGGTCAATTTGATGTAGTTTGGTTGATCTGATGAAATATATATTGGCATGGTGCATAGAT
GTAACATGGCGTCGTCCCCGGACGAGGCCCAGCTGCTAGGGATGCTGATCAAGATGAC
CGGCGCCAAGAACACTATCGAGGTCGGCGTGTTCACTGGCCACTCGCTCCTCGCCACC
GCCCTCGCCCTCCCCGACGACGGCAAGGTCGTCGCGATCGACGTCAGCCGGGAGTACT
TCGACCTCGGCCTGCCCATCATCCGCAAGGCCGGCATGGCGCACAAAGTGGACTTCCG
CGAGGGCCCTGGGCTCGCGCGGCTCGACGAGCTCCTCGCCCGGGAGGCCTCGGCGGG
GCACGAGGCGCGGTTTGACTTCGCGTTCGTGGACGCGGACAAGAACAACTACGTGCG
GTACCACGAGCAGCTGCTCCGGTTGGTCCGCGTGGGCGGCACCATCGTGTACGACAAC
ACACTGTGGGGTGGCACGGTGGCGCTGCCGCCCAATGTGCCCTTGTCGGACCTGGACC
GGAGTCGCTCGGCCGAAATCCGGGACTTCAACGCGAAGGTCGCCGCTGATAAGCGCG
TCGAGGTGTGCCAGCTCGCCATCGCCGACGGCGTCACCATCTGCCGCCGCCTCGAG**TG**

A

>DfCCoAOMT13 (DfaC07G006610)
CTGCTGCATAGAAAGGGCCCACAGCTTGATGGCATCCTACAGCGATGCCTTATCAACAA
ACATCAAATGCAACCGAACCTCTGTCCCATGATACTCCCACAGTAATTCATGCTCGTCA
TATGCATAAACACTCTCAGCATCGAACCGGTTCCATTCCTGCGGGACAGGAGCTTCATC
CTCATCCGAACTATCCTCGTCGTCCTCCTGTACAGCTTCATCTTCAGCCCATGCCGTCTG
GTCATTGCACTCCATCTAGTGCACTATGTACTCTATATGCTCCCCTTCATCGGCTTGTCTT
GTTCCCTCACCATGGTGTGACCATTCTCCCTCTCCTCCATGTACCGTATTAGCCTCGTCA
CAAGTATCTTCCTTCTCTTTGCCAATATGCAAGCAACCCGAGCTGCTCGCACCTCCCAG
ATGATGGCCGTATGCACGACCTTGCACAGCTTCTAATGGGCATGGTTGTACAAGCATGA
CACACTCATATTCACGGCGCCGACACCCTTGCACAAACCCCTTCCATGTAACAGTGTGC
ATCAATGTTCTCAACTCCTATCTGATGTCCGTGGGAGACCGGCTCTAAAGCATTTGTATG
ACAATGCTGTACACCTCGGGGTTCAAACGAAAAACTGTGGGTAATCCAATGCATCACA
TCTGAGATTCGAGCATTCTCGCCGTCCGGGTGTACCAGAGTGGCTCGCGCAAAACTGC
TCAAATCCATACCATTCGGACCTTCACGAATGGTTCTAGCACCGTAATGCACTCTCAAT
GTAGTGGGTATCGGCATTGCTGATGTCTGCTTGCATATATGTTTTAATTTTGCAACATATC
AAATCGCATCGCACACTACGAAAGTACTGATGTACTGTAATTCACATTTGACCGATCGT
TCGGCTGTCGTAACCAACTACACTAACCTCGTAGTATAACCTCCTACGGTAGCCCACAC
ACCGATGCCGCACTTTTCTCTCTACTCGAACTGTACCCTACTGCAAATAATTCCTACTGG
TTTCGACAATTTATATGACATATCGATTTAAATGCCTATGTACCAGTTACAATGGTACATA
TGTTTTTCCAACATACTAACGATATTACATTCATTCATATTTAATCACAAATATTACGTATA
ATTGTTTTTACAAAGTAAATTATGAAATTCTTACAAAAATATTGACTGAATTTTGTAATAC
CTATAAATTTGTATGGACAATTAATTTGTACGTAACGTTTGCTGAATTTTCTACAACTAC
AATTTTATATGCATGCCAATTTGCTAACGATGCTAATTTTGTACATTTATGTTCACTAAAT
TATACACAACTACATGCTACTATGACTGCTAAATTTCTAAAATTACATTGACTAAATTTTT
TATACCAAAGTTCAACTATTTTTGTCTACATCTAAACTTTTCTACTCATACTAACCCTAAA
TAAAATATCTACTGCATATACAAAATTTCAAATACAAAAAATTACCTCACAAAGTGGCTT
CTTCCTCTCCTCCTTCATCTCCTCTCCTCTTCTCCTCCCTCTCCTTTTCTCTCCTCTCTTT
TCTTTCCTCTCTTTCTCCTCTCCTCCGCCGTCTCCTTTTCTGTCGCTGTCGCTTCTGTTC
AGCGCTGCGTGGCCCTTTTATCTGCGCTTCCATTCGCCCAACCCTTCGGCGACTGGAAC
GCAGTCGTCTAACGTTGGGTGACAGCAATTGCAGTCGCCTGACCGTTGGGCGACTGCA
ACAATTCGCCCAACAACCAGTGGATTGCTTGCGTTTCGCTTGACCGTAGGGCGATAGG
CTCTAGTCGGCCGACGGTTAGGCGAATGGAGTCTATTTTTGTAAAAAAAAATTTGATGG
AATATATTTTTATTATTTCGAATAAAAATAGAGTATTTGTAAAAAAAAAATCTCAGTTTGT
TACACTTGGCGACGCAAGGCTGACACTCTCGCTTGTTATATAGAATAATGCCACTCCTG
CGCCATTCGCTCACCGGACTTGCACGGCG**ATG**GCTGCGAACAGCGACGCGGCCGGCG
AGGTCAAGGACATCCACACCAACGGCAGCACCAAGACCCTCCTCAAGAATGACGCCC
TCTACGAGGCAAGTACAATTGTCTTTCCCTGAATCTTTCTTTCTCGTTCCAGCCAGTTC
GGCGCGTGGATCGAGATCTGCAAATGCCTGACGCGTACGCGCGCGCGCGCGCGCG
GTTTTCCTGGTCTTCTCTATTGCAGTACATGTTGAACACGATGGTGTACCCGCGGGAGC
ACGAGTGCCTGCGCGAGCTCCGGCTCATCACCAAGGAACACACCTAGTACGGACGCA
CGCCTAATTCGTTTCTTTTTTTTTCCTTTGATCTCGCGTGATTAGTTTAATATGGCCATGC
ATCACGGTGGATTTGGTTTTCTTTTATTAGTGGGTTCATGTCGTCGCCGCCGGACGAGG

CGCTGTTGCTGTCGCTGCTGGTCAAGGTTGTTAGAAATTTAGGCAAATTACGACATAAT
TTAATCCAGAAATTACCAATTAAAATCATGACAAGGGTTGTGCTAGTACTATTCATTTTT
AGAACACTAAACAGTGTAACTAACTCTATGAACAATACTGTAAAGATCAGGAACAAAA
TGTTATACCCAGAGGCGGGGCCAGTGCCAGAGGCACCGGATGCGCCGTTCTGCTCAGT
TGACATAAGCAGCTATTCGATGTAGTCGATCTCAGTTGGTGCAGGACGAGGTACGCAG
TGCAGTCCCTTGAACGGCCTTCTGGAAGTAGTTGTACTTGCCGTGTAATGGAATGCCGC
CGGGAATTAGCCGATCACGTCGTGTATAGAAGCGAGCAGTCGTGCTGAGCACTCCCCA
AAAACCTGATCGCCCGCCTATCCGTGCAGATGTCACAGCTGGACGTGGTTTTGGAGGC
CTGCTCTCCCAAACGCCTGTGCGCGCAGGAGCTGGGACGGGGAAAAACTCGGAGGAG
CAGCTCAACAGAGAGGAGAAGGTGTGGTTTCTGTGTCTCGCTCGAGGCTACTGTCGAC
TCCCAAGCTTATACGCGTGCCAAGAAGATGAAGAGACAGGAAGCGTTACCGGGACAG
GGAGTAATCGCAAGTAACGGACAGTAACTCTCGCACAATTACTACTTGAAACCGACGG
AATCAATTCCGGTAACTGCTCACTTAATGCCTTCATCAATCCATGCACTTAGCATTTAAT
ATGGGCCTTAGAATTTATTTGATTTAATTGTTGAATTAAAAATGGACCAAGCTCAAATTA
AATCCAACAATCCCCACCAAATTCTAGGGCACATATGAAATACTCTCAATTCTCTGTTAT
TTGATATACCAGTGTTTCGATGGAGACTGTTAAGTTGAACATCCACCTAGAACAAAGGT
TACACTTTTTCACAACTGAACAATGGACTATGCCTTGAATTGACGGTTTTGTGCGAAAT
AAGTTTCACCTAAGTCCTTTACTGATACTAGGCTGCCTAAAGCATCCCCTCTGGTTGGA
GCATATAAGTCAAACTCCTTGGCCTTTCATGAGTATCTAGAGATCACCCAAATCTCATAG
ACTGTGACCAGCAGTCAAACTCATATAGGTGTGTGTTCCTCATAAGATGTTCTGTAGGA
CAACATCTCTGCTTCAACAAGCCACTCGGATCACATTAAGGTATAAACCAACCTGCCTT
ACAGGATAGGAGAGAAGTGCATCTCATACGACATGGACCTTGTTAAGGGTCTCTCCTC
ACAGTCTACCACTAGCTTGTTTCACCATCCTACTTCACGGGATCTCCGATCACATAGGA
CAGGTTACCACTATGATAGACTTCACGTGGGTCTCAAGCCCATCTCACTCGATGCACTA
TCTATCACATTACGTGACAAACCCTTAGTGAATTGATCTGCCAGATTCTTAGACGTATGG
ACATAGTCCAATGCTATCACTCCGGAGTTTCTCAATTTCCTGACAGATTTCAACCGCCT
CTTTATATGCCTTGTTGACTTCATGTTGTCCTTAGAACTGTTCACTTTGACAATCACCGT
CTGATTATCACAGTTCATAGAAATAGCCGGTATAGGTTTTTCAACCACCGGTAAATCCAT
AAGGAGTTCACGAAGCCATTCAGCCTCAACAGTGGCGGTATCCAATGCTGTGAGTTCT
GCTTCCATTGTTGACCTCGTTAAGATGGTCTGCTTGCAAGACTTCCATGAAACAGCGCC
ACCTCCAAGTGTAAACACATATCCACTTGTGGCATAAATCTCATCAGCATCAGATATCCA
ATTGGAATCACTATAACCCTCAAGTACCCTTGGATACCCAGCATAGTGAATCCCATAGCT
CATAGTACCTTTTAAATAGCGCATCACTCTCTCAAGAGCACGCCAGTGATCATCTCCCG
GATTTGATACAAACCGGCTTAGTTTGCTCACAGCAAATGAGATGTCAGGTCTCGTTGCG
CTAGCTAGATACATAAGTGAGCCAATGATTTGGGAGTATCTTAATTGATCCCTTGCTATT
CTTCGATTTTTCCTTAATAGCACACTAGGATCATAAGGCGTTGGAGCAGGTTTGCAATC
ACTATACCCAAAGCGACTCAACACCTTTTCCACATAGTGAGATTGCAGAAGTGTCACC
CCACCATTGCCTTCTCTCAGTAGCTTGATATTAAGAATAACATCAGCTACTCCCAAATCC
TTCATCTCAAAATTATTGGACAAAAAGTCCTTAATCTCCTTAATCACATTAAGGCTGGTT
CCAAAGATCAGTATGTCGTCAACATACAAACACAAGATCACACCTTCACCCCCGCCATA
GCGATAGTACACACATTTATCAGCTTCATTCACAACAAAGCCAGCAGACGTTAATGTTC
TATCGAACTTCTCATGCCATTGCTTAGGCGCTTGCTTCAGGCCATACAAAGACTTCAAT
AATTTACACACCATGCCTTCTTGACCATTTGCTACAAACCCATCTGGCTGATCCATATAA
ATCTCCTCCTCTAACTCCCCATTTAGGAAAGCTGTCTTAACGTCCATCTGATGAACGAG

AAGACCATGAGAGGCTGCCAAGGAAAGTAATACTCGAATTGTGGTCAATCGAGCAAC
AGGTGAATAAGTATCAAAGAAATCTTCGCCTTCTTTTTGGGTGTAACCCTTGGCCACAA
GCCTTGCCTTGTACTTTTCAATAGTACCATCAGGCCTAAGCTTTTTCTTGAACACCCATT
TGCAACCTACAGGTTTGCACCCGTAAGGACGATCAATGACTTCCCAAGTTCCATTAGAC
ATAATAGAATCCATCTCACTCCGTACTGCTTCCTTCCAAAAGTCAGCATCAGGAGAAGA
ATATGCCTCTTCAATGGTTCTTGGAGTGTCATCCACAAGGTACACAATGTAGTCATCAC
CAAAAGACTTTGCAGTCCTTTGTCTCTTACTCTTTCGAGCGACTACACTGTCATCCTCC
TCAGGATTTTGCACACGGGTTTCCTCAAAGTGTTCTATCGAAATATGTTGCTCATGGGG
TATTATAGGTTCATGACTAAACGTGCTAGGTGATTCTTTCATAGGAAATTCATTCTCAAA
AAATGTAGCATCTCTGGACTCCATAATAGTACCAACATGCATGTCAGGTACTTCAGAGT
TTTATTAAAAACCTATATCCCACGCTGTGTATAGCATAACCAAGGAAAACACAATCAAC
AGTTTTAGGTCCAAGTTTGCGCTTTTTGTTGATTGGCACATTCACCTTGGCCAAGCAAC
CCCAAGTGCGCAAGTAGGAGAGATTCAATCTTTTCTTTTCCCATTCCTCGAATGGTGTA
ATTTCTTTATTCTTTGTAGGAACTCTATTCAGGACATGACAAGCAGTCAATATAGCCTCA
CCCCACCATTCCTTAGATAGTCCCGCTGTCTCTAACATGGCATTAACCAACTCAGTTAG
AGTGCGATTCTTTCTTTCGGCAATCCCATTTGATTGTGGTGAGTATGGCGGTGTCCTTTC
ATGAATAATTCCATATTCCATGCAGAATTCAGAAAATTCATTTGAAAAATACTCTCCACC
GCGATCAGATCTTAACCGTTTAATTTTCCTCTCAAGTTGATTTTCTACTTCAGCTTTATAG
ATCTTAAAATAATTTAACGCTTCATCTTTTGATTTTAGCAAGTACACGTAGCAAAATCTA
GTACAATCATCTATAAGTGTCATGAAATATCTTTTGCCACCTTTAGTCAACTCGCCGTTC
ATCTCGCACAAATCAGAATGAACTAGTTCTAATGGTGCCAAGTTCCTCGCCTCTGCTGC
CTTGTGAGGCTTGCGAGGTTGCTTAGATTGCACACACACATGGCACTTAGAATCTTTGA
CTAAAGTAAATTTCGGGATTAAATTCAGATTTGCTAGCCGCGTCAAACAGCCAAAATTA
ACATGACAAAGCCGTGAATGCCAAATGTTCGATTCATTCGAATTAGTAACATTGTTTGC
AACATTATTACACACATCAGACAAAGATAAGCGGAACAAGCCTCCGCAATCATAACCTT
TTCCAACAAATGTCCCATACTTAGAAAGTACACATTTATTCGATTCAAAGACTAATTTAA
AACCATCTCTACATAGTAGAGAGCCGCTAACTAGATTCTTTTTGATAGAGGGGACATGC
TGCACGTTCTTTAATTGCACGGTCTTTCCCGAAGTGAGCTTCAGAATAACCGTACCAGC
ACCAAGAACACGCGCATGCGATCCGTTCCCCATCAGCAAGGTTCCAGTCCCGCCATTC
TGGTAAGAAGAAAACAAAGAAATATCAGCACACACATGAATATTAGCTCCAGTATCCAT
CCACCACTCAGGCGAATGACAGACTGAAAGAACAGTAGGTAAAGAATTACCATACCCC
GATGTTCCTGCATCAGTCTCGCTAACAACCATGTTTGCAGACTTCTTGTCCTGCTTAAAT
TTGCGGTCTGGGCACTCACTTGCCCAGTGATCCTCGCTCCCGCAAACAAAGCAGTTCC
CTTTGTTCTTTTTCTTCTTAAACGTGGTTGTTTGCTTAGTCTTTGGTTTGTTCTCTTGCTT
GTTCTTCTTCTTCTGGTTGCGAGAAGCATTGAAGTTTTTCTTCTGCACCAAATTGGCAC
TAGAAGACTCAACTCCTTTCCCACGAGTGTCTTTTGCCCTCGCCTTCTCCTCAACATCA
AGAGACCCAATGAGGTCAGCGACACTGAACTCTTGTCTCTTGTGTTTTAGAGAAGTGG
CAAAATCCCTCCAAGAAGGTGGTAACTTGGCGATAATACCGCCAGCCACAAACTTGTC
GGGTAACACGCATGGAAAATGCTCGAGTTCCTTTGCCAGTGCATGTATCTCATGAGCCT
GTTCCACTACAGATCGGTTATCAACCATCTTGTAGTCATACAGCTGCTCCATGACGTACA
GCTCACTACCAGCATCGGAAATACCAAACTTTTTCTCAAGTGCTTCCCACATCTCTTTG
CCAGACGGCAGTAACATGTAGACGTCCACTATGTTTTCAGCGAGAACGCTAACCACAG
CGCCTCGAAACAGGTTATCAGCAACCTTGAACATTTTCTCCTCCTCTGGAGTAAATTGT
TCAGGAAGCCCCTGCACGACGTGGTAAACGCTCATAGCCGTCAACCACAGGACCATCT

TAGCACGCCACCGCTTGTAATTTGAACCATCAAATGTATTCGGCTTAAGAGCAGCAGCA
AAACCACTAACCGAAAAGTGCCTAAAATCAGGTTTTTGGATTGTTAGAAATTTAGGCA
AATTACGACATAATTTAATCCAGAAATTACCAATTAAAATCATGACAAGGGTTGTGCTA
GTACTATTCATTTTTAGAACACTAAACAGTGTAACTAACTCTATGAACAATACTGTAAAG
ATCAGGAACAAAATGTTATACCCAGAGGCGGGGCCCAGTGCCAGAGGCACCGGATGC
GCCGTTCTGCTCAGTTGACATAAGCAGCTATTCGATGTAGTCGATCTCAGTTGGTGCAG
GACGAGGTACGCAGTGCAGTCCCTTGAACGGCCTTCTGGAAGTAGTTGTACTTGCCGT
GTAATGGAATGCCGCCGGGAATTAGCCGATCACGTCGTGTATAGAAGCGAGCAGTCGT
GCTGAGCACTCCCCAAAAACCTGATCGCCCGCCTATCCGTGCAGATGTCACAGCTGGA
CGTGGTTTTGGAGGCCTGCTCTCCCAAACGCCTGTGCGCGCAGGAGCTGGGACGGGG
AAAAACTCGGAGGAGCAGCTCAACAGAGAGGAGAAGGTGTGGTTTCTGTGTCTCGCT
CGAGGCTACTGTCGACTCCCAAGCTTATACGCGTGCCAAGAAGATGAAGAGACAGGA
AGCGTTACCGGGACAGGGAGTAATCGCAAGTAACGGACAGTAACTCTCGCACAATTAC
TACTTGAAACCGACGGAATCAATTCCGGTAACTGCTCACTTAATGCCTTCATCAATCCA
TGCACTTAGCATTTAATATGGGCCTTAGAATTTATTTGATTTAATTGTTGAATTAAAAATG
GACCAAGCTCAAATTAAATCCAACAAAGGTGACGGGCGCCAGGAACACTATCGAGGT
CGGCGTCTTCACCGGCTACTCTGTCCTCGCCACCGCGCTCGCCATCCCCGACGACGGC
AAGATCGTCGCCATCGACGTCAGCAGGGAGTACTTCGACCTCGGCCTGCCCGTCATCG
AGAAGGCCGGCGTCGCGCACAAGGTCGACTGTGACGACCCGCATTATTACCATTGGGT
AATAACGCCAGTCAACAGATTATTATTCGTTTTAGCTGATAAAACATCGCTCTAACGAAT
AATAAACCTATCAGAGTGTTGCAGCAGAACAATAATTAGAGACACAATATGATATTACA
ATAATATCGCCTCGCAGGGCTACATTCGATTACAATGCCTCAAGGGCTTAAATATTACAA
TCTTTAGACAAAATATTCTAACTATTTCCTATTCAAACAACTCCACACGGAGTGTCCACC
TGATACCCACTGTGTTCCGTGTCTACGGTATCCCATCCAGGTAACTTCCTCCTGATCTTA
GCCATCCCCTGATCTGACATGCTCCTAAGCAAAGGGGGAGATAATAGCAAGAGTGAGT
ACAGAAGTACTCAGCAAGCATTAATAGCAAAAATGCACAGAATGCTTATCATTTGCACT
TTTAGGAACATCAATTCCTCTATTCCATCTCATGCTCATTCATAACATTCTTTTTCATCAT
CAATTCCCTTTCCAGGACTTGTAGAAAATACAGATTGTTGGTTTTGAAAGAATTCCAAT
TTATTAATTTAGAAAACTACTGAACATACGCATGAACAGCAAAAACTCCCATAAGTTTC
CGTAATTCTCCTTTCCCGGAGACAGGCATGAATTCATGATTTTCAATAGCATTGCGCAA
GCTTGTATCACTTTACCCATCACCACTCTTGATCCTGGTGGCTCATGGTTCTCCGCAATT
CCTTAGATGCGGCGCCCAGATCAGAGCACAGTACGTTTGCATCTATCTTAGTAATTATAT
CTCCACCCAGTTACTCACAATCGGTAGCCTGGAGCGGTGTACCACCCTCACGCAGACC
CACCGAAAGCGACATTAACTGTTGGCTGGGGCGGTTCCCGACCATCTCCCAACACTCC
ACGGCGTAATATATCGCCCATCGCTTACCCGTTGCTGATAACGAACCAGTCTTGGGTTCT
TGCGATCCGCCTCACCCGTTGCTGATAACGAACCAGTCTTGGGTTGCGCGAGGCTGTG
GATAGCCCACCTTGCTGATAACGAACCAGTCTTGTGGGTTCCACTCCCTAACTAATACT
TACCCATGTGGAGAATATAATACTTTGATAGAGCTACTAGTCTGTCCGTCCTCAAACGG
AATGTTGGTTGATACGGTTTGGTACGGTCGGTTCACACGGATGAAGTTGGTAATCCCTT
ATATTCTATTCCTATGCAGAGGTTCCACTCCTGCCCCTTTATCCCTTGCCCTGGCAACCT
CTGCCCACCCTCTTAATTAACCTAGATGGCCAACTGCTCCATCAGTTGCCATCTATTTCT
TTTCTCATATCTCTTCTGTCCACTGCTCGAATTAAATATCTTTTATTCCTTTCATGCAGGG
TGAGATTAGAGTAATACAAAATACTTGTTTTTACAAAAGATACTCTGAAGCTTTGATCA
AAGGAATGCTTGCCTGGACTTTAACCATATACTCGGATTCAAGTAGGATCAGGTGAAGT

TGTCCCCGTAGCTTGGATTGTGGCCTGCAATTAAATACGTTGGCTACGACTTAATTAGC
GGTGCATTAATTAAATCGGCATACGTTATGCTTTTACTCTATCGCTGGTAAATTATTTAAT
CTAATCATTATAAATACTGATTAGCGGATTTCTATTGCTTTGATGATTAAACCAATTTATCT
GCGTATTCAAGTCTGCAACTAAAAGGGTCTTAAACTATAGTACTTTAATTAAATCCTTAG
GCGTACAGGTTTAATCGACGTATACTTTTCATCGTTGGACCACAATTAGTTTGTTCTAAT
ATTATGAAGAACTCAGGGTGAATTTCTATAGTTTAACTATTATTACCCAAACCGTCTAAG
TAATGGAATATATTGATGAGCGGTTTAAATAACAATACACAGGTTAAGAGTATACACATG
GATCCATTCTAATCAATTGACGTTTTGGTTTATTTATGGGATCCTAAATATTTATTAATAGA
ATTCCACAAGTTTATACTACCAACAATGTTAAATAGATATTACTTAGGAATTTTAATAATT
TGCACGGCAATGTATCGATTCAATCGGTACTCGCCAAATTTTAATCTACTGCCTATCCTT
AATTAATCATGACTACTGATTTGAGTTAATTTGTATTTTAGCATGATTAGAATAATAATAA
TAGGTTTTTTTTAATCTACACTAGCTTAGTTAAAGTTAAATATTCACAAGACTGATTTTTA
TTATGCGCGCTTCTCCTCTCTCTTCTCTCTCTTTTTTTTTCTCTTTCTTCCGCCCGACCCC
TCCTTTTTCCTCTGCCGACGCCTCTCCATCCCCCCCTCCTTTTCTTCCTTTCTCGCTCGG
CCTCTCTGCTCTTCTCCTCACGCCGCCTCTCCCTCGCCTCCGTGCCTCAGCGCCGCCGC
CCGGTTTGGCTCCCCTCACGCGCCGCCTCTCTCGCGCCCGCGCCTCGGCGCCTCTGCC
TCGGTGCCGCTCCCCCTCACGCCGGCTCCCCGCCGACGCCTCCTCTGGCCGGCCGTTC
CCGCCGAGATGACCGGCGCCGCTTGGGAATCTCCGGCCAGATCTGGCCCACTCGGATG
CTCCTCCTCCTTTTTCCCCATTTCTTTGCGTGATTTACTTTTGTGATTTGCTAACGTGTGG
GGTTTTTGCCTAGGGTGTGGTATTGGGAGGCGGGTGACCGTGGCCGTCCTCGCCGGTG
AGCCGTCGCCATGCGCCGGGGGAGTACTGGCGGCGGTGAACCGGTGGCTCGGCCGTG
ACTCCCGCAGCCGCCGCCCTCCAATCCTTCCCTTCCCTTTGTCCCCCCCCCCCCCGCAC
GAGAGTAGTGGGTAACCCCTCTTCTCTCCTTCATGCTACTTCCGGTGATATGCTTATGGT
TCATGTGTTCTATTGTGGATGAATGCTTATGGTTGCTACTTGCTAGCCTATGTTGGATGG
AATGGAATAATGGCATTGCATAGATGTTGGGCTTTTGGGGTTTACCTGCACTGGTGCCT
GTGCGGGATGCTAGGGTTTGGGGTTGGGGTGGTGGTCGATTGACGGTGGCTGAGGCTT
GCTGCTGGCTTGCGGTTCTCCTGTGGCCGTCACGTCCCCATCTTCCTTCGGCTTGTCGG
TTCTTCACTCCCTGGCCTTCGGCTCCACTTGGTGACGATGCCTCTGCGGTCTGGGGATT
GGCTGGAAGAATTTGGGGAATGTTACCTTACCTTGCTTTGTCCCTCTGTGCTCTTTCCT
CTCGGGTTGGCTACTGGTATGCAGTGTGGGATATTGTGGTGGATGGCTTGCCCGTGAGG
CTTTGTTGGCTGCCGATACTCCCTGGGCGTGGTTGCTCTGGCCCGGTGCTATCTGATTC
ACTGTGATGGATATTTGCATGGTGAGGGCTTTGACAGGTGTTTTCTCTGTTGTAGGTTT
GGCTGGGATGATTTGTGCTCACCTAGCCTTTGCCTTGGTTGGATTCCTATATTCAAAACA
GGTTTGTATATATATATATACCGTGTGATGAGCCTATGGTTGTGAGAGGTGCCGACTGGC
CCCCGGACGGTCAGGTATCTCTGTGAACGGATTACCCCGCCCGAAGTTAATCCTTAGGG
CTCTGACGGCAACACTTCTTTTATATTTCCTTATTTTCAATTACTCTTTTGTTTGGTCTTT
TTCGGTATTATAATTTGATTGTTTAATCACAAATGAATAACGAGGTAACGGCAACTCGAC
GATAGTCTGATCCATGTATGAAATGGCCTAAATTGTAATCGGGCTTTCTCTTAATATTGAT
GTATACAAAATATTGTATGTTACTCGAATGTAGTTTCTAATGTTGCAATTGTGTTGCAATT
ATCTAAGTTTGCGAGACGGTCATTTGATTAAGACTGAGACTTGTAAACTTACAGTAATT
TGTATTGTCGTTATTAATGTAAAAAGAGTTGTAATATCTTTATTTAATTTAGGCCAGTTTT
AGTTGTAGCTCAAGGGATGTGACTTGATTTGTTGTCCAAGAATTGTAATAATTACACAA
TTGTATTACGGACTGTTATATATATTTTAATTGACTTGAAAGTTTTTATTTGTCGTGCAAG
TATATTTTATCTAGCTCTAGTCTTTACAATTTGGTCTGCGTCCTCATCGACGGAATTACGG

TAGTAACGTATTCGTTTATCGTTCGTTGGTTTCAATGTATACGGTTACGTAGAAAAAGTT
TAAATTTCCTCGTATTTTTTTAGATTATCATTTATGTAATTAATCCTACTTAACGTCCTAA
TTCGCTAATTATGGTTAATTAACGACGTGGGTCGTTACACACTTCCCTCCTTAAAAATGA
TTTCGTCCTCGAAATCGGAAATGGCTTAAGAAGGTTGTAGTAGGTCTCTTATTGATCATT
TACGAAACGTGATTTTAGATATTCCTATTTATTCCGCCTTTTTGTAGATACACTTCCTATG
CTTGTATCGCTCGTTGACTCCTCACATGTCATCGCTGAAATTCGATATCAAGCTATAGGT
TATCGGTTATCGTTACTAGGGATTTGGTAGCATTAGGATTGGAATTATAATTCATGTTAAG
GTAGGAAAATCGTTCAATTTATTCTATGTTGCACCGGTTAGTATTCTCCTTTTTCTACCCA
TTGCCCCTTCCCTCCTTAATTCTTTAGCTACCCCTCGATTCCTTTAGCTGGTCTACTGAA
GTGCGCTAGTTGATAGTTTAGTAGTGTGGTTCAAAGAAAGCGGTGTTGCTTAAAGCAAT
TGGGTTGAAAAGAAATCTCATGGTAGGAAGATTTAGGATTAGATTAGTATTATCCTAGTT
TCCTGGCATTGTACTTTGGCTATTTAAGGGTTGGGAAATATCGTTACTTCCTATTCGGAT
GGCATAATGGTGTCTGAAATTTTGGATTGAAACTCTGTCTACGGGACTAGCTCTTTAGC
CGTGGTTTAGCTCTTCGGAATTGAACAGCTCTCCCTAGACTAATAGGCAGGCTGTGAG
ATTTATGGGTTACACAGAGGGGTATGGTATTGCCATACGATCTTGAGTTCAGTTGATGAT
AGATCAATGATATTGAAAGAGTGGACATTTCCCCTTATTCATAATATATGTCCTCATGGA
ATGCATGAAATAAAGTTTTCCAAAAGCTTTAAACACTATGGTGCAAGAAATATTTATATA
GAATGATGCAAAGAGGTTTTGTGAAAATACTTATTTCCTAATGTCATGTTGAAACATTTT
CGTTTGCAGAATTTCATTTTATGAGCTCATGGTCAATGTCATGCCCCGGGAATTATGCCA
AAAGTTCCTGAAATCATATATTTATGAACTTGGGGAAATGCACCAGACATAGACAATAA
TATTTCAGTGGATGCTTTAAAACAGATCATGGATGCCGAGGTGATTAACACCGCAACAG
GCTTAGACTTAGAAGCCTAGGGGTTGACGGTTCAGGTCTGAATTAAACAAAGAGACAC
AGGTAGCTATGAACTCATTCCCTCGGTTTGACACCAGAGTGGCTACGTGTCTAACTATA
CACTCAAAAAGAGCTAGGACAAAATAAAGGAATGTTTCTTTGGGAAGTTTAAGGATAT
ACTACTCGGGAAAAGACTCGAATAACCCCCATTACGATTGGGCTATCTATGATCCAAAA
GATGAAGAATAGGTAGCCTAATTTAATCCGGTTACGCTCTAGGGACGTCTCACCCTAAG
TTACGTGCTCCGTAAAAGAAAATGGATAGTGGCGCATATATTATCTACAGTCGGTCTCGT
AACAACTCGTCTAAGTTTAGTAGTCCAGGTAATTCTATATAACCGGCTTTTAGAAATAAT
AAGGAATTCGAACATTTTCCTATATTACGGACTAGGCTCTAGATTTTTTTTTTCACTGGT
TTTAATGTGATGGTTCCGTATACACTTAGTCACCTCATTTTAAGTTGTAGTTAAATTTAGG
GAATTATATTTTCGAAAATACCTGCATATCATGACGTTATTAAATTTCCTGCAGCAAGTT
GTTACAAGAGGTACGAACAAAATTTTATTCATGTTGTAGACCCAGGTTTTCCTTATTAGT
ATTCAGCATGCAAATAGATGAGAATTTATTTATGTTCAAGTAGTAGAAAGCCGCATTACT
CCCTCTCGGTTATTGGTCTTCACATCACAGCATACACATTAGGCAAATAAAACCCAAGA
AACATAGAGATGCAAAAAGAATCAACTCCTACACTACTCTGGGACTACGCGCCCAGCT
ACGCCCGAGAACTCGCTCTCGAGACCGCCCAAATCTTTCGCGTTTGGGACCACGCTCA
GAGCTTGCCTCTGAGTGCGAACCTACACTACCCTGGATCTCCGCGCCCAGGCACGCCT
AAGAACTCGCTCTCAGGATCGCCCAAAACTTTCGCGTTTGGAACCACGCTCAGGGCTC
ATCCCTGAGTACGCCCAGGACTTCGTTCCTGGGTACGCCCGAAACTTACGCGTTCGGG
AGCGCCACGGACCACTCGTCCGGGAAATCTCATATGCAGAAATGGGGTAAAGCAATTT
TTTTTAGTGGACCATACATCTTGCCATCTTAGTACCTTTGCTCTAAAATTCATACTCTCCT
TGCTTAGCTGTCTAAGATGGCAAGGTGTTTAATTTAGTTCTCAGATTGAAAGAGATAAG
AAGTTATGATAGGATGGTCCTCTCTACATCACTTTTACTTGCTAACACTCAAGTTTTCTA
TCCTAGGTTGCAAAATGAAAGTTCTGGACAGCAACATGCATGTAACAAAAATGAATTG

AGTACAGGAAGTAAAAATAGGTAATTGCTATAATTTCGATCATTCTACACCTCCTATTCA
TATTACTTACAACCCCAGGTATATACTAGCATGCATGGCCAAGATGATTATTCCTAGCATA
ATACTACCATTACATTTATGTTTCTCAAGTTAGCAAACATTAAATTTGTAATTTTCAGCCC
ACTACTACCCAAAATTTATACTTTTAGATATGACAAAATGAAACTACCTCAATCCTGCTC
ATATTTGTCTTCGATTGCCCTATGTCGCTATGGTTAAGAAAATACAACAATCATACATTAA
CTGAAATGCAATAAGTCAAATGCGGAATACCTAATTGCAGCAAAATTAAATGTTAAATT
CGTAATTAACATTATATTGCTTACCAACTAATACTACAGTTTTGTAAACAAACACTGTTA
CCGATGAAAAGCAACTAGTAACATAATTTAAATTGTCATAACTATTATTACCACCGTTCC
CATCCTCACTACCCATTCTGGCTCTAAAAATATGCGATGATTTAAAAGTAACGATGCTTA
AACCTAGAGCTAACCACATAAGAAAGGATGTACAGATGAGATGTATTAAAGGAGATGT
ATTGCCATATAGCAAGGATCACCTCCTTAAGTCAAGAAACAGTCTCTAATGATTTGCGT
GGCCCTCAAACCATTGCTCTGATACCACCTGTGACGACCCGCATTATTACCATTGGGTA
ATAACGCCAGTCAACAGATTATTATTCGTTTTAGCTGATAAAACATCGCTCTAACGAATA
ATAAACCTATCAGAGTGTTGCAGCAGAACAATAATTAGAGACACAATATGATATTACAA
TAATATCGCCTCGCAGGGCTACATTCGATTACAATGCCTCAAGGGCTTAAATATTACAAT
CTTTAGACAAAATATTCTAACTATTTCCTATTCAAACAACTCCACACGGAGTGTCCACCT
GATACCCACTGTGTTCCGTGTCTACGGTATCCCATCCAGGTAACTTCCTCCTGATCTTAG
CCATCCCCTGATCTGACATGCTCCTAAGCAAAGGGGGAGATAATAGCAAGAGTGAGTA
CAGAAGTACTCAGCAAGCGTTAATAGCAAAAATGCACAGAATGCTTATCATTTGCACTT
TTAGGAACATCAATTCCTCTATTCCATCTCATGCTCATTCATAACATTCTTTTTCATCATC
AATTCCCTTTCCAGGACTTGTAGAAAATACAGATTGTTGGTTTTGAAAGAATTCCAATT
TATTAATTTAGAAAACTACTGAACATACGCATGAACAGCAAAAACTCCCATAAGTTTCC
GTAATTCTCCTTTCCCGGAGACAGGCATGAATTCATGATTTTCAATAGCATTGCGCAAG
CTTGTATCACTTTACCCATCACCACTCTTGATCCTGGTGGCTCATGGTTCTCCGCAATTC
CTTAGATGCGGCGCCCAGATCAGAGCACAGTACGTTTGCATCTATCTTAGTAATTATATC
TCCACCCAGTTACTCACAATCGGTAGCCTGGAGCGGTGTACCACCCTCACGCAGACGT
ATACTTTTCATCGTCGGACCACAATTAGTTTGTTCTAATATTATGAAGAACTCAGGGTGA
ATTTCTATAGTTTAACTATTATTACCCAAACCGTCTAAGTAATGGAATATATTGATGAGCG
GTTTAAATAACAATACACAGGTTAAGAGTATACACATGGATCCATTCTAATCAATTGACG
TTTTGGTTTATTTATGGGATCCTAAATATTTATTAATAGAATTCCACAAGTTTATACTACCA
ACAATGTTAAATAGATATTACTTAGGAATTTTAATAATTTGCACGGCAATGTATCGATTCA
ATCGGTACTCGCCAAATTTTAATCTACTGCCTATCCTTAATTAATCATGACTACTGATTTG
AGTTAATTTGTATTTTAGCATGATTAGAATAATAATAATAGGTTTTTTTAATCTACACTA
GCTTAGTTAAAGTTAAATATTCACAAGACTGATTTTTATTATGCGCGCTTCTCCTCTCTCT
TCTCTCTCTTTTTTTTTCTCTTTCTTCCGCCCGACCCCTCCTTTTTCCTCTGCCGACGCCT
CTCCATCCCCCCCTCCTTTTCTTCCTTTCTCGCTCGGCCTCTCTGCTCTTCTCCTCACGC
CGCCTCTCCCTCGCCTCCGTGCCTCAGCGCCGCCGCCCGGTTTGGCTCCCCTCACGCG
CCGCCTCTCTCTCGCGCCCGCGCCTCGGCGCCTCTGCCTCGGTGCCGCTCCCCCTCAC
GCCGGCTCCCCGCCGACGCCTCCTCTGGCCGGCCGTTCCCGCCGAGATGACCGGCGCC
GCTTGGGAATCTCCGGCCAGATCTGGCCCACTCGGATGCTCCTCCTCCTTTTTCCCCAT
TTCTTTGCGTGATTTACATTTGTGATTTGCTAACGTGTGGGGTTTTTGCCTAGGGTGTGG
TATTGGGAGGCGGGTGACCGTGGCCGTCCTCGCCGGTGAGCCGTCGCCATGCGCCGGG
GGAGTACTGGCGGCGGTGAACCGGTGGCTCGGCCGTGACTCCCGCAGCCGCCGCCCT
CACGGTAACCCTCTTCTCTCCTCTGCTACTTCCGGTGATATGCTTATGGTTCATGTGTTC

TATTGTGGATGAATGCTTATGGTTGCTACTTGCTAGCCTATGTTGGATGGAATGGAATAA
TGGCATTGCATAGATGTTGGGCTTTTGGGGTTTACCTGCACTGGTGCCTGTGCGGGATG
CTAGGGTTTGGGGTTGGGGTGGTGGTCGATTGACGGTGGCTGAGGCTTGCTGCTGGCT
TGCGGTTCTCCTGTGGCCGTCACGTCCCCATCTTCCTTCGGCTTGTCGGTTCTTCACTC
CCTGGCCTTCGGCTCCACTTGGTGACGATGCCTCTGCGGTCTGGGGATTGGCTGGAAG
AATTTGGGGAATGTTACCTTACCTTGCTTTGTCCCTCTGTGCTCTTTCCTCTCGGGTTGG
CTACTGGTATGCAGTGTGGGATATTGTGGTGGATGGCTTGCCCGTGAGGCTTTGTTGGC
TGCCGATACTCCCTGGGCGTGGTTGCTCTGGCCCGGTGCTATCTGATTCACTGTGATGG
ATATTTGCATGGTGAGGGCTTTGACAGGTGTTTTCTCTGTTGTAGGTTTGGCTGGGATG
ATTTGTGCTCACCTAGCCTTTGCCTTGGTTGGATTCCTATATTCAAAACAGGTTTGTATAT
ATATATATATACCGTGTGATGAGCCTATGGTTGTGAGAGGTGCCGACTGGCCCCCGGAC
GGTCAGGTATCTCTGTGAACGGATTACCCCGCCCGAAGTTAATCCTTAGGGCTCTGACG
GCAACACTTCTTTTATATTTCCTTATTTTCAATTACTCTTTTGTTTGGTCTTTTTCGGTATT
ATAATTTGATTGTTTAATCACAAATGAATAACGAGGTAACGGCAACTCGACGATAGTCT
GATCCATGTATGAAATGGCCTAAATTGTAATCGGGCTTTCTCTTAATATTGATGTATACAG
AATATTGTATGTTACTCGAATGTAGTTTCTAATGTTGCAATTGTGTTGCAATTATCTAAGT
TTGCGAGACGGTCATTTGATTAAGACTGAGACTTGTAAACTTACAGTAATTTGTATTGT
CGTTATTAATGTAAAAAGAGTTGTAATATCTTTATTTAATTTAGGCCAGTTTTAGTTGTAG
CTCAAGGGATGTGACTTGATTTGTTGTCCAAGAATTGTAATAATTACACAATTGTATTAC
GGACTGTTATATATATTTTAATTGACTTGAAAGTTTTTATTTGTCGTGCAAGTATATTTTAT
CTAGCTCTAGTCTTTACAATTTGGTCTGCGTCCTCATCGACGGAATTACGGTAGTAACGT
ATTCGTTTATCGTTCGTTGGTTTCAATGTATACGGTTACGTAGAAAAAGTTTAAATTTCC
TCGTATTTTTTTAGATTATCATTTATGTAATTAATCCTACTTAACGTCCTAATTCGCTAATT
ATGGTTAATTAACGACGTGGGTCGTTACATCGACTTCCGCGAGGGCCCCGCTCAGCCC
ATCCTCGACAAGCTGCTCTCCGAAGTACGTACGGGATCAGCCACCGCCACCGGGTCTT
TCTTGATGACCCATGCATTTACCCAATGCCGCCGGCCTGTGGCTTCAACGGTCGCCATA
ATTACTTGCTCATCGATGTGATCTTGGTGCGTGCGTGCAGGAGGGTAACGAGGGGAAG
TTCGGCTTCGCCTTCGTGGACGCCGACAAGGGGAACTACGGCAACTACCACGAGCAG
CTGCTGCGGTTGGTGCGAGTCGGCGGCGTGCTCGCCTACGACAACACGCTCTGGGGC
GGCACCGTGGCACTGCCGGACGACACGCCGCTCACGGAGGACGACCGGGAGATCCGG
GACTTCATCAGGGCGTTCAACGCCAAGATCGCCGCCGACCCGCGCGTCGAGGCCGTG
CAGCTCCCGGTCGCTGACGGCATCACGCTGTGCCGCCGCGTCGCC**TGA**

>DfCCoAOMT14 (DfaA11G016070)
TTTTGGATGGCCTGAAATAATAGCGCTTGGAATTAGAAGTTTGGTGTTTGATTTGGCTTT
TAGTTAGACTGGCTGCCTGGGCTGATTCGGCTTTTAGTTAGATGGCCGCCTCATTGCCA
CCTATTCCGTCATCTTCGATTAAGTTGTCGCAAAATCTTTCGTATCACTTTTTGTTCCAG
ATTCACCTTTTACTTTTCTCTTGATGAATTTGATCTCCCCCACGGTTCATCACCGGATTT
GGGTCGTCTTCCTAGTCGCCATCGTCAAATTCTTCGCACTCGGTGAGCATCTCTGCAAT
CCTTTGCACCTGTAGCATGAAATTTATCCGCTCTACCTTTTTCCTACTTCAATTTGGTTG
ATGTACTGCCATGGGGTGCTGCCACCACCACCATCTTGTTGAGCTCCAACTACCGCCCA
ATCTCCATCTCTGGTGAGCTTGCGTCGAACCCAACTGCATCATGACGTGTTCCACCTTC
GTACCAATTATCATCCTCAGTTAATTGCATTCTCTCACAGTCATTCGCCGGAATCAAGCC
ATCTTCCTCGTCATCGCCCAAACCGTCACCGCCCTAGATTTCATCGCCGCTGGTGAGCT

ACTGCCATCATCAACCTCAACATCGTCTTTCTCATACCGCGTTCCACACCATGCTCACCT
CGCCTATATTGGAACCCTTAGGGGTGAAAACGGGTCGGATTCGGACGGATATTGCTGTT
ACCATATCCTAACCCATATTTTTCTCCGGATTCGGTACCAATCGGATTCGGTACCAATC
GGATTCGGTGTCGGATCGGTCGGGATTAACCCATTTCGTTTGCACCCCTACTTGTTTGTA
AGGAATGCATACAGTGCAGTAAATGGAGTAGGACGCACTGACTTGATGTTACTATTTAA
TAGCTGATGTGGAAGATTTTATTTTACCATTGCAAAAGAAAATGAAACGTCAAATAGTA
GCACTAACCTGAAATCAAACTTGAAGATGTATCAGATTCGGACGGATATTCGGATTCGG
ACGGATATTGCTGTTACCATATCCTAACCCATATTTTTCTCCGGATTCGGTACTAGTCGG
ATTCGGTACCAATCGGATTCGGTGCCGGATCGGTCGGGATTAACCCGACCCGTTTTCAC
CCCTAGGAACCCTGCCGCCATCCGTCTTCACCCTGGCTGCCGACCCTACTCGTCGCCA
GCGTCACCGTCGTGTAGGACCACTGCGACGCCAACAACTTGTTATGTTCATTGGCCATG
CCTTCTAGGCCGTCACTCGTCGAAGCCAATGCCAAAGCGGCTTCGAGAAGCCAACTGT
GCAAAGCTACTTCTCACAAGAATATATAGTTTGATTGTTAGAGACTGGAAGCCGAAGCC
GTTTCAGGAACGCGGCCTAGTTAGGCGGTGCACCCTGCGAGAGTCCCTCAACGAAAG
AGTGATGCACGCACGGAAGCAGCCAAGCTGGCGCAGGGTAGGTGGGCGGGGCCCAG
CGTGGGCTCTACGGTGGGCCCCCCTCCCTCCATTCACCCTCCCGACGACCGTCCCGG
TCCATTCCCCACCAGTCCGTTTCCCACCAACCCTCCCCGCTCGCCCCGCGCGAGAGAC
CAACCCGCTCGCCGTATAGACGCGGTCCACGCAGCGAAGCCAGAGAGGCAGGAGGAG
AGAAAAGTCTTTGCACCAATCGCAGTCCACCACCAACCCACCCTCCCCTCCCCTCGGA
ATCGCTCACCAATCGTGCCATGTCGCGGCCAACAACGAGCGACACCAGCTCACCAACC
CAAAGCTGTATTCTACCGACCGGTACGGGTCACTGACATGTGGGCCCCACAGGCTACT
GTACCGCTCTCTCCTCTTGGGGTATTTTTGTGCTATTTTTTTCTTTTTTTTTTCCATTCTG
GTAGTCGTCACGTCCAGTGTACCTGCCTTCCTACGCACAAGCCCACATGCCTCTCCCGA
TCAGTCCACTGCGCCGCGCTATATAAACCGCCCGTTTGCTTCCTCATCCCGGCAAGGTC
AATCGAGCGAGCCGCGCATTAGTATTACTACACCCGGATCTCGTCTCCTCGAAGACCGG
CA**ATG**GCCACCGCGACCGCCGATGCGACGACGGCGACCAAGGAGCAAACCAGCGGC
GGCGGCGGCGGCGGCGAGCAGAAGACGCGCCACTCCGAGGTCGGGCACAAGAGCCT
GCTCCAGAGCGACGCGCTCTACCAGTACATCCTGGAGACGAGCGTGTACCCGCGCGAG
CACGAGTGCATGAAGGAGCTCCGCGAGGTCACCGCCAAGCACCCATGGTATGGTGCTT
GTCCTCCTCCTCTCCTCTTCTTGATCTGAATCCGCGAGGTCACCGCCATGCGCGCGGCT
TTAGTTAATGTCGGATCTTGCTTGACCTTGTGCCTGCAGGAACCTGATGACGACGTCGG
CGGACGAGGGGCAGTTCCTGAACATGCTGCTCAAGCTCATCGGCGCCAAGAAGACCA
TGGAGATCGGCGTCTACACCGGCTACTCCCTCCTCGCCACCGCGCTCGCCATCCCCGA
GGACGGCACGGTAAGTCCATGGATCGGCTAGCTACTACTTCCACGCGCGCCGCAGATC
CCTAGATCTGGCGAGCTGAAACGATCGATGGCACTCGATATCTGCAGATCTTGGCCATG
GACATCAACCGCGAGAACTACGAGCTCGGCCTGCCCTGCATCGAGAAGGCCGGCGTC
GCCCACAAGATCGACTTCCGCGAGGGCCCCGCACTCCCCGTCCTCGACCAGCTCCTCG
AGGACGAGGCCAACCACGGCTCGTTCGACTTCGTCTTCGTCGACGCCGACAAGGACA
ACTACCTCAACTACCACGACCGCCTCATGAAGCTGGTCAAGGTCGGCGGCCTCGTCGG
CTACGACAACACGCTCTGGAACGGCTCCGTCGTGCTCCCCGCCGACGCGCCCATGCGC
AAGTACATCCGCTACTACCGCGACTTCGTGCTCGAGCTCAACAAGGCCCTCGCCGCCG
ACGAGCGCGTCGAGATCTGCCAGCTCCCCGTCGGCGACGGCATCACCCTCTGCCGCCG
CGCCAAG**TGA**ACCGTCCCTCCGGCCATCTTGGTCGCTCGACGGCGCGCGCGCCACCAT
GATGCCATCGTCTGCTCCTTCTGCCATAGATGCGAATTGAAGAAGGATTAATTATCGTTT

TGGCTTTCTTGGCTTTTGTATCTTTTTTTGATATCACCATTTCATACGTAAAAAAATGTCG
AGGCAATAATATTATAAGATGACCCGGTGTTTGTCA

>DfCCoAOMT15 (DfaB11G015250)
ACCTGGCTGCCCGCAACGCGCGGAGAAAGCAGGGTTGGTGGGCGGGCCCCGACCGCC
GCTGCCTCTTCACCTTCCCGATGACCGTCCTGGAAGGAAGCCACCACCGATCCGTCAC
CCATCCCTCTCCCCCCTCACTCGCGGCGCCTCTCCCCATCTGCTAGCGGGGAGGCCACC
TGCGCAGCTGGCGCACGGGCGCGCTCCACGCACCGAAGCCAATCGCAGACTCGCAGT
CCACCACCCACCCCCACCTTCTTGCTCTCCTATATCTCGCTCATTTCGATTTAAACTTTT
AACGTCTACCTTATTTTTTTCACTATTTCTAAATTTTATCTTTTTCTAGCTTTTCTGCTTTT
TTTGAGCTTTTTTGCCGGTTATTTTTTTGTATTCGTTTGCATTACACGTGATGGGCTAAC
ATATTTTTTCTATTTTAAAATTTTTAAAATTTAATTATGCCAAATAATCCACCATCAATTAC
AATGTAATTTTAGTTAGCTACAGTGCAATTTCAACCATAAACCACCAAATTTCAGCTCA
AATGAGCGGGCTGCTAATCGCTCACCAATCGTGTCCATCTTGTCGCGGCCGATAAATAA
AGGGATAAGTCTACTTCTACCCACCGAACTCTTGTGCCATCTAACCTTCAACCCAAAAA
ATATATAATCAAATATTTTGTGGTCTTCAACTATCAATACCATTTATTTTATCTCTTAAAAC
TAATTAAAGTGATTTTAACTGACATGACATCAACATGGTAATATTTTTGACACATGAGTT
GGTTTTGAATGACATATCAACCATTCATCAAAATCATCAAATAAATCAAAAACATATGGT
GAACAGGAGGCTAGATCTAGGGGAGGAGGCCAAAGAATTATCATGGGTGAGCTCACCT
TTTTCTTCCGCTGTGACCTCACCTCCCAATCTCTCTCCTCTTCCTTCGTCCGTAGATCAT
CACCGTCTTCTTCTTTATTATCCTAGAGGTTAGGACGATGTTGGGCGCGATGGGAGAGG
GGGTCACAGAGGAGATGGATGCGTGGTGGAGGGCAACGAGGAGGGGGAGCTAGTGC
AGGCCTCCTCCCTAGATTCAGCTATCGCTCTTAGCCTCCTCCCCAATTTCAAGTTGCGG
CAACGGCAGCATTTAGCTGAAGCTACTTTGCTTTAATCCCTCACAATAAGGCCTAGATC
TTGTGTGCCCTGGTCGCAGGGGCTGCCTCCTCTGTCCACTTCAATCACCAATAGCAACA
TCTTGCCGCCCGCTCTAGTGAAAACGTAGGCGCAAAGGTCAGCGATGGTGTACCTTCA
TCCACGTCATGATCTCCGCCTCCAACGTCGATTAGATCATCTGCCGTGGTGATGAACTC
CAGGGGAGAGGGAGAGAAGGGAAGAGAGAAGAAACATGGTGATAGTGGTTGTTGCA
AGCAGCTAGTGGCGTGGCCAATTGCGATATAGAGAAGAGAGGAAGAGATGAAATGAC
ATGAGGTTGATAGGTGGGTCCCACTGTATATTTCTTATTTATATGATAATTATGCTGACTG
AGTTGCTATGTCACCCTAAAACAAACTCATGTACCAAAAACTATTTCCATGTCAACGTC
ACTAAGACAAAAATTGAGATGGAAAGGTCTCAAAAGCAAAATATCCGGTTTTGATAGT
TATGCGATATTGAATATCCGATTTTATAGTTTAAGGTTGAAACTTAGACTATTTTAAAAAT
TTAGGTGTACAATAGACTTATTTCTAAAATAATCTAGTACTGGTACCACTAGCATGTGTA
TTCTACAGACCAGTCTTGAGTATCTGACACGCCGGGCCCAACGGCTGCTACAGACGAC
TGTTCCACGTTCTCTCCTACGCGCAAGCCGACATGCCTCAGGCGACCAATCCACCGCG
CCGCGTTATATAAAGCCGCCCGTTCTGGTCTCATCTCGGCAAGGTCAATCGAGCGAGCG
AGAGCGCACCAGCACAGCCCAGATCTCGTTTCCTCTTCTAAGATCAGCAGCA**ATG**GCC
ACCGCGACAGCCGAGGCGGCGACCGCGTCCAAGGAGCAGACCAACGGCAGCGGCGG
CGAGCAGAAGACGCGCCACTCCGAGGTCGGGCACAAGAGCCTGCTCCAGAGCGACG
CGCTCTACCAGTACATCCTGGAGACGAGCGTGTACCCGCGCGAGCACGAGTGCATGAA
GGAGCTCCGCGAGGTCACCGCCAAGCATCCATGGTATGCGCGCTGCCATCCTCCTCCTC
CTCGTCAGTCTTGATCTGAATCTGGTCTGCGAGATCGATGAGACCAATCGCTACAGTGC
GGCATGTGGCGCCGCATTACATTTGGCCCCGGTTAATTTCGTCGGATCTTGCCTGATCTC

GTGGCTGCAGGAACCTGATGACGACGTCGGCGGACGAGGGGCAGTTCCTGAACATGC
TGCTCAAGCTCATCGGCGCCAAGAAGACCATGGAAATCGGCGTCTACACTGGCTACTC
CCTTCTCGCCACCGCGCTCGCCATCCCCGAGGACGGCACGGTAAGTCCATCGATCAGC
TAGCTACTACTACTTCCGCGCGATCCGCAGATCCCCAGATCTGGCGAACTGAAATGATC
GATGGCACCGATGATGCAGATCTTGGCCATGGACATCAACCGCGAGAACTACGAGCTC
GGCCTGCCCTGCATCGAGAAGGCTGGCGTCGCCCACAAGATCAACTTCCGCGAGGGG
CCCGCCCTCCCCGTCCTCGACCAGCTCCTCGAGGACGAGAGCAACCACGGCTCGTTCG
ACTTCGTCTTCGTGGACGCCGACAAGGACAACTACCTCAACTACCACGAGCGCCTGAT
GAAGCTGGTTAAGGTCGGCGGCCTCGTCGGCTACGACAACACGCTGTGGAACGGCTC
CGTAGTGCTCCCCGCCGACGCGCCCATGCGCAAGTACATCCGCTACTACCGCGACTTC
GTCCTCGAGCTCAACAAGGCCCTCGCCGCCGACGAGCGCATCGAGATCTGCCAGCTCC
CCGTCGGCGACGGCATCACCCTCTGCCGCCGTGTCAAG**TGA**

>DfCCoAOMT16 (DfaC11G007040)
TACGCTTACTAGACTGCAGAGCAGGGGCGTAGCTGGGCCTCCGGCCGCCGGGGCCGC
AGCCCGAGACCCAGAGCAAAAAATCAACTATATAAATACTGTAGCAACAGTAAGATAG
ACTGCAAAATTTATTTCTAGTAGGCTAGGCCCGAGGCCCAGCCCACGGCTGGCTACAC
CCCTGCTCCAGAGGGATAGCTGCCGTGTCAGGTTGGTGAATTTGGATCCCGATCCAGA
AGATATTTTTCTTGGTCCAAACAAAGCTGGTCCTTTTGCATGGCACGATCATGTTTCTCC
TCATCCTTAATTTAACATCGCATATCCACAGTGGCGGAGCCAACTATTTGGCTTGGTTTG
GCAGCCGCCATACCTAAATCATATAAAAAAACGAATATATGTAGTATTTGTTCCAAGAAT
CTTCATTTAGTGAGGTATTTTAAGTACCAATGCTTTGGATTTCTGTTATATTTGCTCATTA
AAAGTAATATATATATATATATATATATATATATATATATATATATATATATATATATATATATA
TATATATATATATATATATATATATATTATATATATATATATAGATAATGTTCTGGTGCGTCTTTGCATA
GTTGATAGTTATATTATATATGTTTGACCAATTGACCTTGCAAACCCGCCACACCTGAAA
ATTATTCCTGCCTCCGCCACTGCATATCCAGGTACTTGAGCTAGGCAAACACCATAACG
GAGCAAACGCCACCTCATGCGACATACCAACAACCTCTACGGATCTATGCGTTACTAAA
TTGGTTGTTCACGAATTGTTAAGGTTTTTGGTCTTGCTTAAAAATGATGTGAATTTTGGT
TTTTAGTTGTATCCTTCGACTTTTGATTAATTGTGAAAGAAATTAAGAGGTAAGAAACA
CACGCATTAAGAAGCTCGCCTTATAAAAACTAACTTGAAAAGTCACTCCGTTCATAAAC
TGACTTCTAAAAAAACAAAATCTGTTTAGAACAAGGTCTTCATAGGAAAATGGACCAG
AAAGACAACGAAAAAAGGGAGTTTTACCGTATTACGGCAGCAAATCAACACAATATCC
AATTTGATCGTCAAACTTATCGAATTTCAGTTGAGCAAAACTTAGCGTGCGCATGTTTG
AAACGAGCCACCAAACTCCCTATCCACCTCCTCTCCAGAGCAGTGCCCCTAATTAATCG
CACTCGCTTTTCCGTTCGGGAGAGCGGAGTGTGTCTCCACCCAGCAACGAACGAACG
AACGATGCCACGCACGGGAAAGAAGGGTAGGTGAGCGGGCCCCGCGGCGGGCACCA
ATTCGGGGCTTCACGGTGGGCCCCACGACCCCCCTCTTCACCCACCCGACGACCGTCC
CGGGAAGCCACCACCACCCCACCCGTTCCTTTCCCACCAACCCTGGTCCCCACCCCCG
TCCACCAAACCCCCAACGTGGCCACCCGCGCAGCGTATAGACGCGATGGTTACCGGCC
GGCGGTGCACCACCCGGCGAAGCCAGAGGGGCCGCGGCCAAGAATAATAAACTACAC
CAGCTCACCAGCCCAAAGAGCAAAGCTGTCTGTATTCTGCCGACCAGCTCGAGTCCCT
GACACGTCGGGGCCACCGACTGCTGCACAGGACTGTTCCACGCTCTCTCTCCTGTCGG
TTAGTGGATTTTTTTGGGTAGATAGTTTAAATATTTGAATTCTTGTTACTGTGAGAGGTA
GTACGTAAAGAAAAATAGTTATATGCGATTTTAGTTGGTATACTTAAAATGAGTATATTTT

ATTATAATTATTTTATGAACTACATTTTTAAAAAATATCACTTAAAAACTAGTTATAGAAA
GTACGAAAATAAACTAATTTTAACCTGAAGAGAATTTTAATCAGTAGGGGTCACATCTA
GTACCTGGCTTTCCTACGCGCAACTGGCGCAAGCCCACCTGCCTCTCCCAATCAATCCA
CCACTGTGCGGCGTTATATAGATCGCCCGTTGCTTCCTCAATCCTCATCTCCGCAAGGTC
AATCGAGCGGGCAAAGCAAGTGCGCGCTACCGCTACACCCAGACCTCGCTTCCTCTCA
GATCTGCAGCA**ATG**GCCACCGCGACAGCCCAGACGACCGCGACGAAGGAGCAGACC
AATGGCAGCGGCGGCGAGCAGAAGACGCGCCACTCCGAGGTCGGCCACAAGAGCCT
GCTCCAGAGCGACGCGCTCTACCAGTACATCCTGGAGACGAGCGTGTACCCGCGCGAG
CACGAGTGCATGAAGGAGCTCCGCGAGGTCACCGCCAAGCACCCATGGTATGTTGCTG
TCCCCCTCCACTTCTTCTGGATCTGCGAGATCGCCGAGATTAATGTCGGATCTTGCCTG
ATCTTGTGGCTGCAGGAACCTGATGACGACGTCGGCGGACGAGGGCCAGTTCCTGAA
CATGCTGCTCAAGCTCATCGGCGCCAAGAAGACCATGGAGATCGGCGTCTATACGGGC
TACTCCCTCCTCGCCACCGCGCTCGCCATCCCCGAGGACGGCACGGTAAGTCTATCGAT
CAGCTAGCTAGTACTTCCGCCCGCACCGCAGATTCCCAGATCTGGCAAGCTGAAATGAT
CGATGGCACTCCTCGATATATGCAGATCTTGGCCATGGACATCAACCGCGAGAACTACG
AGCTCGGCTTGCCCTGCATCGAGAAGGCCGGCGTCGCCCACAAGATCGACTTCCGCGA
GGGCCCCGCCCTCCCCGTCCTCGACCAGCTTCTCGAGGACGAGAGCAACCACGGCTC
CTTCGACTTCGTCTTCGTGGACGCCGACAAGGACAACTACCTCAACTACCACGAGCGG
CTGATGAAGCTGGTCAAGGTCGGCGGCCTCGTCGGCTACGACAACACGCTCTGGAAC
GGCTCCGTCGTGCTCCCCGCCGACGCGCCCATGCGCAAGTACATCCGCTACTACCGGG
ACTTTGTGCTCGAGCTCAACAAGGCCCTCGCCGCCGACGAGCGCGTCGAAATCTGCCA
GCTCCCCGTCGGCGACGGCATCACCCTCTGCCGCCGCGCCAAG**TGA**GCCGCCCCTCCC
GCCGTGCTGGTCGCTCGACGGCGCGCCCGCACGCGCGCCATGATGCCGTCATCTGCTG
CTCCTACCATAGATGCGAACTGAAGAAGGATTAATTATCGTTTTGGCTTCCTTTTTTCTT
CTTCTTGGAAATCGTCGTTTCATATGTAAAAAAATGTCGAGACAATAATATTATAAGATG
ACCCGGTGTTTGTTAGTTTGCGTTGCGTGTGCGAATGGCGACCGCAGCTGATGAATCG
ACGATGATATACTTTTTGAAA

>DfCCoAOMT17 (Dfa0G042980)
ATTTCTAAATCAAACACTAAAAACATATGTATTTCTAAACATTCGTGTGTATTTCTAAATC
AAATCCTAAATCTAAACATTCATCAATCTCGCTCAAACCAAACCCTAAAAACATGTGTA
TCTCTAAATCAAACCCTAAATCTAAACATTCATCAATCTCGCTCAAATCAAACCTTAAA
AACATATGTATTTCTAAATCAAACCCTAAAAACATATGTATTTCTAAAAATCAAACCCTA
AAAACGAATCTATACACATTACTACTTTTACATTACGCATATCTATTGAAAATCCAACCT
AAAACCAAGCCAAATTTAACTAATTTCATCTAGGGTTTCAAATCTTCCCACACGAAATG
TACCTCAAATAATCCATATTTATTGCAAAAAATCAAGGGAAATGAGGGGGTTCACCTTG
CTTACTCGATTTGGAAGTAAAATCGCGAGAAAAAACGATGGATTTGAGTAGATTTTGA
GGGGGAAGAGAGTGGTGGCGGCGGCGGCGGTTGCTTCGGCTCGGACTGAACAGAGT
GAGGGGGTGGGGGGCTGCGCTCGGGCTTAAGTCCCAGAGCACGGCGCCACGGCCGTT
AGCGCCGTTGGTACACAGCACGACGCCATGAGCTTTGGCGCCGAGCTCTGCCATCGTG
GATCCTACGTGGCCAAGCACGGCGCCAAAGCATATGACGCCGTTCTGTGTACCTTGAG
CGCCATATACTTTGGCGCCGTGGTAAGGGTCCATTTTTGCAAATCTTTTCTCCAGGGATC
CATATGTAAAAAACTTTCAAAATTTGGGCTAACAAGTAAAAATTTTACCATATTATTCTT
CTCGAGAGCCTAGCGTGTCCACATCTCATTGACATGGGGGCCCCGCAGACGCCAAGCT

AATCTGCGTTCCCGTTTCTACTTGGGGAAAATTGCTCTCATACCACAAAGTTAAATACC
ATCCTCCCTATTGCCACATACTTAAGTGGCTTTGTTATTATGCCACAAACTTGAGTGTTT
TTCCTTTCTTTTGCCATTTCCTCCTCTTAAAGTCTATTTTAATTTTATTTTGCATTTCCCTA
ACGTGAATTGACCTTTTTGCCCTCGCTCCCCTACCTCGCCCTCAACGCTAACCTCCTTC
CCCGCCGGCTGCCAACTCCCCCCCCCCCGCCTCCCCTCCATCCACGCCACCGTCTCCC
TCTTCAGCTCCGCCTCTGGGGTCCCCCTCGCCTCCGTCGACGGCTCCGCGCTCACCCTC
CTCCGCACCGCCGCCGTCTCCGCGCTCGCCGCATCCCCCATCTCCCCCGACCGGCCCC
CCTGCACCCTCCCCCACCCGCATCGCCGGCGTGAAGGACCCGACCAGGTCCAGGTGC
GCCCCGGCCGCAGCTGTTCCCCCGCACGATCGGCTCCTACGACCCCATCGCGCAGCTC
ACGACGTCCGCCGCGGCCACCGCCTTGTCCATGCCGTCGACCTCCTCGACGGTGATCC
TCGGGTGCGCATCCCGAAGCTTCGAGGCGAGCACGGTGGATTTGGCCCTGGTGCGGTT
CCAGACGAGGATGCGGGAGATGGAGGAGAGGGCGGCCAGGTGGGCTTCGGCGAGGT
AGGGCGCGAGGGCGCCAGCGGGACGACGAGGCGCTCCGGTGGGGGAGGGTGCAGGG
GGGCCGGTCGGGGGAGATGAGGGATGCGGTGAGCGTGGAGACGGCGGCGGTGCGGA
GGAGGGTGAGCGCGGAGCCGTCGACGGAGGCGAGGGGGACCCCAGAGGCGGAGCTG
AAGAGGGAGACGGCGGCGTGGATGGAGGGGAGGCGGGGGGAGTTGGCGGCCGGCGG
GGAAGGAGGCTAGCGTTGAGGGCGAGGTAGGGGAGCGAGGGCAAAAAGGTCAATTC
AATGCAAAATAAAATTAAAATAGACTTTAAGACGAGGAAATGGCAAAAGAAAGGAAA
AAACACTCAAATTTGTGGCATAATAACAAAACCACTTAAGTATGTGGCAAAAGGGAGG
ATGGTATTTAAGTTTGTGGT**ATG**AGAGCAATTTTCCCTTCTACTTGGCGGCTGCCTCCC
GCGACGATGCCTCGCTTCGCCGTCGCCTCCTCCTCCCTTCCTCCGGCGGCGGCGGCGG
CGGCTGCTCTCGGGTTCGGCGCCACGCCTCCTTGCGTCCTCCAAGCCCAAACCCTAGC
CGCCGCCGCTCCGCCCCCGCCATGGCGCCGCCGCCTGTGCATGTGCTCCTCCTCCTCG
ACAGCGGCGGCGGTGGAGGAGGCGCGGCGCGGGCGGAAGCAGCTGGGAATGACACC
GCAGCTCTACGACTACCTCCTCGCCAATGTCCGCGAGCACCCCGTACGCCACTCCCCCT
CTCTTTGTGTATATCTCTATCGTTTTGAACCAGTTGCTGCAATTTGGGGGGAATTTATTTT
GCTAGGTCCTGACCCCTGACCAATGTGCGTGTGTTGTTTATCCTACTCTGGATACGCTTT
TTAGATCCTTCGGGAGCTTCGCGAAGAGACGGCGGCCATGAGAGGAAGCCAGATGCA
GGTACATTAGCATACATAAACTTAGAATACTACGCTTTAGAATTTAGATGGAGAGTGTAA
CGCCCCATTTCTGAGATCGTTGGATTTCCCATTGCTGAGACACGGCCCCATTTTTGAGG
TCGTTGGATTTCCCCGTAGCTGAGTGTGCATCAAATGTGCTCATGCTTTATTCATAAAAG
CCACACTTAGTACAGTAGATTATAGCATGTCAGCCAAGTTTACAATAAAAAGGGCGTCC
AACCTCAAAATTTATATTTCATCACAAAAGAACTTCTAAAAGATCCACAAGACGTAAAA
CCAGTAATATCTCCTAGGGAATCATCAAAGTCTTCTTCGAAAGGCTCTTCATTGACTTTC
GGAGTTTCTTTTGTTGATATTTTAGCAAGTATGAGTACGACCGGCGCCTCAGCGGGGTG
GCACGACTTCGCCGTCCGCCTCGTATGCATTGCCTCGACGGGACAGCATGCCTTTGCCA
GGAGACTTCGCCACCCGGCTTGGAGGCATCGCTTCGGCGGGGCCGCATGACTTCGTCG
CCCTCCTTGTAGTCATCGCCTCGGCGGGATGGCCTGGCTTCGGCATAAGACTTCGCCGC
CTGCCTTGGAGGCATCGTCTCGACGGGGCGGCACGATGTCGACCACCTCGTACGCATC
ACCTCGGAGGGACGTACCGGTTTTGGCGTGAGACTTCGCCGACCTCGTAGACGTTGCG
TCGGAGGGACGAAGCCACCCCTGCTCTGCTTCCATAATTTCTTGGTGGACTCTGCAGTC
GCATTATGATCAGTAAAAGAAAAACCCTCTTCCAGGGGCTGCAAGCTATCAGTTGCATC
GTCTCACCAGTCAGCACCCTTGCGCTTACTATTGGGGGACGGAGGGCTGGGATAGTTT
GTCACGTCAGCTCGCCCATCAGGACCTCCGCGCTTGCTATTGAGAGAGTTCTGTTGTAG

AGGTGGTCCAACCCCCTGTGACTGTGCCTTGCAACTGGCTTAATATGTAATAGGCTCAA
TAGTATAAAGGAATATACCAATGTACTCCTACCTTATGTAATTATTAAGGGATAGGAAGA
ATTTCTCATCCTACTCCACTTATATATATAAACCCCCAAGAGGGGCATGAGGAGGGGAG
GTCCACTCATTTCCCACATACCATCTCTTTGTTGGCTCTCCTCTTCTAGGCTCCCTCTTTA
CTATTTGGTCTGTATCCATAACATCTAATACTCTTTTAGTGTCGTTTTCTTGTTTTTTTCCC
TAGAATAGTCTTAATTATTTCGTAGGTATATCCCAAAAAAGAAATCCAATTTCACATATTT
ATAATTGTTTTTTCTGAAAATATAATGATAGTCCCTGGATACATTACCAAAATTCTGTATA
ATGATAGTCACTTTCAGCTGGTTGTTTTGTTTTGGTAGGTTTCTCCCGCACAGGCTCAG
CTGTTAGCGATCCTTGCGCAAACTCTTGGAGCACAGCGATGTATTGAAGTCGGCATCTA
TACTGTATGTGAACCTAGCGTTTTGTAATTTAGATGTTCCTACCTTCCACTCAATCAGTA
CTCAGTAAACACTAAATAGTACTAAGTTATTGTTTCTTCTTGGAACCATAGCATGCTATA
TTGATGTTATGTTAGTTGTTAACATGTTGTCCTAGCACACCAAACATAGAATCCACACAT
ATGTGCGAGACAAATAAGAAGTATATATGAGTGGGTTAACAAATCATCTGTAACATCTC
TGGTATCTGTCATTTTCCCTGGAAGTGATAGCATAATAGGCACTGCATCTTTCCAAGGTA
TATACCCAGTAAGATTTTAAATAGTACTTTCAGAATGCTGGTACTCTCAGAATGCTGAAG
CCTGAATCTTGCATATGTTTTCCTTCCTTCAAACAATATTTTCTGTCAGGGACAATAATG
TAATGCATGATGTATTGTTTATGTCAACTTGTCAAGTGCTGGAATCATTAGCTACTTTTGT
ACAAAAGCAACAATCATTGCAAATTTGGTAATCTTATCTTCTCACTCCTTTAATCTTTTC
ACAGGGTTATTCATCATTAGCTGTAGCACTAGCACTTCCTGAATCAGGTCGTTTGGTTG
CTTGTGAAAGGGACGAAAGATGTCTAGAAGTTGCCAAGAAATATTATCAGCGCGCTGG
TGTTGCACACAAGGTGATTAATATATGTCTCTTCTTGCTTGATGAGGTCGGTTCATTATA
ATCAATGAACTACATAATTTTCTGTATTTGTAGTTTACTTCTAGATTCTTGGCTAGAAATT
TACAATCAATTGCATGTGATGTTTCAGTAGGTTGGAGAACTTGTTTTTGATACAAAGCT
AAAATTCTTCCAAGTCCCAAGTTCCCAACCATAAAGATCATGGAAAAAAATTGTTCA
GAAGCAGGGCAAAGCCTTCTTTTTAGTTTCACTAATTCCCCCTTTATGTATTATTTATTTT
ACATCATTGCTCTTGTATTTGTTTCACTTTTTACTGACTTATGTAGCACACTATAGTTTAT
GAAATTAGGAGTGCCGCCATTTAATGCATCACATTTATTTTGTAGGGAAACTAATCTGTG
TAGGTTTAATCTATATTTATTTCCTCTTTAATGATTTGTGAGCACTTAAAACTGAAATGTT
GACCTTTTTTTGGAATGCCTAGAATGTATAGCATATTAGTATTCAAGGACCGATTTGTATT
CTTGCATATTAGATTTTCTATGCTGATCTGTCTTAAAATTGCTTAGGTTGATGTCAAGCAC
TCTTTAGCTGTAGATTCCCTAAAGTTGTTGCTCGATTGTGGTGAAGCTTTCAGGTATAGT
AATTTGGTATGGTATAATCCATCATTATATTAGTTGACCTCTTGCATGCTCTAACTAATCT
TTTCCTTTTCTGCTTTTTTCTAGTTATGACTTTGCATTCGTTGATGCAGACAAGAGAATG
TATGAGGAATATTTTGAACTTCTACTTAAGCTAGTAAGATTCAACATGAAGTTTACCTTG
TGGGTTATTTATAACTCATTGTGGTTTACAACGCATGTTTTCACTCTCTGTTCCCCATAGT
GTTTTTTTCATAATATTATAGAAGATTCCCCATTCTTGCCATAAGTTTTATGATTTTATCTA
TACTTTCCGTACATAGTGATCGTAGTACTTATGCTTTTTATATGTTGAACATTATGTTTTTG
TTGTGTTGTAGGTAAGAGTTGGTGGTTTAATTGTAATGGACAATGTCCTTTGGTACGGA
AGGGTTGCTGATCCGCTAGTAAGTTCTGTTCTCTTTTTGAGTTTTGACATGATAAAGATG
TTACAGGGTAGACATTAGGTAACACTTTTCTCTGGCATTTTTTCCTTAGGTGAATGACGC
AAAGACAATCAGTATAAGGAACTTCAATAAGAAACTTTTGGAGGATAAACGTGTCAAT
ATCAGCATGGTCTCTTCCTCTCTCTCTACCTCTCATATGTTTGGTCTACCACATAGTTATT
AGTCATTACTTGCGAATGTTGATTGTGAAGCCTTAGAAAATATTTTATATGTCTGCATATT
TATTCTGTCGCCTAGAGCATGTGAAGAGCACCAACAATGATGTGTAGCGGCACTGCAAT

GCCAGCACAGTAAATATCCCAAATTCGAAATTTTTATTTGCCTAATAATGTGGCACATTC
AAGCCATGGTCATGTATACCTGTTTGGATGGGGGGTATGGATTCGAATCCTTAGGATTTA
GTTCAAATTTGCACTAAATCCCAAAAATTCACAAGGAAATCCCCCTCATCCAAACACCC
CCTAAATGTGTTGTCCTCTTCACTATCTTTATAGTTGTTCAACTGCTTCAGAACCTTTATA
ACCCCCTGTATCCAAACCTTCTTTTGATACACCATCGTTTGGTTTTAGTACAACTTCTGT
GAGAAGAAAACGGTATGTGTAACATTGCTCCAATTACAAGTGTATTCTTGTAACCGTAT
GCAATGAGAACCATAATATTGGTACCATTTAGATTTTCATTTGACATGCTTATATCATGAT
TCAGTGCATCATATCCCATGCCCTTCTCATGTGTTGTCTGTTCATACCACCTGTCTGTGC
TTTAGTACTGGATCTTAATTAACAATGGTATTATATTTTTGAGAAATTTGCAGGTGCCAAT
TGGGGACGGGATGACAATTTGTCGGAAGCTAGTAGATACTCCCCTTCCGAGGTCAAGC
TCTCAA**TGA**
