# Supplementary figures and images for: Genome-Wide Identification and Expression Analysis of Dendrocalamus farinosus CCoAOMT Gene Family and the Role of DfCCoAOMT14 Involved in Lignin Synthesis
Source: Int J Mol Sci. 2023 May 18;24(10):8965. doi: 10.3390/ijms24108965 (PMC10219071; doi:10.3390/ijms24108965)

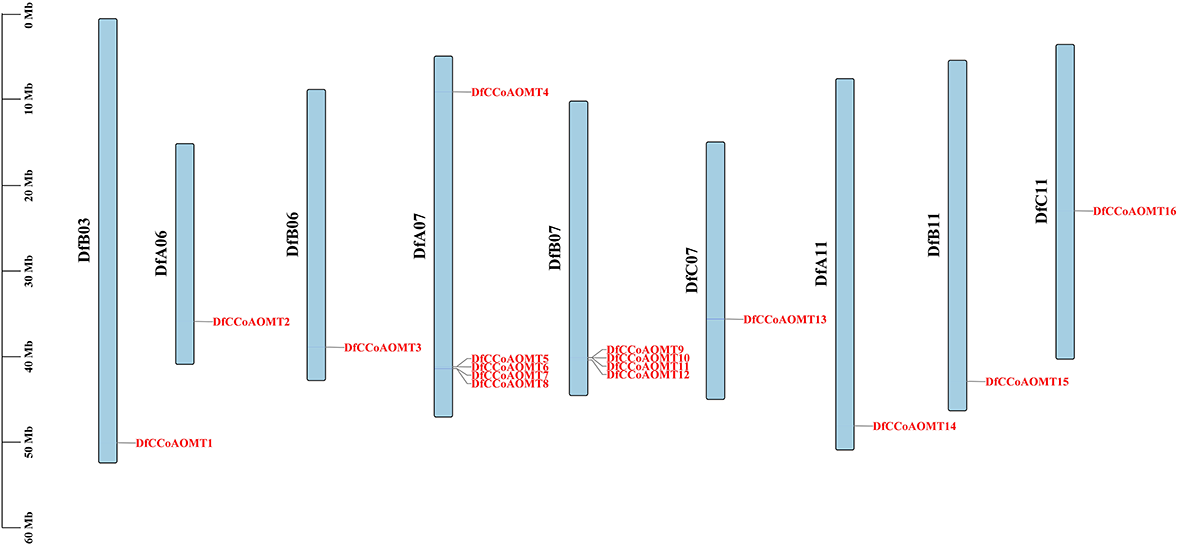

Supplement: Supplementary file 1 [file ijms-24-08965-s001.zip › Supplementary figure S1-Chromosome Localization of DfCCoAOMT.tif]

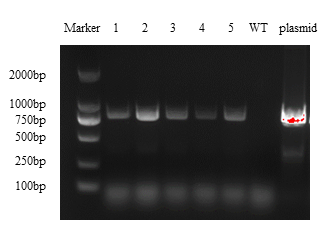

Supplement: Supplementary file 1 [file ijms-24-08965-s001.zip › Supplementary figure S2-Positive verification of DfCCoAOMT14 on tobacco DNA genome.tif]
